# Supplementary material for: Genome-wide identification, characterization and gene expression of BES1 transcription factor family in grapevine (Vitis vinifera L.)
Source: Sci Rep. 2023 Jan 5;13:240. doi: 10.1038/s41598-022-24407-y (PMC9816167; doi:10.1038/s41598-022-24407-y)
Supplement: Supplementary file 3 — Supplementary Information. [file 41598_2022_24407_MOESM3_ESM.zip › Vvi_Atr/Vitis_vinifera.PN40024.v4.dna_sm.toplevel.fa.vs.Amborella_trichopoda.AMTR1.0.dna_sm.toplevel.fa.html/Vvi-12.html]

|  |  |  |  |  |  |  |  |  |  |  |  |  |  |
| --- | --- | --- | --- | --- | --- | --- | --- | --- | --- | --- | --- | --- | --- |
| Duplication depth | Reference chromosome | Collinear blocks | | | | | | | | | | | |
| 0 | Vvi-Vitvi12g04000\_t001 |  |  |  |  |  |  |  |  |  |  |  |  |
| 0 | Vvi-Vitvi12g04001\_t001 |  |  |  |  |  |  |  |  |  |  |  |  |
| 0 | Vvi-Vitvi12g00001\_t001 |  |  |  |  |  |  |  |  |  |  |  |  |
| 0 | Vvi-Vitvi12g00002\_t001 |  |  |  |  |  |  |  |  |  |  |  |  |
| 0 | Vvi-Vitvi12g00003\_t001 |  |  |  |  |  |  |  |  |  |  |  |  |
| 0 | Vvi-Vitvi12g02179\_t001 |  |  |  |  |  |  |  |  |  |  |  |  |
| 0 | Vvi-Vitvi12g00005\_t003 |  |  |  |  |  |  |  |  |  |  |  |  |
| 0 | Vvi-Vitvi12g00006\_t001 |  |  |  |  |  |  |  |  |  |  |  |  |
| 0 | Vvi-Vitvi12g04002\_t001 |  |  |  |  |  |  |  |  |  |  |  |  |
| 0 | Vvi-Vitvi12g00008\_t001 |  |  |  |  |  |  |  |  |  |  |  |  |
| 0 | Vvi-Vitvi12g00009\_t001 |  |  |  |  |  |  |  |  |  |  |  |  |
| 0 | Vvi-Vitvi12g02180\_t001 |  |  |  |  |  |  |  |  |  |  |  |  |
| 0 | Vvi-Vitvi12g04003\_t001 |  |  |  |  |  |  |  |  |  |  |  |  |
| 0 | Vvi-Vitvi12g00011\_t001 |  |  |  |  |  |  |  |  |  |  |  |  |
| 0 | Vvi-Vitvi12g00014\_t001 |  |  |  |  |  |  |  |  |  |  |  |  |
| 0 | Vvi-Vitvi12g00015\_t001 |  |  |  |  |  |  |  |  |  |  |  |  |
| 0 | Vvi-Vitvi12g04004\_t001 |  |  |  |  |  |  |  |  |  |  |  |  |
| 0 | Vvi-Vitvi12g04005\_t001 |  |  |  |  |  |  |  |  |  |  |  |  |
| 0 | Vvi-Vitvi12g04006\_t001 |  |  |  |  |  |  |  |  |  |  |  |  |
| 0 | Vvi-Vitvi12g00017\_t001 |  |  |  |  |  |  |  |  |  |  |  |  |
| 0 | Vvi-Vitvi12g04007\_t001 |  |  |  |  |  |  |  |  |  |  |  |  |
| 0 | Vvi-Vitvi12g00019\_t002 |  |  |  |  |  |  |  |  |  |  |  |  |
| 0 | Vvi-Vitvi12g00020\_t002 |  |  |  |  |  |  |  |  |  |  |  |  |
| 0 | Vvi-Vitvi12g00021\_t001 |  |  |  |  |  |  |  |  |  |  |  |  |
| 0 | Vvi-Vitvi12g00022\_t001 |  |  |  |  |  |  |  |  |  |  |  |  |
| 0 | Vvi-Vitvi12g00024\_t001 |  |  |  |  |  |  |  |  |  |  |  |  |
| 0 | Vvi-Vitvi12g00025\_t001 |  |  |  |  |  |  |  |  |  |  |  |  |
| 0 | Vvi-Vitvi12g04008\_t001 |  |  |  |  |  |  |  |  |  |  |  |  |
| 0 | Vvi-Vitvi12g04009\_t001 |  |  |  |  |  |  |  |  |  |  |  |  |
| 0 | Vvi-Vitvi12g04010\_t001 |  |  |  |  |  |  |  |  |  |  |  |  |
| 0 | Vvi-Vitvi12g00027\_t001 |  |  |  |  |  |  |  |  |  |  |  |  |
| 0 | Vvi-Vitvi12g04011\_t001 |  |  |  |  |  |  |  |  |  |  |  |  |
| 0 | Vvi-Vitvi12g00029\_t001 |  |  |  |  |  |  |  |  |  |  |  |  |
| 0 | Vvi-Vitvi12g00031\_t001 |  |  |  |  |  |  |  |  |  |  |  |  |
| 0 | Vvi-Vitvi12g02184\_t001 |  |  |  |  |  |  |  |  |  |  |  |  |
| 0 | Vvi-Vitvi12g02185\_t001 |  |  |  |  |  |  |  |  |  |  |  |  |
| 0 | Vvi-Vitvi12g04012\_t001 |  |  |  |  |  |  |  |  |  |  |  |  |
| 0 | Vvi-Vitvi12g02186\_t001 |  |  |  |  |  |  |  |  |  |  |  |  |
| 0 | Vvi-Vitvi12g00032\_t001 |  |  |  |  |  |  |  |  |  |  |  |  |
| 0 | Vvi-Vitvi12g00033\_t001 |  |  |  |  |  |  |  |  |  |  |  |  |
| 0 | Vvi-Vitvi12g00034\_t002 |  |  |  |  |  |  |  |  |  |  |  |  |
| 0 | Vvi-Vitvi12g04013\_t001 |  |  |  |  |  |  |  |  |  |  |  |  |
| 0 | Vvi-Vitvi12g00036\_t001 |  |  |  |  |  |  |  |  |  |  |  |  |
| 0 | Vvi-Vitvi12g00037\_t001 |  |  |  |  |  |  |  |  |  |  |  |  |
| 0 | Vvi-Vitvi12g00038\_t001 |  |  |  |  |  |  |  |  |  |  |  |  |
| 0 | Vvi-Vitvi12g00039\_t001 |  |  |  |  |  |  |  |  |  |  |  |  |
| 0 | Vvi-Vitvi12g00040\_t001 |  |  |  |  |  |  |  |  |  |  |  |  |
| 0 | Vvi-Vitvi12g00041\_t001 |  |  |  |  |  |  |  |  |  |  |  |  |
| 0 | Vvi-Vitvi12g02191\_t001 |  |  |  |  |  |  |  |  |  |  |  |  |
| 0 | Vvi-Vitvi12g04014\_t002 |  |  |  |  |  |  |  |  |  |  |  |  |
| 0 | Vvi-Vitvi12g02192\_t001 |  |  |  |  |  |  |  |  |  |  |  |  |
| 0 | Vvi-Vitvi12g00044\_t001 |  |  |  |  |  |  |  |  |  |  |  |  |
| 0 | Vvi-Vitvi12g00045\_t001 |  |  |  |  |  |  |  |  |  |  |  |  |
| 0 | Vvi-Vitvi12g00046\_t001 |  |  |  |  |  |  |  |  |  |  |  |  |
| 0 | Vvi-Vitvi12g00048\_t001 |  |  |  |  |  |  |  |  |  |  |  |  |
| 0 | Vvi-Vitvi12g00049\_t001 |  |  |  |  |  |  |  |  |  |  |  |  |
| 0 | Vvi-Vitvi12g00050\_t001 |  |  |  |  |  |  |  |  |  |  |  |  |
| 0 | Vvi-Vitvi12g00053\_t001 |  |  |  |  |  |  |  |  |  |  |  |  |
| 0 | Vvi-Vitvi12g00054\_t001 |  |  |  |  |  |  |  |  |  |  |  |  |
| 0 | Vvi-Vitvi12g00055\_t001 |  |  |  |  |  |  |  |  |  |  |  |  |
| 0 | Vvi-Vitvi12g00056\_t001 |  |  |  |  |  |  |  |  |  |  |  |  |
| 0 | Vvi-Vitvi12g00058\_t001 |  |  |  |  |  |  |  |  |  |  |  |  |
| 0 | Vvi-Vitvi12g00059\_t001 |  |  |  |  |  |  |  |  |  |  |  |  |
| 0 | Vvi-Vitvi12g02193\_t001 |  |  |  |  |  |  |  |  |  |  |  |  |
| 0 | Vvi-Vitvi12g02194\_t001 |  |  |  |  |  |  |  |  |  |  |  |  |
| 0 | Vvi-Vitvi12g02195\_t001 |  |  |  |  |  |  |  |  |  |  |  |  |
| 0 | Vvi-Vitvi12g00060\_t001 |  |  |  |  |  |  |  |  |  |  |  |  |
| 0 | Vvi-Vitvi12g00061\_t001 |  |  |  |  |  |  |  |  |  |  |  |  |
| 0 | Vvi-Vitvi12g00062\_t001 |  |  |  |  |  |  |  |  |  |  |  |  |
| 0 | Vvi-Vitvi12g02197\_t002 |  |  |  |  |  |  |  |  |  |  |  |  |
| 0 | Vvi-Vitvi12g02198\_t001 |  |  |  |  |  |  |  |  |  |  |  |  |
| 0 | Vvi-Vitvi12g00064\_t001 |  |  |  |  |  |  |  |  |  |  |  |  |
| 0 | Vvi-Vitvi12g00065\_t001 |  |  |  |  |  |  |  |  |  |  |  |  |
| 0 | Vvi-Vitvi12g00066\_t001 |  |  |  |  |  |  |  |  |  |  |  |  |
| 0 | Vvi-Vitvi12g00067\_t003 |  |  |  |  |  |  |  |  |  |  |  |  |
| 0 | Vvi-Vitvi12g00068\_t001 |  |  |  |  |  |  |  |  |  |  |  |  |
| 0 | Vvi-Vitvi12g04015\_t001 |  |  |  |  |  |  |  |  |  |  |  |  |
| 0 | Vvi-Vitvi12g02202\_t001 |  |  |  |  |  |  |  |  |  |  |  |  |
| 0 | Vvi-Vitvi12g00071\_t001 |  |  |  |  |  |  |  |  |  |  |  |  |
| 0 | Vvi-Vitvi12g00073\_t001 |  |  |  |  |  |  |  |  |  |  |  |  |
| 0 | Vvi-Vitvi12g00075\_t001 |  |  |  |  |  |  |  |  |  |  |  |  |
| 0 | Vvi-Vitvi12g00076\_t001 |  |  |  |  |  |  |  |  |  |  |  |  |
| 0 | Vvi-Vitvi12g04016\_t001 |  |  |  |  |  |  |  |  |  |  |  |  |
| 0 | Vvi-Vitvi12g00080\_t001 |  |  |  |  |  |  |  |  |  |  |  |  |
| 0 | Vvi-Vitvi12g00081\_t001 |  |  |  |  |  |  |  |  |  |  |  |  |
| 0 | Vvi-Vitvi12g00082\_t001 |  |  |  |  |  |  |  |  |  |  |  |  |
| 0 | Vvi-Vitvi12g04017\_t001 |  |  |  |  |  |  |  |  |  |  |  |  |
| 0 | Vvi-Vitvi12g04018\_t001 |  |  |  |  |  |  |  |  |  |  |  |  |
| 0 | Vvi-Vitvi12g04019\_t001 |  |  |  |  |  |  |  |  |  |  |  |  |
| 0 | Vvi-Vitvi12g00084\_t001 |  |  |  |  |  |  |  |  |  |  |  |  |
| 0 | Vvi-Vitvi12g02204\_t001 |  |  |  |  |  |  |  |  |  |  |  |  |
| 0 | Vvi-Vitvi12g00085\_t001 |  |  |  |  |  |  |  |  |  |  |  |  |
| 0 | Vvi-Vitvi12g00086\_t001 |  |  |  |  |  |  |  |  |  |  |  |  |
| 0 | Vvi-Vitvi12g04020\_t001 |  |  |  |  |  |  |  |  |  |  |  |  |
| 0 | Vvi-Vitvi12g00088\_t001 |  |  |  |  |  |  |  |  |  |  |  |  |
| 0 | Vvi-Vitvi12g04021\_t001 |  |  |  |  |  |  |  |  |  |  |  |  |
| 0 | Vvi-Vitvi12g00092\_t001 |  |  |  |  |  |  |  |  |  |  |  |  |
| 0 | Vvi-Vitvi12g00093\_t001 |  |  |  |  |  |  |  |  |  |  |  |  |
| 0 | Vvi-Vitvi12g04022\_t001 |  |  |  |  |  |  |  |  |  |  |  |  |
| 0 | Vvi-Vitvi12g00095\_t001 |  |  |  |  |  |  |  |  |  |  |  |  |
| 0 | Vvi-Vitvi12g04023\_t001 |  |  |  |  |  |  |  |  |  |  |  |  |
| 0 | Vvi-Vitvi12g00096\_t001 |  |  |  |  |  |  |  |  |  |  |  |  |
| 0 | Vvi-Vitvi12g04024\_t001 |  |  |  |  |  |  |  |  |  |  |  |  |
| 0 | Vvi-Vitvi12g00097\_t001 |  |  |  |  |  |  |  |  |  |  |  |  |
| 0 | Vvi-Vitvi12g00098\_t001 |  |  |  |  |  |  |  |  |  |  |  |  |
| 0 | Vvi-Vitvi12g00099\_t001 |  |  |  |  |  |  |  |  |  |  |  |  |
| 0 | Vvi-Vitvi12g00101\_t001 |  |  |  |  |  |  |  |  |  |  |  |  |
| 0 | Vvi-Vitvi12g00102\_t001 |  |  |  |  |  |  |  |  |  |  |  |  |
| 0 | Vvi-Vitvi12g00103\_t001 |  |  |  |  |  |  |  |  |  |  |  |  |
| 0 | Vvi-Vitvi12g00104\_t001 |  |  |  |  |  |  |  |  |  |  |  |  |
| 0 | Vvi-Vitvi12g00105\_t001 |  |  |  |  |  |  |  |  |  |  |  |  |
| 0 | Vvi-Vitvi12g00106\_t001 |  |  |  |  |  |  |  |  |  |  |  |  |
| 0 | Vvi-Vitvi12g04025\_t001 |  |  |  |  |  |  |  |  |  |  |  |  |
| 0 | Vvi-Vitvi12g02210\_t001 |  |  |  |  |  |  |  |  |  |  |  |  |
| 0 | Vvi-Vitvi12g00108\_t001 |  |  |  |  |  |  |  |  |  |  |  |  |
| 0 | Vvi-Vitvi12g00109\_t001 |  |  |  |  |  |  |  |  |  |  |  |  |
| 0 | Vvi-Vitvi12g00111\_t001 |  |  |  |  |  |  |  |  |  |  |  |  |
| 0 | Vvi-Vitvi12g04026\_t001 |  |  |  |  |  |  |  |  |  |  |  |  |
| 0 | Vvi-Vitvi12g02211\_t001 |  |  |  |  |  |  |  |  |  |  |  |  |
| 0 | Vvi-Vitvi12g02212\_t001 |  |  |  |  |  |  |  |  |  |  |  |  |
| 0 | Vvi-Vitvi12g00112\_t001 |  |  |  |  |  |  |  |  |  |  |  |  |
| 0 | Vvi-Vitvi12g04027\_t001 |  |  |  |  |  |  |  |  |  |  |  |  |
| 0 | Vvi-Vitvi12g04028\_t001 |  |  |  |  |  |  |  |  |  |  |  |  |
| 0 | Vvi-Vitvi12g02214\_t001 |  |  |  |  |  |  |  |  |  |  |  |  |
| 0 | Vvi-Vitvi12g00114\_t002 |  |  |  |  |  |  |  |  |  |  |  |  |
| 0 | Vvi-Vitvi12g00115\_t001 |  |  |  |  |  |  |  |  |  |  |  |  |
| 0 | Vvi-Vitvi12g02215\_t001 |  |  |  |  |  |  |  |  |  |  |  |  |
| 0 | Vvi-Vitvi12g00116\_t001 |  |  |  |  |  |  |  |  |  |  |  |  |
| 0 | Vvi-Vitvi12g04029\_t001 |  |  |  |  |  |  |  |  |  |  |  |  |
| 0 | Vvi-Vitvi12g00117\_t001 |  |  |  |  |  |  |  |  |  |  |  |  |
| 0 | Vvi-Vitvi12g00118\_t001 |  |  |  |  |  |  |  |  |  |  |  |  |
| 0 | Vvi-Vitvi12g00119\_t001 |  |  |  |  |  |  |  |  |  |  |  |  |
| 0 | Vvi-Vitvi12g00120\_t002 |  |  |  |  |  |  |  |  |  |  |  |  |
| 0 | Vvi-Vitvi12g02216\_t001 |  |  |  |  |  |  |  |  |  |  |  |  |
| 0 | Vvi-Vitvi12g00121\_t001 |  |  |  |  |  |  |  |  |  |  |  |  |
| 0 | Vvi-Vitvi12g04030\_t001 |  |  |  |  |  |  |  |  |  |  |  |  |
| 0 | Vvi-Vitvi12g00122\_t001 |  |  |  |  |  |  |  |  |  |  |  |  |
| 0 | Vvi-Vitvi12g02217\_t001 |  |  |  |  |  |  |  |  |  |  |  |  |
| 0 | Vvi-Vitvi12g02218\_t001 |  |  |  |  |  |  |  |  |  |  |  |  |
| 0 | Vvi-Vitvi12g04031\_t001 |  |  |  |  |  |  |  |  |  |  |  |  |
| 0 | Vvi-Vitvi12g04032\_t001 |  |  |  |  |  |  |  |  |  |  |  |  |
| 0 | Vvi-Vitvi12g04033\_t001 |  |  |  |  |  |  |  |  |  |  |  |  |
| 0 | Vvi-Vitvi12g00124\_t001 |  |  |  |  |  |  |  |  |  |  |  |  |
| 0 | Vvi-Vitvi12g04034\_t001 |  |  |  |  |  |  |  |  |  |  |  |  |
| 0 | Vvi-Vitvi12g00127\_t001 |  |  |  |  |  |  |  |  |  |  |  |  |
| 0 | Vvi-Vitvi12g00128\_t001 |  |  |  |  |  |  |  |  |  |  |  |  |
| 0 | Vvi-Vitvi12g00130\_t001 |  |  |  |  |  |  |  |  |  |  |  |  |
| 0 | Vvi-Vitvi12g04035\_t001 |  |  |  |  |  |  |  |  |  |  |  |  |
| 0 | Vvi-Vitvi12g04036\_t001 |  |  |  |  |  |  |  |  |  |  |  |  |
| 0 | Vvi-Vitvi12g00131\_t001 |  |  |  |  |  |  |  |  |  |  |  |  |
| 0 | Vvi-Vitvi12g04037\_t001 |  |  |  |  |  |  |  |  |  |  |  |  |
| 0 | Vvi-Vitvi12g04038\_t001 |  |  |  |  |  |  |  |  |  |  |  |  |
| 0 | Vvi-Vitvi12g02222\_t001 |  |  |  |  |  |  |  |  |  |  |  |  |
| 0 | Vvi-Vitvi12g02223\_t001 |  |  |  |  |  |  |  |  |  |  |  |  |
| 0 | Vvi-Vitvi12g04039\_t001 |  |  |  |  |  |  |  |  |  |  |  |  |
| 0 | Vvi-Vitvi12g00135\_t001 |  |  |  |  |  |  |  |  |  |  |  |  |
| 0 | Vvi-Vitvi12g02227\_t001 |  |  |  |  |  |  |  |  |  |  |  |  |
| 0 | Vvi-Vitvi12g04040\_t001 |  |  |  |  |  |  |  |  |  |  |  |  |
| 0 | Vvi-Vitvi12g00136\_t001 |  |  |  |  |  |  |  |  |  |  |  |  |
| 0 | Vvi-Vitvi12g00137\_t001 |  |  |  |  |  |  |  |  |  |  |  |  |
| 0 | Vvi-Vitvi12g02228\_t001 |  |  |  |  |  |  |  |  |  |  |  |  |
| 0 | Vvi-Vitvi12g00138\_t001 |  |  |  |  |  |  |  |  |  |  |  |  |
| 0 | Vvi-Vitvi12g02229\_t001 |  |  |  |  |  |  |  |  |  |  |  |  |
| 0 | Vvi-Vitvi12g04041\_t001 |  |  |  |  |  |  |  |  |  |  |  |  |
| 0 | Vvi-Vitvi12g00141\_t002 |  |  |  |  |  |  |  |  |  |  |  |  |
| 0 | Vvi-Vitvi12g00144\_t001 |  |  |  |  |  |  |  |  |  |  |  |  |
| 0 | Vvi-Vitvi12g04042\_t001 |  |  |  |  |  |  |  |  |  |  |  |  |
| 0 | Vvi-Vitvi12g02230\_t001 |  |  |  |  |  |  |  |  |  |  |  |  |
| 0 | Vvi-Vitvi12g00145\_t001 |  |  |  |  |  |  |  |  |  |  |  |  |
| 0 | Vvi-Vitvi12g04043\_t001 |  |  |  |  |  |  |  |  |  |  |  |  |
| 0 | Vvi-Vitvi12g02231\_t001 |  |  |  |  |  |  |  |  |  |  |  |  |
| 0 | Vvi-Vitvi12g00146\_t001 |  |  |  |  |  |  |  |  |  |  |  |  |
| 0 | Vvi-Vitvi12g00148\_t001 |  |  |  |  |  |  |  |  |  |  |  |  |
| 0 | Vvi-Vitvi12g00149\_t001 |  |  |  |  |  |  |  |  |  |  |  |  |
| 0 | Vvi-Vitvi12g02233\_t001 |  |  |  |  |  |  |  |  |  |  |  |  |
| 0 | Vvi-Vitvi12g00150\_t001 |  |  |  |  |  |  |  |  |  |  |  |  |
| 0 | Vvi-Vitvi12g00151\_t001 |  |  |  |  |  |  |  |  |  |  |  |  |
| 0 | Vvi-Vitvi12g02234\_t002 |  |  |  |  |  |  |  |  |  |  |  |  |
| 0 | Vvi-Vitvi12g00153\_t001 |  |  |  |  |  |  |  |  |  |  |  |  |
| 0 | Vvi-Vitvi12g02235\_t001 |  |  |  |  |  |  |  |  |  |  |  |  |
| 0 | Vvi-Vitvi12g00154\_t001 |  |  |  |  |  |  |  |  |  |  |  |  |
| 0 | Vvi-Vitvi12g00155\_t001 |  |  |  |  |  |  |  |  |  |  |  |  |
| 0 | Vvi-Vitvi12g00157\_t001 |  |  |  |  |  |  |  |  |  |  |  |  |
| 0 | Vvi-Vitvi12g00158\_t001 |  |  |  |  |  |  |  |  |  |  |  |  |
| 0 | Vvi-Vitvi12g04044\_t001 |  |  |  |  |  |  |  |  |  |  |  |  |
| 0 | Vvi-Vitvi12g02237\_t001 |  |  |  |  |  |  |  |  |  |  |  |  |
| 0 | Vvi-Vitvi12g04045\_t001 |  |  |  |  |  |  |  |  |  |  |  |  |
| 0 | Vvi-Vitvi12g00159\_t001 |  |  |  |  |  |  |  |  |  |  |  |  |
| 0 | Vvi-Vitvi12g04046\_t001 |  |  |  |  |  |  |  |  |  |  |  |  |
| 0 | Vvi-Vitvi12g02239\_t001 |  |  |  |  |  |  |  |  |  |  |  |  |
| 0 | Vvi-Vitvi12g02240\_t001 |  |  |  |  |  |  |  |  |  |  |  |  |
| 0 | Vvi-Vitvi12g02241\_t001 |  |  |  |  |  |  |  |  |  |  |  |  |
| 0 | Vvi-Vitvi12g00160\_t001 |  |  |  |  |  |  |  |  |  |  |  |  |
| 0 | Vvi-Vitvi12g02242\_t001 |  |  |  |  |  |  |  |  |  |  |  |  |
| 0 | Vvi-Vitvi12g00161\_t001 |  |  |  |  |  |  |  |  |  |  |  |  |
| 0 | Vvi-Vitvi12g02243\_t001 |  |  |  |  |  |  |  |  |  |  |  |  |
| 0 | Vvi-Vitvi12g02244\_t001 |  |  |  |  |  |  |  |  |  |  |  |  |
| 0 | Vvi-Vitvi12g02245\_t001 |  |  |  |  |  |  |  |  |  |  |  |  |
| 0 | Vvi-Vitvi12g02247\_t001 |  |  |  |  |  |  |  |  |  |  |  |  |
| 0 | Vvi-Vitvi12g02248\_t001 |  |  |  |  |  |  |  |  |  |  |  |  |
| 0 | Vvi-Vitvi12g00163\_t001 |  |  |  |  |  |  |  |  |  |  |  |  |
| 0 | Vvi-Vitvi12g00164\_t001 |  |  |  |  |  |  |  |  |  |  |  |  |
| 0 | Vvi-Vitvi12g00165\_t002 |  |  |  |  |  |  |  |  |  |  |  |  |
| 0 | Vvi-Vitvi12g00167\_t001 |  |  |  |  |  |  |  |  |  |  |  |  |
| 0 | Vvi-Vitvi12g00168\_t001 |  |  |  |  |  |  |  |  |  |  |  |  |
| 0 | Vvi-Vitvi12g00170\_t001 |  |  |  |  |  |  |  |  |  |  |  |  |
| 0 | Vvi-Vitvi12g04047\_t001 |  |  |  |  |  |  |  |  |  |  |  |  |
| 1 | Vvi-Vitvi12g00175\_t001 |  | Atr-ERN13866 |  |  |  |  |  |  |  |  |  |  |
| 1 | Vvi-Vitvi12g00177\_t001 |  | \| \| |  |  |  |  |  |  |  |  |  |  |
| 1 | Vvi-Vitvi12g04048\_t001 |  | \| \| |  |  |  |  |  |  |  |  |  |  |
| 1 | Vvi-Vitvi12g02249\_t001 |  | \| \| |  |  |  |  |  |  |  |  |  |  |
| 1 | Vvi-Vitvi12g00178\_t001 |  | \| \| |  |  |  |  |  |  |  |  |  |  |
| 1 | Vvi-Vitvi12g00179\_t001 |  | Atr-ERN13868 |  |  |  |  |  |  |  |  |  |  |
| 1 | Vvi-Vitvi12g00181\_t002 |  | Atr-ERN13872 |  |  |  |  |  |  |  |  |  |  |
| 1 | Vvi-Vitvi12g02250\_t001 |  | Atr-ERN13876 |  |  |  |  |  |  |  |  |  |  |
| 1 | Vvi-Vitvi12g00182\_t001 |  | \| \| |  |  |  |  |  |  |  |  |  |  |
| 1 | Vvi-Vitvi12g04049\_t001 |  | \| \| |  |  |  |  |  |  |  |  |  |  |
| 1 | Vvi-Vitvi12g00183\_t001 |  | \| \| |  |  |  |  |  |  |  |  |  |  |
| 1 | Vvi-Vitvi12g00184\_t001 |  | Atr-ERN13887 |  |  |  |  |  |  |  |  |  |  |
| 1 | Vvi-Vitvi12g00185\_t001 |  | Atr-ERN13889 |  |  |  |  |  |  |  |  |  |  |
| 1 | Vvi-Vitvi12g00186\_t001 |  | \| \| |  |  |  |  |  |  |  |  |  |  |
| 1 | Vvi-Vitvi12g02251\_t001 |  | \| \| |  |  |  |  |  |  |  |  |  |  |
| 1 | Vvi-Vitvi12g00187\_t001 |  | \| \| |  |  |  |  |  |  |  |  |  |  |
| 1 | Vvi-Vitvi12g00188\_t001 |  | \| \| |  |  |  |  |  |  |  |  |  |  |
| 1 | Vvi-Vitvi12g00189\_t001 |  | \| \| |  |  |  |  |  |  |  |  |  |  |
| 1 | Vvi-Vitvi12g00190\_t001 |  | \| \| |  |  |  |  |  |  |  |  |  |  |
| 1 | Vvi-Vitvi12g02252\_t001 |  | \| \| |  |  |  |  |  |  |  |  |  |  |
| 1 | Vvi-Vitvi12g02253\_t001 |  | Atr-ERN13894 |  |  |  |  |  |  |  |  |  |  |
| 1 | Vvi-Vitvi12g00192\_t001 |  | \| \| |  |  |  |  |  |  |  |  |  |  |
| 1 | Vvi-Vitvi12g00193\_t001 |  | Atr-ERN13895 |  |  |  |  |  |  |  |  |  |  |
| 1 | Vvi-Vitvi12g02254\_t001 |  | \| \| |  |  |  |  |  |  |  |  |  |  |
| 1 | Vvi-Vitvi12g02255\_t001 |  | \| \| |  |  |  |  |  |  |  |  |  |  |
| 1 | Vvi-Vitvi12g02256\_t001 |  | \| \| |  |  |  |  |  |  |  |  |  |  |
| 1 | Vvi-Vitvi12g04050\_t001 |  | \| \| |  |  |  |  |  |  |  |  |  |  |
| 1 | Vvi-Vitvi12g02257\_t001 |  | \| \| |  |  |  |  |  |  |  |  |  |  |
| 1 | Vvi-Vitvi12g00196\_t001 |  | \| \| |  |  |  |  |  |  |  |  |  |  |
| 1 | Vvi-Vitvi12g00197\_t001 |  | \| \| |  |  |  |  |  |  |  |  |  |  |
| 1 | Vvi-Vitvi12g00198\_t001 |  | Atr-ERN13903 |  |  |  |  |  |  |  |  |  |  |
| 1 | Vvi-Vitvi12g00200\_t001 |  | Atr-ERN13912 |  |  |  |  |  |  |  |  |  |  |
| 1 | Vvi-Vitvi12g00201\_t001 |  | Atr-ERN13913 |  |  |  |  |  |  |  |  |  |  |
| 1 | Vvi-Vitvi12g00202\_t001 |  | \| \| |  |  |  |  |  |  |  |  |  |  |
| 1 | Vvi-Vitvi12g04051\_t001 |  | \| \| |  |  |  |  |  |  |  |  |  |  |
| 1 | Vvi-Vitvi12g04052\_t001 |  | \| \| |  |  |  |  |  |  |  |  |  |  |
| 1 | Vvi-Vitvi12g00204\_t001 |  | Atr-ERN13915 |  |  |  |  |  |  |  |  |  |  |
| 1 | Vvi-Vitvi12g00205\_t001 |  | \| \| |  |  |  |  |  |  |  |  |  |  |
| 1 | Vvi-Vitvi12g00206\_t001 |  | Atr-ERN13924 |  |  |  |  |  |  |  |  |  |  |
| 1 | Vvi-Vitvi12g02260\_t001 |  | \| \| |  |  |  |  |  |  |  |  |  |  |
| 1 | Vvi-Vitvi12g00208\_t001 |  | \| \| |  |  |  |  |  |  |  |  |  |  |
| 1 | Vvi-Vitvi12g00209\_t001 |  | Atr-ERN13929 |  |  |  |  |  |  |  |  |  |  |
| 1 | Vvi-Vitvi12g00210\_t001 |  | \| \| |  |  |  |  |  |  |  |  |  |  |
| 1 | Vvi-Vitvi12g00211\_t001 |  | \| \| |  |  |  |  |  |  |  |  |  |  |
| 1 | Vvi-Vitvi12g00212\_t001 |  | Atr-ERN13943 |  |  |  |  |  |  |  |  |  |  |
| 1 | Vvi-Vitvi12g00213\_t001 |  | \| \| |  |  |  |  |  |  |  |  |  |  |
| 1 | Vvi-Vitvi12g02262\_t001 |  | Atr-ERN13950 |  |  |  |  |  |  |  |  |  |  |
| 1 | Vvi-Vitvi12g00214\_t001 |  | Atr-ERN13951 |  |  |  |  |  |  |  |  |  |  |
| 1 | Vvi-Vitvi12g00215\_t003 |  | Atr-ERN13953 |  |  |  |  |  |  |  |  |  |  |
| 1 | Vvi-Vitvi12g04053\_t001 |  | \| \| |  |  |  |  |  |  |  |  |  |  |
| 1 | Vvi-Vitvi12g00217\_t001 |  | Atr-ERN13966 |  |  |  |  |  |  |  |  |  |  |
| 1 | Vvi-Vitvi12g04054\_t001 |  | \| \| |  |  |  |  |  |  |  |  |  |  |
| 1 | Vvi-Vitvi12g04055\_t001 |  | \| \| |  |  |  |  |  |  |  |  |  |  |
| 1 | Vvi-Vitvi12g04056\_t001 |  | \| \| |  |  |  |  |  |  |  |  |  |  |
| 1 | Vvi-Vitvi12g00219\_t001 |  | Atr-ERN13972 |  |  |  |  |  |  |  |  |  |  |
| 1 | Vvi-Vitvi12g00220\_t001 |  | Atr-ERN13976 |  |  |  |  |  |  |  |  |  |  |
| 1 | Vvi-Vitvi12g00221\_t001 |  | Atr-ERN13978 |  |  |  |  |  |  |  |  |  |  |
| 1 | Vvi-Vitvi12g04057\_t001 |  | Atr-ERN13980 |  |  |  |  |  |  |  |  |  |  |
| 1 | Vvi-Vitvi12g02263\_t001 |  | \| \| |  |  |  |  |  |  |  |  |  |  |
| 1 | Vvi-Vitvi12g02264\_t001 |  | \| \| |  |  |  |  |  |  |  |  |  |  |
| 1 | Vvi-Vitvi12g04058\_t001 |  | \| \| |  |  |  |  |  |  |  |  |  |  |
| 1 | Vvi-Vitvi12g02265\_t001 |  | \| \| |  |  |  |  |  |  |  |  |  |  |
| 1 | Vvi-Vitvi12g02266\_t001 |  | \| \| |  |  |  |  |  |  |  |  |  |  |
| 1 | Vvi-Vitvi12g00224\_t001 |  | \| \| |  |  |  |  |  |  |  |  |  |  |
| 1 | Vvi-Vitvi12g00225\_t002 |  | Atr-ERN13982 |  |  |  |  |  |  |  |  |  |  |
| 1 | Vvi-Vitvi12g00226\_t001 |  | Atr-ERN13983 |  |  |  |  |  |  |  |  |  |  |
| 1 | Vvi-Vitvi12g00227\_t001 |  | \| \| |  |  |  |  |  |  |  |  |  |  |
| 1 | Vvi-Vitvi12g00228\_t001 |  | \| \| |  |  |  |  |  |  |  |  |  |  |
| 1 | Vvi-Vitvi12g00229\_t001 |  | Atr-ERN14004 |  |  |  |  |  |  |  |  |  |  |
| 1 | Vvi-Vitvi12g00230\_t001 |  | \| \| |  |  |  |  |  |  |  |  |  |  |
| 1 | Vvi-Vitvi12g04059\_t001 |  | \| \| |  |  |  |  |  |  |  |  |  |  |
| 1 | Vvi-Vitvi12g00231\_t001 |  | Atr-ERN14007 |  |  |  |  |  |  |  |  |  |  |
| 1 | Vvi-Vitvi12g00232\_t001 |  | \| \| |  |  |  |  |  |  |  |  |  |  |
| 1 | Vvi-Vitvi12g00233\_t001 |  | Atr-ERN14011 |  |  |  |  |  |  |  |  |  |  |
| 1 | Vvi-Vitvi12g00234\_t001 |  | Atr-ERN14015 |  |  |  |  |  |  |  |  |  |  |
| 1 | Vvi-Vitvi12g00235\_t001 |  | Atr-ERN14021 |  |  |  |  |  |  |  |  |  |  |
| 1 | Vvi-Vitvi12g00236\_t001 |  | Atr-ERN14024 |  |  |  |  |  |  |  |  |  |  |
| 1 | Vvi-Vitvi12g04060\_t001 |  | \| \| |  |  |  |  |  |  |  |  |  |  |
| 1 | Vvi-Vitvi12g00239\_t001 |  | \| \| |  |  |  |  |  |  |  |  |  |  |
| 1 | Vvi-Vitvi12g02269\_t001 |  | \| \| |  |  |  |  |  |  |  |  |  |  |
| 1 | Vvi-Vitvi12g00240\_t001 |  | \| \| |  |  |  |  |  |  |  |  |  |  |
| 1 | Vvi-Vitvi12g02272\_t001 |  | \| \| |  |  |  |  |  |  |  |  |  |  |
| 1 | Vvi-Vitvi12g00242\_t001 |  | \| \| |  |  |  |  |  |  |  |  |  |  |
| 1 | Vvi-Vitvi12g04061\_t001 |  | \| \| |  |  |  |  |  |  |  |  |  |  |
| 1 | Vvi-Vitvi12g02273\_t001 |  | \| \| |  |  |  |  |  |  |  |  |  |  |
| 1 | Vvi-Vitvi12g02274\_t001 |  | \| \| |  |  |  |  |  |  |  |  |  |  |
| 1 | Vvi-Vitvi12g02275\_t001 |  | \| \| |  |  |  |  |  |  |  |  |  |  |
| 1 | Vvi-Vitvi12g02277\_t001 |  | \| \| |  |  |  |  |  |  |  |  |  |  |
| 1 | Vvi-Vitvi12g02280\_t001 |  | \| \| |  |  |  |  |  |  |  |  |  |  |
| 1 | Vvi-Vitvi12g02281\_t001 |  | \| \| |  |  |  |  |  |  |  |  |  |  |
| 1 | Vvi-Vitvi12g02282\_t001 |  | \| \| |  |  |  |  |  |  |  |  |  |  |
| 1 | Vvi-Vitvi12g02283\_t001 |  | \| \| |  |  |  |  |  |  |  |  |  |  |
| 1 | Vvi-Vitvi12g02289\_t001 |  | \| \| |  |  |  |  |  |  |  |  |  |  |
| 1 | Vvi-Vitvi12g02293\_t002 |  | \| \| |  |  |  |  |  |  |  |  |  |  |
| 1 | Vvi-Vitvi12g04062\_t001 |  | \| \| |  |  |  |  |  |  |  |  |  |  |
| 1 | Vvi-Vitvi12g02292\_t001 |  | \| \| |  |  |  |  |  |  |  |  |  |  |
| 1 | Vvi-Vitvi12g00246\_t001 |  | \| \| |  |  |  |  |  |  |  |  |  |  |
| 1 | Vvi-Vitvi12g00248\_t001 |  | \| \| |  |  |  |  |  |  |  |  |  |  |
| 1 | Vvi-Vitvi12g00251\_t001 |  | Atr-ERN14036 |  |  |  |  |  |  |  |  |  |  |
| 1 | Vvi-Vitvi12g02295\_t001 |  | Atr-ERN14037 |  |  |  |  |  |  |  |  |  |  |
| 1 | Vvi-Vitvi12g00252\_t001 |  | \| \| |  |  |  |  |  |  |  |  |  |  |
| 1 | Vvi-Vitvi12g02296\_t001 |  | Atr-ERN14040 |  |  |  |  |  |  |  |  |  |  |
| 1 | Vvi-Vitvi12g04063\_t001 |  | \| \| |  |  |  |  |  |  |  |  |  |  |
| 1 | Vvi-Vitvi12g00253\_t001 |  | \| \| |  |  |  |  |  |  |  |  |  |  |
| 1 | Vvi-Vitvi12g00255\_t001 |  | Atr-ERN14042 |  |  |  |  |  |  |  |  |  |  |
| 1 | Vvi-Vitvi12g00256\_t001 |  | Atr-ERN14047 |  |  |  |  |  |  |  |  |  |  |
| 1 | Vvi-Vitvi12g00257\_t001 |  | \| \| |  |  |  |  |  |  |  |  |  |  |
| 1 | Vvi-Vitvi12g00258\_t003 |  | \| \| |  |  |  |  |  |  |  |  |  |  |
| 1 | Vvi-Vitvi12g00260\_t001 |  | \| \| |  |  |  |  |  |  |  |  |  |  |
| 1 | Vvi-Vitvi12g00261\_t001 |  | \| \| |  |  |  |  |  |  |  |  |  |  |
| 1 | Vvi-Vitvi12g00262\_t001 |  | \| \| |  |  |  |  |  |  |  |  |  |  |
| 1 | Vvi-Vitvi12g00263\_t001 |  | \| \| |  |  |  |  |  |  |  |  |  |  |
| 1 | Vvi-Vitvi12g00264\_t001 |  | \| \| |  |  |  |  |  |  |  |  |  |  |
| 1 | Vvi-Vitvi12g02297\_t001 |  | \| \| |  |  |  |  |  |  |  |  |  |  |
| 1 | Vvi-Vitvi12g00267\_t004 |  | \| \| |  |  |  |  |  |  |  |  |  |  |
| 1 | Vvi-Vitvi12g00268\_t001 |  | \| \| |  |  |  |  |  |  |  |  |  |  |
| 1 | Vvi-Vitvi12g00269\_t001 |  | Atr-ERN14050 |  |  |  |  |  |  |  |  |  |  |
| 0 | Vvi-Vitvi12g00270\_t001 |  |  |  |  |  |  |  |  |  |  |  |  |
| 0 | Vvi-Vitvi12g04064\_t001 |  |  |  |  |  |  |  |  |  |  |  |  |
| 0 | Vvi-Vitvi12g04065\_t001 |  |  |  |  |  |  |  |  |  |  |  |  |
| 0 | Vvi-Vitvi12g02300\_t001 |  |  |  |  |  |  |  |  |  |  |  |  |
| 0 | Vvi-Vitvi12g00272\_t001 |  |  |  |  |  |  |  |  |  |  |  |  |
| 0 | Vvi-Vitvi12g00274\_t001 |  |  |  |  |  |  |  |  |  |  |  |  |
| 0 | Vvi-Vitvi12g00275\_t002 |  |  |  |  |  |  |  |  |  |  |  |  |
| 0 | Vvi-Vitvi12g00276\_t001 |  |  |  |  |  |  |  |  |  |  |  |  |
| 0 | Vvi-Vitvi12g00277\_t001 |  |  |  |  |  |  |  |  |  |  |  |  |
| 0 | Vvi-Vitvi12g00278\_t001 |  |  |  |  |  |  |  |  |  |  |  |  |
| 0 | Vvi-Vitvi12g00279\_t001 |  |  |  |  |  |  |  |  |  |  |  |  |
| 0 | Vvi-Vitvi12g00280\_t001 |  |  |  |  |  |  |  |  |  |  |  |  |
| 0 | Vvi-Vitvi12g00281\_t001 |  |  |  |  |  |  |  |  |  |  |  |  |
| 0 | Vvi-Vitvi12g00283\_t001 |  |  |  |  |  |  |  |  |  |  |  |  |
| 0 | Vvi-Vitvi12g00284\_t001 |  |  |  |  |  |  |  |  |  |  |  |  |
| 0 | Vvi-Vitvi12g00285\_t001 |  |  |  |  |  |  |  |  |  |  |  |  |
| 0 | Vvi-Vitvi12g00286\_t001 |  |  |  |  |  |  |  |  |  |  |  |  |
| 0 | Vvi-Vitvi12g00287\_t001 |  |  |  |  |  |  |  |  |  |  |  |  |
| 0 | Vvi-Vitvi12g00288\_t001 |  |  |  |  |  |  |  |  |  |  |  |  |
| 0 | Vvi-Vitvi12g00289\_t001 |  |  |  |  |  |  |  |  |  |  |  |  |
| 0 | Vvi-Vitvi12g00290\_t001 |  |  |  |  |  |  |  |  |  |  |  |  |
| 1 | Vvi-Vitvi12g04066\_t001 |  | Atr-ERN20482 |  |  |  |  |  |  |  |  |  |  |
| 1 | Vvi-Vitvi12g00292\_t001 |  | \| \| |  |  |  |  |  |  |  |  |  |  |
| 1 | Vvi-Vitvi12g00294\_t001 |  | Atr-ERN20489 |  |  |  |  |  |  |  |  |  |  |
| 1 | Vvi-Vitvi12g00297\_t001 |  | \| \| |  |  |  |  |  |  |  |  |  |  |
| 1 | Vvi-Vitvi12g04067\_t001 |  | \| \| |  |  |  |  |  |  |  |  |  |  |
| 1 | Vvi-Vitvi12g02305\_t001 |  | \| \| |  |  |  |  |  |  |  |  |  |  |
| 1 | Vvi-Vitvi12g02306\_t001 |  | \| \| |  |  |  |  |  |  |  |  |  |  |
| 1 | Vvi-Vitvi12g04068\_t001 |  | \| \| |  |  |  |  |  |  |  |  |  |  |
| 1 | Vvi-Vitvi12g02307\_t001 |  | \| \| |  |  |  |  |  |  |  |  |  |  |
| 1 | Vvi-Vitvi12g00298\_t001 |  | Atr-ERN20493 |  |  |  |  |  |  |  |  |  |  |
| 1 | Vvi-Vitvi12g00299\_t002 |  | Atr-ERN20494 |  |  |  |  |  |  |  |  |  |  |
| 1 | Vvi-Vitvi12g00300\_t001 |  | Atr-ERN20496 |  |  |  |  |  |  |  |  |  |  |
| 1 | Vvi-Vitvi12g00301\_t001 |  | Atr-ERN20499 |  |  |  |  |  |  |  |  |  |  |
| 1 | Vvi-Vitvi12g00302\_t001 |  | \| \| |  |  |  |  |  |  |  |  |  |  |
| 1 | Vvi-Vitvi12g02308\_t001 |  | \| \| |  |  |  |  |  |  |  |  |  |  |
| 1 | Vvi-Vitvi12g00303\_t004 |  | Atr-ERN20506 |  |  |  |  |  |  |  |  |  |  |
| 1 | Vvi-Vitvi12g02309\_t001 |  | \| \| |  |  |  |  |  |  |  |  |  |  |
| 1 | Vvi-Vitvi12g00304\_t001 |  | Atr-ERN20510 |  |  |  |  |  |  |  |  |  |  |
| 1 | Vvi-Vitvi12g00305\_t001 |  | Atr-ERN20513 |  |  |  |  |  |  |  |  |  |  |
| 1 | Vvi-Vitvi12g00306\_t001 |  | Atr-ERN20514 |  |  |  |  |  |  |  |  |  |  |
| 0 | Vvi-Vitvi12g04069\_t001 |  |  |  |  |  |  |  |  |  |  |  |  |
| 0 | Vvi-Vitvi12g00309\_t001 |  |  |  |  |  |  |  |  |  |  |  |  |
| 0 | Vvi-Vitvi12g00310\_t001 |  |  |  |  |  |  |  |  |  |  |  |  |
| 0 | Vvi-Vitvi12g00312\_t001 |  |  |  |  |  |  |  |  |  |  |  |  |
| 0 | Vvi-Vitvi12g00313\_t001 |  |  |  |  |  |  |  |  |  |  |  |  |
| 0 | Vvi-Vitvi12g00314\_t001 |  |  |  |  |  |  |  |  |  |  |  |  |
| 0 | Vvi-Vitvi12g00315\_t001 |  |  |  |  |  |  |  |  |  |  |  |  |
| 0 | Vvi-Vitvi12g00316\_t001 |  |  |  |  |  |  |  |  |  |  |  |  |
| 0 | Vvi-Vitvi12g00317\_t001 |  |  |  |  |  |  |  |  |  |  |  |  |
| 0 | Vvi-Vitvi12g00319\_t001 |  |  |  |  |  |  |  |  |  |  |  |  |
| 0 | Vvi-Vitvi12g04070\_t001 |  |  |  |  |  |  |  |  |  |  |  |  |
| 0 | Vvi-Vitvi12g00320\_t001 |  |  |  |  |  |  |  |  |  |  |  |  |
| 0 | Vvi-Vitvi12g04071\_t001 |  |  |  |  |  |  |  |  |  |  |  |  |
| 0 | Vvi-Vitvi12g00322\_t001 |  |  |  |  |  |  |  |  |  |  |  |  |
| 0 | Vvi-Vitvi12g04072\_t001 |  |  |  |  |  |  |  |  |  |  |  |  |
| 0 | Vvi-Vitvi12g04073\_t001 |  |  |  |  |  |  |  |  |  |  |  |  |
| 0 | Vvi-Vitvi12g00324\_t001 |  |  |  |  |  |  |  |  |  |  |  |  |
| 0 | Vvi-Vitvi12g04074\_t001 |  |  |  |  |  |  |  |  |  |  |  |  |
| 0 | Vvi-Vitvi12g00325\_t001 |  |  |  |  |  |  |  |  |  |  |  |  |
| 0 | Vvi-Vitvi12g00326\_t001 |  |  |  |  |  |  |  |  |  |  |  |  |
| 0 | Vvi-Vitvi12g00327\_t001 |  |  |  |  |  |  |  |  |  |  |  |  |
| 0 | Vvi-Vitvi12g00328\_t001 |  |  |  |  |  |  |  |  |  |  |  |  |
| 0 | Vvi-Vitvi12g00329\_t001 |  |  |  |  |  |  |  |  |  |  |  |  |
| 0 | Vvi-Vitvi12g04075\_t001 |  |  |  |  |  |  |  |  |  |  |  |  |
| 0 | Vvi-Vitvi12g04076\_t001 |  |  |  |  |  |  |  |  |  |  |  |  |
| 0 | Vvi-Vitvi12g02313\_t001 |  |  |  |  |  |  |  |  |  |  |  |  |
| 0 | Vvi-Vitvi12g04077\_t001 |  |  |  |  |  |  |  |  |  |  |  |  |
| 0 | Vvi-Vitvi12g04078\_t001 |  |  |  |  |  |  |  |  |  |  |  |  |
| 0 | Vvi-Vitvi12g04079\_t001 |  |  |  |  |  |  |  |  |  |  |  |  |
| 0 | Vvi-Vitvi12g04080\_t001 |  |  |  |  |  |  |  |  |  |  |  |  |
| 0 | Vvi-Vitvi12g04081\_t001 |  |  |  |  |  |  |  |  |  |  |  |  |
| 0 | Vvi-Vitvi12g02316\_t001 |  |  |  |  |  |  |  |  |  |  |  |  |
| 0 | Vvi-Vitvi12g04082\_t001 |  |  |  |  |  |  |  |  |  |  |  |  |
| 0 | Vvi-Vitvi12g04083\_t001 |  |  |  |  |  |  |  |  |  |  |  |  |
| 0 | Vvi-Vitvi12g04084\_t001 |  |  |  |  |  |  |  |  |  |  |  |  |
| 0 | Vvi-Vitvi12g04085\_t001 |  |  |  |  |  |  |  |  |  |  |  |  |
| 0 | Vvi-Vitvi12g02318\_t001 |  |  |  |  |  |  |  |  |  |  |  |  |
| 0 | Vvi-Vitvi12g00334\_t001 |  |  |  |  |  |  |  |  |  |  |  |  |
| 0 | Vvi-Vitvi12g00335\_t001 |  |  |  |  |  |  |  |  |  |  |  |  |
| 0 | Vvi-Vitvi12g00336\_t001 |  |  |  |  |  |  |  |  |  |  |  |  |
| 0 | Vvi-Vitvi12g04086\_t001 |  |  |  |  |  |  |  |  |  |  |  |  |
| 0 | Vvi-Vitvi12g04087\_t001 |  |  |  |  |  |  |  |  |  |  |  |  |
| 0 | Vvi-Vitvi12g04088\_t001 |  |  |  |  |  |  |  |  |  |  |  |  |
| 0 | Vvi-Vitvi12g02324\_t001 |  |  |  |  |  |  |  |  |  |  |  |  |
| 0 | Vvi-Vitvi12g04089\_t001 |  |  |  |  |  |  |  |  |  |  |  |  |
| 0 | Vvi-Vitvi12g04090\_t001 |  |  |  |  |  |  |  |  |  |  |  |  |
| 0 | Vvi-Vitvi12g04091\_t001 |  |  |  |  |  |  |  |  |  |  |  |  |
| 0 | Vvi-Vitvi12g04092\_t001 |  |  |  |  |  |  |  |  |  |  |  |  |
| 0 | Vvi-Vitvi12g02326\_t001 |  |  |  |  |  |  |  |  |  |  |  |  |
| 0 | Vvi-Vitvi12g04093\_t001 |  |  |  |  |  |  |  |  |  |  |  |  |
| 0 | Vvi-Vitvi12g04094\_t001 |  |  |  |  |  |  |  |  |  |  |  |  |
| 0 | Vvi-Vitvi12g02330\_t001 |  |  |  |  |  |  |  |  |  |  |  |  |
| 0 | Vvi-Vitvi12g02331\_t001 |  |  |  |  |  |  |  |  |  |  |  |  |
| 0 | Vvi-Vitvi12g04095\_t001 |  |  |  |  |  |  |  |  |  |  |  |  |
| 0 | Vvi-Vitvi12g00339\_t001 |  |  |  |  |  |  |  |  |  |  |  |  |
| 1 | Vvi-Vitvi12g00340\_t001 |  | Atr-ERM95199 |  |  |  |  |  |  |  |  |  |  |
| 1 | Vvi-Vitvi12g00341\_t001 |  | Atr-ERM95204 |  |  |  |  |  |  |  |  |  |  |
| 1 | Vvi-Vitvi12g00342\_t001 |  | Atr-ERM95207 |  |  |  |  |  |  |  |  |  |  |
| 1 | Vvi-Vitvi12g00343\_t001 |  | Atr-ERM95223 |  |  |  |  |  |  |  |  |  |  |
| 1 | Vvi-Vitvi12g02332\_t001 |  | Atr-ERM95227 |  |  |  |  |  |  |  |  |  |  |
| 1 | Vvi-Vitvi12g02334\_t002 |  | \| \| |  |  |  |  |  |  |  |  |  |  |
| 1 | Vvi-Vitvi12g04096\_t001 |  | \| \| |  |  |  |  |  |  |  |  |  |  |
| 1 | Vvi-Vitvi12g04097\_t001 |  | \| \| |  |  |  |  |  |  |  |  |  |  |
| 1 | Vvi-Vitvi12g00344\_t001 |  | Atr-ERM95231 |  |  |  |  |  |  |  |  |  |  |
| 1 | Vvi-Vitvi12g00345\_t001 |  | \| \| |  |  |  |  |  |  |  |  |  |  |
| 1 | Vvi-Vitvi12g00346\_t001 |  | \| \| |  |  |  |  |  |  |  |  |  |  |
| 1 | Vvi-Vitvi12g04098\_t001 |  | \| \| |  |  |  |  |  |  |  |  |  |  |
| 1 | Vvi-Vitvi12g00348\_t001 |  | Atr-ERM95240 |  |  |  |  |  |  |  |  |  |  |
| 0 | Vvi-Vitvi12g00349\_t001 |  |  |  |  |  |  |  |  |  |  |  |  |
| 0 | Vvi-Vitvi12g04099\_t001 |  |  |  |  |  |  |  |  |  |  |  |  |
| 0 | Vvi-Vitvi12g04100\_t001 |  |  |  |  |  |  |  |  |  |  |  |  |
| 0 | Vvi-Vitvi12g02337\_t001 |  |  |  |  |  |  |  |  |  |  |  |  |
| 0 | Vvi-Vitvi12g02340\_t001 |  |  |  |  |  |  |  |  |  |  |  |  |
| 0 | Vvi-Vitvi12g04101\_t001 |  |  |  |  |  |  |  |  |  |  |  |  |
| 0 | Vvi-Vitvi12g04102\_t001 |  |  |  |  |  |  |  |  |  |  |  |  |
| 0 | Vvi-Vitvi12g00351\_t001 |  |  |  |  |  |  |  |  |  |  |  |  |
| 0 | Vvi-Vitvi12g04103\_t001 |  |  |  |  |  |  |  |  |  |  |  |  |
| 0 | Vvi-Vitvi12g02342\_t001 |  |  |  |  |  |  |  |  |  |  |  |  |
| 0 | Vvi-Vitvi12g02344\_t001 |  |  |  |  |  |  |  |  |  |  |  |  |
| 0 | Vvi-Vitvi12g02345\_t001 |  |  |  |  |  |  |  |  |  |  |  |  |
| 0 | Vvi-Vitvi12g02348\_t001 |  |  |  |  |  |  |  |  |  |  |  |  |
| 0 | Vvi-Vitvi12g04104\_t001 |  |  |  |  |  |  |  |  |  |  |  |  |
| 0 | Vvi-Vitvi12g02350\_t001 |  |  |  |  |  |  |  |  |  |  |  |  |
| 0 | Vvi-Vitvi12g02351\_t001 |  |  |  |  |  |  |  |  |  |  |  |  |
| 0 | Vvi-Vitvi12g04105\_t001 |  |  |  |  |  |  |  |  |  |  |  |  |
| 0 | Vvi-Vitvi12g04106\_t001 |  |  |  |  |  |  |  |  |  |  |  |  |
| 0 | Vvi-Vitvi12g04107\_t001 |  |  |  |  |  |  |  |  |  |  |  |  |
| 0 | Vvi-Vitvi12g04108\_t001 |  |  |  |  |  |  |  |  |  |  |  |  |
| 0 | Vvi-Vitvi12g02353\_t001 |  |  |  |  |  |  |  |  |  |  |  |  |
| 0 | Vvi-Vitvi12g00353\_t003 |  |  |  |  |  |  |  |  |  |  |  |  |
| 0 | Vvi-Vitvi12g04109\_t001 |  |  |  |  |  |  |  |  |  |  |  |  |
| 0 | Vvi-Vitvi12g04110\_t001 |  |  |  |  |  |  |  |  |  |  |  |  |
| 0 | Vvi-Vitvi12g04111\_t001 |  |  |  |  |  |  |  |  |  |  |  |  |
| 0 | Vvi-Vitvi12g04112\_t001 |  |  |  |  |  |  |  |  |  |  |  |  |
| 0 | Vvi-Vitvi12g00357\_t001 |  |  |  |  |  |  |  |  |  |  |  |  |
| 0 | Vvi-Vitvi12g00358\_t001 |  |  |  |  |  |  |  |  |  |  |  |  |
| 0 | Vvi-Vitvi12g00360\_t001 |  |  |  |  |  |  |  |  |  |  |  |  |
| 0 | Vvi-Vitvi12g00361\_t001 |  |  |  |  |  |  |  |  |  |  |  |  |
| 0 | Vvi-Vitvi12g00362\_t003 |  |  |  |  |  |  |  |  |  |  |  |  |
| 0 | Vvi-Vitvi12g00363\_t001 |  |  |  |  |  |  |  |  |  |  |  |  |
| 0 | Vvi-Vitvi12g02356\_t001 |  |  |  |  |  |  |  |  |  |  |  |  |
| 0 | Vvi-Vitvi12g00364\_t001 |  |  |  |  |  |  |  |  |  |  |  |  |
| 0 | Vvi-Vitvi12g00365\_t002 |  |  |  |  |  |  |  |  |  |  |  |  |
| 0 | Vvi-Vitvi12g00367\_t001 |  |  |  |  |  |  |  |  |  |  |  |  |
| 1 | Vvi-Vitvi12g00368\_t001 |  | Atr-ERN17362 |  |  |  |  |  |  |  |  |  |  |
| 1 | Vvi-Vitvi12g00369\_t001 |  | \| \| |  |  |  |  |  |  |  |  |  |  |
| 1 | Vvi-Vitvi12g04113\_t001 |  | \| \| |  |  |  |  |  |  |  |  |  |  |
| 1 | Vvi-Vitvi12g00370\_t002 |  | \| \| |  |  |  |  |  |  |  |  |  |  |
| 1 | Vvi-Vitvi12g04114\_t001 |  | \| \| |  |  |  |  |  |  |  |  |  |  |
| 1 | Vvi-Vitvi12g00373\_t001 |  | \| \| |  |  |  |  |  |  |  |  |  |  |
| 1 | Vvi-Vitvi12g04115\_t001 |  | \| \| |  |  |  |  |  |  |  |  |  |  |
| 1 | Vvi-Vitvi12g04116\_t001 |  | \| \| |  |  |  |  |  |  |  |  |  |  |
| 1 | Vvi-Vitvi12g00376\_t001 |  | Atr-ERN17373 |  |  |  |  |  |  |  |  |  |  |
| 1 | Vvi-Vitvi12g00377\_t001 |  | Atr-ERN17385 |  |  |  |  |  |  |  |  |  |  |
| 1 | Vvi-Vitvi12g00379\_t001 |  | \| \| |  |  |  |  |  |  |  |  |  |  |
| 1 | Vvi-Vitvi12g00380\_t001 |  | Atr-ERN17391 |  |  |  |  |  |  |  |  |  |  |
| 1 | Vvi-Vitvi12g00381\_t001 |  | Atr-ERN17395 |  |  |  |  |  |  |  |  |  |  |
| 1 | Vvi-Vitvi12g00382\_t001 |  | Atr-ERN17406 |  |  |  |  |  |  |  |  |  |  |
| 0 | Vvi-Vitvi12g04117\_t001 |  |  |  |  |  |  |  |  |  |  |  |  |
| 0 | Vvi-Vitvi12g04118\_t001 |  |  |  |  |  |  |  |  |  |  |  |  |
| 0 | Vvi-Vitvi12g04119\_t001 |  |  |  |  |  |  |  |  |  |  |  |  |
| 0 | Vvi-Vitvi12g00383\_t001 |  |  |  |  |  |  |  |  |  |  |  |  |
| 0 | Vvi-Vitvi12g00384\_t001 |  |  |  |  |  |  |  |  |  |  |  |  |
| 0 | Vvi-Vitvi12g00385\_t001 |  |  |  |  |  |  |  |  |  |  |  |  |
| 0 | Vvi-Vitvi12g02362\_t001 |  |  |  |  |  |  |  |  |  |  |  |  |
| 0 | Vvi-Vitvi12g02363\_t001 |  |  |  |  |  |  |  |  |  |  |  |  |
| 0 | Vvi-Vitvi12g02364\_t001 |  |  |  |  |  |  |  |  |  |  |  |  |
| 0 | Vvi-Vitvi12g02365\_t001 |  |  |  |  |  |  |  |  |  |  |  |  |
| 0 | Vvi-Vitvi12g04120\_t001 |  |  |  |  |  |  |  |  |  |  |  |  |
| 0 | Vvi-Vitvi12g02366\_t001 |  |  |  |  |  |  |  |  |  |  |  |  |
| 0 | Vvi-Vitvi12g02367\_t001 |  |  |  |  |  |  |  |  |  |  |  |  |
| 0 | Vvi-Vitvi12g00386\_t001 |  |  |  |  |  |  |  |  |  |  |  |  |
| 0 | Vvi-Vitvi12g00387\_t001 |  |  |  |  |  |  |  |  |  |  |  |  |
| 0 | Vvi-Vitvi12g00388\_t001 |  |  |  |  |  |  |  |  |  |  |  |  |
| 0 | Vvi-Vitvi12g04121\_t001 |  |  |  |  |  |  |  |  |  |  |  |  |
| 0 | Vvi-Vitvi12g02368\_t001 |  |  |  |  |  |  |  |  |  |  |  |  |
| 0 | Vvi-Vitvi12g04122\_t001 |  |  |  |  |  |  |  |  |  |  |  |  |
| 0 | Vvi-Vitvi12g00389\_t001 |  |  |  |  |  |  |  |  |  |  |  |  |
| 0 | Vvi-Vitvi12g02369\_t001 |  |  |  |  |  |  |  |  |  |  |  |  |
| 0 | Vvi-Vitvi12g00390\_t001 |  |  |  |  |  |  |  |  |  |  |  |  |
| 0 | Vvi-Vitvi12g00391\_t001 |  |  |  |  |  |  |  |  |  |  |  |  |
| 0 | Vvi-Vitvi12g04123\_t001 |  |  |  |  |  |  |  |  |  |  |  |  |
| 0 | Vvi-Vitvi12g04124\_t001 |  |  |  |  |  |  |  |  |  |  |  |  |
| 0 | Vvi-Vitvi12g00395\_t001 |  |  |  |  |  |  |  |  |  |  |  |  |
| 0 | Vvi-Vitvi12g02371\_t001 |  |  |  |  |  |  |  |  |  |  |  |  |
| 0 | Vvi-Vitvi12g04125\_t001 |  |  |  |  |  |  |  |  |  |  |  |  |
| 0 | Vvi-Vitvi12g00396\_t001 |  |  |  |  |  |  |  |  |  |  |  |  |
| 0 | Vvi-Vitvi12g04126\_t001 |  |  |  |  |  |  |  |  |  |  |  |  |
| 0 | Vvi-Vitvi12g04127\_t001 |  |  |  |  |  |  |  |  |  |  |  |  |
| 0 | Vvi-Vitvi12g02374\_t001 |  |  |  |  |  |  |  |  |  |  |  |  |
| 0 | Vvi-Vitvi12g04128\_t001 |  |  |  |  |  |  |  |  |  |  |  |  |
| 0 | Vvi-Vitvi12g04129\_t001 |  |  |  |  |  |  |  |  |  |  |  |  |
| 0 | Vvi-Vitvi12g04130\_t001 |  |  |  |  |  |  |  |  |  |  |  |  |
| 0 | Vvi-Vitvi12g00401\_t001 |  |  |  |  |  |  |  |  |  |  |  |  |
| 0 | Vvi-Vitvi12g00402\_t001 |  |  |  |  |  |  |  |  |  |  |  |  |
| 0 | Vvi-Vitvi12g00403\_t001 |  |  |  |  |  |  |  |  |  |  |  |  |
| 0 | Vvi-Vitvi12g00404\_t001 |  |  |  |  |  |  |  |  |  |  |  |  |
| 0 | Vvi-Vitvi12g02375\_t001 |  |  |  |  |  |  |  |  |  |  |  |  |
| 0 | Vvi-Vitvi12g00405\_t001 |  |  |  |  |  |  |  |  |  |  |  |  |
| 0 | Vvi-Vitvi12g00406\_t001 |  |  |  |  |  |  |  |  |  |  |  |  |
| 0 | Vvi-Vitvi12g00409\_t001 |  |  |  |  |  |  |  |  |  |  |  |  |
| 0 | Vvi-Vitvi12g00410\_t001 |  |  |  |  |  |  |  |  |  |  |  |  |
| 0 | Vvi-Vitvi12g00411\_t001 |  |  |  |  |  |  |  |  |  |  |  |  |
| 0 | Vvi-Vitvi12g02376\_t001 |  |  |  |  |  |  |  |  |  |  |  |  |
| 0 | Vvi-Vitvi12g00414\_t001 |  |  |  |  |  |  |  |  |  |  |  |  |
| 0 | Vvi-Vitvi12g00415\_t001 |  |  |  |  |  |  |  |  |  |  |  |  |
| 0 | Vvi-Vitvi12g04131\_t001 |  |  |  |  |  |  |  |  |  |  |  |  |
| 0 | Vvi-Vitvi12g04132\_t001 |  |  |  |  |  |  |  |  |  |  |  |  |
| 0 | Vvi-Vitvi12g00419\_t001 |  |  |  |  |  |  |  |  |  |  |  |  |
| 0 | Vvi-Vitvi12g00420\_t001 |  |  |  |  |  |  |  |  |  |  |  |  |
| 0 | Vvi-Vitvi12g00421\_t001 |  |  |  |  |  |  |  |  |  |  |  |  |
| 0 | Vvi-Vitvi12g00422\_t001 |  |  |  |  |  |  |  |  |  |  |  |  |
| 0 | Vvi-Vitvi12g00423\_t001 |  |  |  |  |  |  |  |  |  |  |  |  |
| 0 | Vvi-Vitvi12g00424\_t001 |  |  |  |  |  |  |  |  |  |  |  |  |
| 0 | Vvi-Vitvi12g00425\_t001 |  |  |  |  |  |  |  |  |  |  |  |  |
| 0 | Vvi-Vitvi12g00426\_t001 |  |  |  |  |  |  |  |  |  |  |  |  |
| 0 | Vvi-Vitvi12g00427\_t001 |  |  |  |  |  |  |  |  |  |  |  |  |
| 0 | Vvi-Vitvi12g00428\_t002 |  |  |  |  |  |  |  |  |  |  |  |  |
| 0 | Vvi-Vitvi12g00429\_t001 |  |  |  |  |  |  |  |  |  |  |  |  |
| 0 | Vvi-Vitvi12g00430\_t001 |  |  |  |  |  |  |  |  |  |  |  |  |
| 0 | Vvi-Vitvi12g00431\_t001 |  |  |  |  |  |  |  |  |  |  |  |  |
| 0 | Vvi-Vitvi12g00432\_t001 |  |  |  |  |  |  |  |  |  |  |  |  |
| 0 | Vvi-Vitvi12g00433\_t001 |  |  |  |  |  |  |  |  |  |  |  |  |
| 0 | Vvi-Vitvi12g00437\_t001 |  |  |  |  |  |  |  |  |  |  |  |  |
| 0 | Vvi-Vitvi12g04133\_t001 |  |  |  |  |  |  |  |  |  |  |  |  |
| 0 | Vvi-Vitvi12g00438\_t001 |  |  |  |  |  |  |  |  |  |  |  |  |
| 0 | Vvi-Vitvi12g00439\_t001 |  |  |  |  |  |  |  |  |  |  |  |  |
| 0 | Vvi-Vitvi12g00440\_t001 |  |  |  |  |  |  |  |  |  |  |  |  |
| 0 | Vvi-Vitvi12g00441\_t001 |  |  |  |  |  |  |  |  |  |  |  |  |
| 0 | Vvi-Vitvi12g04134\_t001 |  |  |  |  |  |  |  |  |  |  |  |  |
| 0 | Vvi-Vitvi12g00443\_t001 |  |  |  |  |  |  |  |  |  |  |  |  |
| 0 | Vvi-Vitvi12g02377\_t001 |  |  |  |  |  |  |  |  |  |  |  |  |
| 0 | Vvi-Vitvi12g02378\_t001 |  |  |  |  |  |  |  |  |  |  |  |  |
| 0 | Vvi-Vitvi12g00444\_t001 |  |  |  |  |  |  |  |  |  |  |  |  |
| 0 | Vvi-Vitvi12g00445\_t001 |  |  |  |  |  |  |  |  |  |  |  |  |
| 1 | Vvi-Vitvi12g00446\_t001 |  | Atr-ERM96911 |  |  |  |  |  |  |  |  |  |  |
| 1 | Vvi-Vitvi12g00447\_t001 |  | \| \| |  |  |  |  |  |  |  |  |  |  |
| 1 | Vvi-Vitvi12g00448\_t001 |  | \| \| |  |  |  |  |  |  |  |  |  |  |
| 1 | Vvi-Vitvi12g00449\_t001 |  | Atr-ERM96915 |  |  |  |  |  |  |  |  |  |  |
| 1 | Vvi-Vitvi12g00451\_t001 |  | \| \| |  |  |  |  |  |  |  |  |  |  |
| 1 | Vvi-Vitvi12g02381\_t001 |  | Atr-ERM96916 |  |  |  |  |  |  |  |  |  |  |
| 1 | Vvi-Vitvi12g02382\_t001 |  | Atr-ERM96918 |  |  |  |  |  |  |  |  |  |  |
| 1 | Vvi-Vitvi12g02385\_t001 |  | \| \| |  |  |  |  |  |  |  |  |  |  |
| 1 | Vvi-Vitvi12g02384\_t001 |  | \| \| |  |  |  |  |  |  |  |  |  |  |
| 1 | Vvi-Vitvi12g04135\_t001 |  | \| \| |  |  |  |  |  |  |  |  |  |  |
| 1 | Vvi-Vitvi12g04136\_t001 |  | \| \| |  |  |  |  |  |  |  |  |  |  |
| 1 | Vvi-Vitvi12g04137\_t004 |  | \| \| |  |  |  |  |  |  |  |  |  |  |
| 1 | Vvi-Vitvi12g00452\_t001 |  | \| \| |  |  |  |  |  |  |  |  |  |  |
| 1 | Vvi-Vitvi12g00453\_t001 |  | Atr-ERM96921 |  |  |  |  |  |  |  |  |  |  |
| 1 | Vvi-Vitvi12g00454\_t001 |  | Atr-ERM96930 |  |  |  |  |  |  |  |  |  |  |
| 1 | Vvi-Vitvi12g00455\_t001 |  | Atr-ERM96941 |  |  |  |  |  |  |  |  |  |  |
| 1 | Vvi-Vitvi12g04138\_t001 |  | \| \| |  |  |  |  |  |  |  |  |  |  |
| 1 | Vvi-Vitvi12g00456\_t001 |  | \| \| |  |  |  |  |  |  |  |  |  |  |
| 1 | Vvi-Vitvi12g00459\_t001 |  | \| \| |  |  |  |  |  |  |  |  |  |  |
| 1 | Vvi-Vitvi12g04139\_t001 |  | \| \| |  |  |  |  |  |  |  |  |  |  |
| 1 | Vvi-Vitvi12g02387\_t001 |  | Atr-ERM96951 |  |  |  |  |  |  |  |  |  |  |
| 0 | Vvi-Vitvi12g00462\_t001 |  |  |  |  |  |  |  |  |  |  |  |  |
| 0 | Vvi-Vitvi12g00463\_t001 |  |  |  |  |  |  |  |  |  |  |  |  |
| 0 | Vvi-Vitvi12g04140\_t001 |  |  |  |  |  |  |  |  |  |  |  |  |
| 0 | Vvi-Vitvi12g00464\_t001 |  |  |  |  |  |  |  |  |  |  |  |  |
| 0 | Vvi-Vitvi12g00465\_t001 |  |  |  |  |  |  |  |  |  |  |  |  |
| 0 | Vvi-Vitvi12g00466\_t001 |  |  |  |  |  |  |  |  |  |  |  |  |
| 0 | Vvi-Vitvi12g00467\_t001 |  |  |  |  |  |  |  |  |  |  |  |  |
| 1 | Vvi-Vitvi12g00468\_t001 |  | Atr-ERN03346 |  |  |  |  |  |  |  |  |  |  |
| 1 | Vvi-Vitvi12g00469\_t001 |  | Atr-ERN03348 |  |  |  |  |  |  |  |  |  |  |
| 1 | Vvi-Vitvi12g00471\_t001 |  | \| \| |  |  |  |  |  |  |  |  |  |  |
| 1 | Vvi-Vitvi12g02388\_t001 |  | \| \| |  |  |  |  |  |  |  |  |  |  |
| 1 | Vvi-Vitvi12g00472\_t001 |  | Atr-ERN03355 |  |  |  |  |  |  |  |  |  |  |
| 1 | Vvi-Vitvi12g00473\_t001 |  | Atr-ERN03358 |  |  |  |  |  |  |  |  |  |  |
| 1 | Vvi-Vitvi12g02389\_t001 |  | Atr-ERN03365 |  |  |  |  |  |  |  |  |  |  |
| 1 | Vvi-Vitvi12g02390\_t001 |  | \| \| |  |  |  |  |  |  |  |  |  |  |
| 1 | Vvi-Vitvi12g04141\_t001 |  | \| \| |  |  |  |  |  |  |  |  |  |  |
| 1 | Vvi-Vitvi12g04142\_t001 |  | Atr-ERN03366 |  |  |  |  |  |  |  |  |  |  |
| 1 | Vvi-Vitvi12g00474\_t001 |  | \| \| |  |  |  |  |  |  |  |  |  |  |
| 1 | Vvi-Vitvi12g00475\_t001 |  | Atr-ERN03378 |  |  |  |  |  |  |  |  |  |  |
| 1 | Vvi-Vitvi12g04143\_t001 |  | \| \| |  |  |  |  |  |  |  |  |  |  |
| 1 | Vvi-Vitvi12g04144\_t001 |  | \| \| |  |  |  |  |  |  |  |  |  |  |
| 1 | Vvi-Vitvi12g00477\_t003 |  | \| \| |  |  |  |  |  |  |  |  |  |  |
| 1 | Vvi-Vitvi12g02394\_t001 |  | Atr-ERN03381 |  |  |  |  |  |  |  |  |  |  |
| 1 | Vvi-Vitvi12g00478\_t001 |  | Atr-ERN03387 |  |  |  |  |  |  |  |  |  |  |
| 1 | Vvi-Vitvi12g00479\_t001 |  | \| \| |  |  |  |  |  |  |  |  |  |  |
| 1 | Vvi-Vitvi12g00480\_t001 |  | Atr-ERN03404 |  |  |  |  |  |  |  |  |  |  |
| 1 | Vvi-Vitvi12g04145\_t001 |  | \| \| |  |  |  |  |  |  |  |  |  |  |
| 1 | Vvi-Vitvi12g04146\_t001 |  | \| \| |  |  |  |  |  |  |  |  |  |  |
| 1 | Vvi-Vitvi12g00481\_t001 |  | Atr-ERN03410 |  |  |  |  |  |  |  |  |  |  |
| 1 | Vvi-Vitvi12g00482\_t001 |  | Atr-ERN03412 |  |  |  |  |  |  |  |  |  |  |
| 1 | Vvi-Vitvi12g04147\_t001 |  | \| \| |  |  |  |  |  |  |  |  |  |  |
| 1 | Vvi-Vitvi12g00483\_t003 |  | Atr-ERN03417 |  |  |  |  |  |  |  |  |  |  |
| 1 | Vvi-Vitvi12g00484\_t001 |  | Atr-ERN03423 |  |  |  |  |  |  |  |  |  |  |
| 1 | Vvi-Vitvi12g00485\_t001 |  | Atr-ERN03425 |  |  |  |  |  |  |  |  |  |  |
| 1 | Vvi-Vitvi12g00486\_t001 |  | Atr-ERN03430 |  |  |  |  |  |  |  |  |  |  |
| 1 | Vvi-Vitvi12g00489\_t001 |  | Atr-ERN03431 |  |  |  |  |  |  |  |  |  |  |
| 1 | Vvi-Vitvi12g00490\_t001 |  | Atr-ERN03441 |  |  |  |  |  |  |  |  |  |  |
| 1 | Vvi-Vitvi12g00491\_t001 |  | Atr-ERN03444 |  |  |  |  |  |  |  |  |  |  |
| 1 | Vvi-Vitvi12g00493\_t002 |  | Atr-ERN03445 |  |  |  |  |  |  |  |  |  |  |
| 1 | Vvi-Vitvi12g00494\_t001 |  | \| \| |  |  |  |  |  |  |  |  |  |  |
| 1 | Vvi-Vitvi12g00496\_t001 |  | \| \| |  |  |  |  |  |  |  |  |  |  |
| 1 | Vvi-Vitvi12g00497\_t001 |  | \| \| |  |  |  |  |  |  |  |  |  |  |
| 1 | Vvi-Vitvi12g02402\_t001 |  | Atr-ERN03452 |  |  |  |  |  |  |  |  |  |  |
| 1 | Vvi-Vitvi12g00498\_t001 |  | \| \| |  |  |  |  |  |  |  |  |  |  |
| 1 | Vvi-Vitvi12g00499\_t001 |  | \| \| |  |  |  |  |  |  |  |  |  |  |
| 1 | Vvi-Vitvi12g04148\_t001 |  | \| \| |  |  |  |  |  |  |  |  |  |  |
| 1 | Vvi-Vitvi12g04149\_t001 |  | \| \| |  |  |  |  |  |  |  |  |  |  |
| 1 | Vvi-Vitvi12g00500\_t001 |  | \| \| |  |  |  |  |  |  |  |  |  |  |
| 1 | Vvi-Vitvi12g04150\_t001 |  | \| \| |  |  |  |  |  |  |  |  |  |  |
| 1 | Vvi-Vitvi12g00501\_t001 |  | Atr-ERN03456 |  |  |  |  |  |  |  |  |  |  |
| 1 | Vvi-Vitvi12g00502\_t001 |  | Atr-ERN03480 |  |  |  |  |  |  |  |  |  |  |
| 0 | Vvi-Vitvi12g02404\_t001 |  |  |  |  |  |  |  |  |  |  |  |  |
| 0 | Vvi-Vitvi12g00504\_t001 |  |  |  |  |  |  |  |  |  |  |  |  |
| 0 | Vvi-Vitvi12g02405\_t001 |  |  |  |  |  |  |  |  |  |  |  |  |
| 0 | Vvi-Vitvi12g02406\_t001 |  |  |  |  |  |  |  |  |  |  |  |  |
| 0 | Vvi-Vitvi12g02407\_t001 |  |  |  |  |  |  |  |  |  |  |  |  |
| 0 | Vvi-Vitvi12g00505\_t001 |  |  |  |  |  |  |  |  |  |  |  |  |
| 0 | Vvi-Vitvi12g00506\_t001 |  |  |  |  |  |  |  |  |  |  |  |  |
| 0 | Vvi-Vitvi12g02409\_t001 |  |  |  |  |  |  |  |  |  |  |  |  |
| 0 | Vvi-Vitvi12g04151\_t001 |  |  |  |  |  |  |  |  |  |  |  |  |
| 0 | Vvi-Vitvi12g00509\_t001 |  |  |  |  |  |  |  |  |  |  |  |  |
| 0 | Vvi-Vitvi12g04152\_t001 |  |  |  |  |  |  |  |  |  |  |  |  |
| 0 | Vvi-Vitvi12g04153\_t001 |  |  |  |  |  |  |  |  |  |  |  |  |
| 0 | Vvi-Vitvi12g02411\_t001 |  |  |  |  |  |  |  |  |  |  |  |  |
| 0 | Vvi-Vitvi12g04154\_t001 |  |  |  |  |  |  |  |  |  |  |  |  |
| 0 | Vvi-Vitvi12g04155\_t001 |  |  |  |  |  |  |  |  |  |  |  |  |
| 0 | Vvi-Vitvi12g02413\_t001 |  |  |  |  |  |  |  |  |  |  |  |  |
| 0 | Vvi-Vitvi12g00515\_t001 |  |  |  |  |  |  |  |  |  |  |  |  |
| 0 | Vvi-Vitvi12g00516\_t001 |  |  |  |  |  |  |  |  |  |  |  |  |
| 0 | Vvi-Vitvi12g00517\_t001 |  |  |  |  |  |  |  |  |  |  |  |  |
| 0 | Vvi-Vitvi12g00519\_t002 |  |  |  |  |  |  |  |  |  |  |  |  |
| 0 | Vvi-Vitvi12g00520\_t001 |  |  |  |  |  |  |  |  |  |  |  |  |
| 0 | Vvi-Vitvi12g04156\_t001 |  |  |  |  |  |  |  |  |  |  |  |  |
| 0 | Vvi-Vitvi12g00521\_t001 |  |  |  |  |  |  |  |  |  |  |  |  |
| 0 | Vvi-Vitvi12g04157\_t001 |  |  |  |  |  |  |  |  |  |  |  |  |
| 0 | Vvi-Vitvi12g00522\_t001 |  |  |  |  |  |  |  |  |  |  |  |  |
| 0 | Vvi-Vitvi12g02414\_t001 |  |  |  |  |  |  |  |  |  |  |  |  |
| 0 | Vvi-Vitvi12g00523\_t001 |  |  |  |  |  |  |  |  |  |  |  |  |
| 0 | Vvi-Vitvi12g00524\_t001 |  |  |  |  |  |  |  |  |  |  |  |  |
| 0 | Vvi-Vitvi12g00526\_t001 |  |  |  |  |  |  |  |  |  |  |  |  |
| 0 | Vvi-Vitvi12g04158\_t002 |  |  |  |  |  |  |  |  |  |  |  |  |
| 0 | Vvi-Vitvi12g04159\_t001 |  |  |  |  |  |  |  |  |  |  |  |  |
| 0 | Vvi-Vitvi12g00528\_t002 |  |  |  |  |  |  |  |  |  |  |  |  |
| 0 | Vvi-Vitvi12g00529\_t001 |  |  |  |  |  |  |  |  |  |  |  |  |
| 0 | Vvi-Vitvi12g04160\_t001 |  |  |  |  |  |  |  |  |  |  |  |  |
| 0 | Vvi-Vitvi12g04161\_t001 |  |  |  |  |  |  |  |  |  |  |  |  |
| 0 | Vvi-Vitvi12g00530\_t001 |  |  |  |  |  |  |  |  |  |  |  |  |
| 0 | Vvi-Vitvi12g00531\_t001 |  |  |  |  |  |  |  |  |  |  |  |  |
| 0 | Vvi-Vitvi12g00532\_t001 |  |  |  |  |  |  |  |  |  |  |  |  |
| 0 | Vvi-Vitvi12g02415\_t001 |  |  |  |  |  |  |  |  |  |  |  |  |
| 0 | Vvi-Vitvi12g00533\_t001 |  |  |  |  |  |  |  |  |  |  |  |  |
| 0 | Vvi-Vitvi12g02416\_t001 |  |  |  |  |  |  |  |  |  |  |  |  |
| 0 | Vvi-Vitvi12g00534\_t002 |  |  |  |  |  |  |  |  |  |  |  |  |
| 0 | Vvi-Vitvi12g00535\_t001 |  |  |  |  |  |  |  |  |  |  |  |  |
| 0 | Vvi-Vitvi12g00536\_t001 |  |  |  |  |  |  |  |  |  |  |  |  |
| 0 | Vvi-Vitvi12g00537\_t001 |  |  |  |  |  |  |  |  |  |  |  |  |
| 0 | Vvi-Vitvi12g00538\_t001 |  |  |  |  |  |  |  |  |  |  |  |  |
| 0 | Vvi-Vitvi12g00540\_t001 |  |  |  |  |  |  |  |  |  |  |  |  |
| 0 | Vvi-Vitvi12g00541\_t001 |  |  |  |  |  |  |  |  |  |  |  |  |
| 0 | Vvi-Vitvi12g04162\_t001 |  |  |  |  |  |  |  |  |  |  |  |  |
| 0 | Vvi-Vitvi12g00542\_t001 |  |  |  |  |  |  |  |  |  |  |  |  |
| 0 | Vvi-Vitvi12g04163\_t001 |  |  |  |  |  |  |  |  |  |  |  |  |
| 0 | Vvi-Vitvi12g00543\_t001 |  |  |  |  |  |  |  |  |  |  |  |  |
| 0 | Vvi-Vitvi12g00544\_t001 |  |  |  |  |  |  |  |  |  |  |  |  |
| 0 | Vvi-Vitvi12g04164\_t001 |  |  |  |  |  |  |  |  |  |  |  |  |
| 0 | Vvi-Vitvi12g00545\_t001 |  |  |  |  |  |  |  |  |  |  |  |  |
| 1 | Vvi-Vitvi12g00546\_t001 |  | Atr-ERN12728 |  |  |  |  |  |  |  |  |  |  |
| 1 | Vvi-Vitvi12g04165\_t001 |  | \| \| |  |  |  |  |  |  |  |  |  |  |
| 1 | Vvi-Vitvi12g00549\_t001 |  | \| \| |  |  |  |  |  |  |  |  |  |  |
| 1 | Vvi-Vitvi12g04166\_t001 |  | \| \| |  |  |  |  |  |  |  |  |  |  |
| 1 | Vvi-Vitvi12g02417\_t001 |  | \| \| |  |  |  |  |  |  |  |  |  |  |
| 1 | Vvi-Vitvi12g02418\_t001 |  | \| \| |  |  |  |  |  |  |  |  |  |  |
| 1 | Vvi-Vitvi12g04167\_t001 |  | \| \| |  |  |  |  |  |  |  |  |  |  |
| 1 | Vvi-Vitvi12g04168\_t001 |  | \| \| |  |  |  |  |  |  |  |  |  |  |
| 1 | Vvi-Vitvi12g02419\_t001 |  | \| \| |  |  |  |  |  |  |  |  |  |  |
| 1 | Vvi-Vitvi12g04169\_t001 |  | \| \| |  |  |  |  |  |  |  |  |  |  |
| 1 | Vvi-Vitvi12g00552\_t001 |  | \| \| |  |  |  |  |  |  |  |  |  |  |
| 1 | Vvi-Vitvi12g00554\_t001 |  | \| \| |  |  |  |  |  |  |  |  |  |  |
| 1 | Vvi-Vitvi12g04170\_t001 |  | \| \| |  |  |  |  |  |  |  |  |  |  |
| 1 | Vvi-Vitvi12g00555\_t001 |  | \| \| |  |  |  |  |  |  |  |  |  |  |
| 1 | Vvi-Vitvi12g00556\_t002 |  | \| \| |  |  |  |  |  |  |  |  |  |  |
| 1 | Vvi-Vitvi12g02420\_t001 |  | \| \| |  |  |  |  |  |  |  |  |  |  |
| 1 | Vvi-Vitvi12g00558\_t001 |  | \| \| |  |  |  |  |  |  |  |  |  |  |
| 1 | Vvi-Vitvi12g02421\_t001 |  | \| \| |  |  |  |  |  |  |  |  |  |  |
| 1 | Vvi-Vitvi12g00559\_t001 |  | \| \| |  |  |  |  |  |  |  |  |  |  |
| 1 | Vvi-Vitvi12g00560\_t001 |  | \| \| |  |  |  |  |  |  |  |  |  |  |
| 1 | Vvi-Vitvi12g00561\_t001 |  | \| \| |  |  |  |  |  |  |  |  |  |  |
| 1 | Vvi-Vitvi12g00562\_t001 |  | \| \| |  |  |  |  |  |  |  |  |  |  |
| 1 | Vvi-Vitvi12g00563\_t001 |  | \| \| |  |  |  |  |  |  |  |  |  |  |
| 1 | Vvi-Vitvi12g04171\_t001 |  | \| \| |  |  |  |  |  |  |  |  |  |  |
| 1 | Vvi-Vitvi12g00565\_t001 |  | \| \| |  |  |  |  |  |  |  |  |  |  |
| 1 | Vvi-Vitvi12g00566\_t001 |  | \| \| |  |  |  |  |  |  |  |  |  |  |
| 1 | Vvi-Vitvi12g00567\_t001 |  | Atr-ERN12736 |  |  |  |  |  |  |  |  |  |  |
| 1 | Vvi-Vitvi12g00568\_t001 |  | Atr-ERN12742 |  |  |  |  |  |  |  |  |  |  |
| 1 | Vvi-Vitvi12g04172\_t001 |  | \| \| |  |  |  |  |  |  |  |  |  |  |
| 1 | Vvi-Vitvi12g00569\_t002 |  | Atr-ERN12744 |  |  |  |  |  |  |  |  |  |  |
| 1 | Vvi-Vitvi12g02424\_t001 |  | \| \| |  |  |  |  |  |  |  |  |  |  |
| 1 | Vvi-Vitvi12g00571\_t001 |  | Atr-ERN12747 |  |  |  |  |  |  |  |  |  |  |
| 1 | Vvi-Vitvi12g04173\_t001 |  | \| \| |  |  |  |  |  |  |  |  |  |  |
| 1 | Vvi-Vitvi12g04174\_t001 |  | \| \| |  |  |  |  |  |  |  |  |  |  |
| 1 | Vvi-Vitvi12g00572\_t001 |  | Atr-ERN12751 |  |  |  |  |  |  |  |  |  |  |
| 1 | Vvi-Vitvi12g00574\_t001 |  | \| \| |  |  |  |  |  |  |  |  |  |  |
| 1 | Vvi-Vitvi12g00576\_t001 |  | \| \| |  |  |  |  |  |  |  |  |  |  |
| 1 | Vvi-Vitvi12g00577\_t001 |  | \| \| |  |  |  |  |  |  |  |  |  |  |
| 1 | Vvi-Vitvi12g00579\_t001 |  | \| \| |  |  |  |  |  |  |  |  |  |  |
| 1 | Vvi-Vitvi12g02177\_t001 |  | Atr-ERN12759 |  |  |  |  |  |  |  |  |  |  |
| 1 | Vvi-Vitvi12g04175\_t001 |  | \| \| |  |  |  |  |  |  |  |  |  |  |
| 1 | Vvi-Vitvi12g02178\_t001 |  | \| \| |  |  |  |  |  |  |  |  |  |  |
| 1 | Vvi-Vitvi12g00585\_t001 |  | \| \| |  |  |  |  |  |  |  |  |  |  |
| 1 | Vvi-Vitvi12g00586\_t001 |  | \| \| |  |  |  |  |  |  |  |  |  |  |
| 1 | Vvi-Vitvi12g04176\_t001 |  | \| \| |  |  |  |  |  |  |  |  |  |  |
| 1 | Vvi-Vitvi12g04177\_t001 |  | Atr-ERN12772 |  |  |  |  |  |  |  |  |  |  |
| 1 | Vvi-Vitvi12g04178\_t001 |  | \| \| |  |  |  |  |  |  |  |  |  |  |
| 1 | Vvi-Vitvi12g00588\_t001 |  | \| \| |  |  |  |  |  |  |  |  |  |  |
| 1 | Vvi-Vitvi12g04179\_t001 |  | \| \| |  |  |  |  |  |  |  |  |  |  |
| 1 | Vvi-Vitvi12g02430\_t001 |  | \| \| |  |  |  |  |  |  |  |  |  |  |
| 1 | Vvi-Vitvi12g00589\_t001 |  | \| \| |  |  |  |  |  |  |  |  |  |  |
| 1 | Vvi-Vitvi12g00590\_t001 |  | Atr-ERN12780 |  |  |  |  |  |  |  |  |  |  |
| 1 | Vvi-Vitvi12g00591\_t001 |  | \| \| |  |  |  |  |  |  |  |  |  |  |
| 1 | Vvi-Vitvi12g00592\_t001 |  | \| \| |  |  |  |  |  |  |  |  |  |  |
| 1 | Vvi-Vitvi12g00593\_t001 |  | Atr-ERN12786 |  |  |  |  |  |  |  |  |  |  |
| 1 | Vvi-Vitvi12g00594\_t001 |  | \| \| |  |  |  |  |  |  |  |  |  |  |
| 1 | Vvi-Vitvi12g00595\_t001 |  | \| \| |  |  |  |  |  |  |  |  |  |  |
| 1 | Vvi-Vitvi12g04180\_t001 |  | \| \| |  |  |  |  |  |  |  |  |  |  |
| 1 | Vvi-Vitvi12g04181\_t001 |  | \| \| |  |  |  |  |  |  |  |  |  |  |
| 1 | Vvi-Vitvi12g04182\_t001 |  | \| \| |  |  |  |  |  |  |  |  |  |  |
| 1 | Vvi-Vitvi12g04183\_t001 |  | \| \| |  |  |  |  |  |  |  |  |  |  |
| 1 | Vvi-Vitvi12g04184\_t001 |  | \| \| |  |  |  |  |  |  |  |  |  |  |
| 1 | Vvi-Vitvi12g04185\_t001 |  | Atr-ERN12802 |  |  |  |  |  |  |  |  |  |  |
| 0 | Vvi-Vitvi12g00606\_t001 |  |  |  |  |  |  |  |  |  |  |  |  |
| 0 | Vvi-Vitvi12g04186\_t001 |  |  |  |  |  |  |  |  |  |  |  |  |
| 0 | Vvi-Vitvi12g04187\_t001 |  |  |  |  |  |  |  |  |  |  |  |  |
| 0 | Vvi-Vitvi12g00601\_t001 |  |  |  |  |  |  |  |  |  |  |  |  |
| 0 | Vvi-Vitvi12g04188\_t001 |  |  |  |  |  |  |  |  |  |  |  |  |
| 0 | Vvi-Vitvi12g02439\_t001 |  |  |  |  |  |  |  |  |  |  |  |  |
| 0 | Vvi-Vitvi12g04189\_t001 |  |  |  |  |  |  |  |  |  |  |  |  |
| 0 | Vvi-Vitvi12g04190\_t001 |  |  |  |  |  |  |  |  |  |  |  |  |
| 0 | Vvi-Vitvi12g02440\_t001 |  |  |  |  |  |  |  |  |  |  |  |  |
| 0 | Vvi-Vitvi12g04191\_t001 |  |  |  |  |  |  |  |  |  |  |  |  |
| 1 | Vvi-Vitvi12g02441\_t001 |  | Atr-ERM98369 |  |  |  |  |  |  |  |  |  |  |
| 1 | Vvi-Vitvi12g02442\_t001 |  | Atr-ERM98372 |  |  |  |  |  |  |  |  |  |  |
| 1 | Vvi-Vitvi12g04192\_t001 |  | \| \| |  |  |  |  |  |  |  |  |  |  |
| 1 | Vvi-Vitvi12g00619\_t001 |  | Atr-ERM98373 |  |  |  |  |  |  |  |  |  |  |
| 1 | Vvi-Vitvi12g00620\_t002 |  | Atr-ERM98375 |  |  |  |  |  |  |  |  |  |  |
| 1 | Vvi-Vitvi12g00621\_t001 |  | Atr-ERM98376 |  |  |  |  |  |  |  |  |  |  |
| 1 | Vvi-Vitvi12g00622\_t001 |  | Atr-ERM98383 |  |  |  |  |  |  |  |  |  |  |
| 1 | Vvi-Vitvi12g00623\_t001 |  | \| \| |  |  |  |  |  |  |  |  |  |  |
| 1 | Vvi-Vitvi12g00624\_t001 |  | \| \| |  |  |  |  |  |  |  |  |  |  |
| 1 | Vvi-Vitvi12g00625\_t001 |  | \| \| |  |  |  |  |  |  |  |  |  |  |
| 1 | Vvi-Vitvi12g00626\_t001 |  | \| \| |  |  |  |  |  |  |  |  |  |  |
| 1 | Vvi-Vitvi12g00627\_t002 |  | Atr-ERM98399 |  |  |  |  |  |  |  |  |  |  |
| 1 | Vvi-Vitvi12g00628\_t002 |  | Atr-ERM98405 |  |  |  |  |  |  |  |  |  |  |
| 1 | Vvi-Vitvi12g04193\_t001 |  | \| \| |  |  |  |  |  |  |  |  |  |  |
| 1 | Vvi-Vitvi12g04194\_t001 |  | \| \| |  |  |  |  |  |  |  |  |  |  |
| 1 | Vvi-Vitvi12g00630\_t001 |  | Atr-ERM98409 |  |  |  |  |  |  |  |  |  |  |
| 1 | Vvi-Vitvi12g00631\_t001 |  | Atr-ERN03542 |  |  |  |  |  |  |  |  |  |  |
| 1 | Vvi-Vitvi12g04195\_t001 |  | \| \| |  |  |  |  |  |  |  |  |  |  |
| 1 | Vvi-Vitvi12g00632\_t001 |  | Atr-ERN03536 |  |  |  |  |  |  |  |  |  |  |
| 1 | Vvi-Vitvi12g02443\_t001 |  | \| \| |  |  |  |  |  |  |  |  |  |  |
| 1 | Vvi-Vitvi12g02444\_t001 |  | \| \| |  |  |  |  |  |  |  |  |  |  |
| 1 | Vvi-Vitvi12g04196\_t001 |  | \| \| |  |  |  |  |  |  |  |  |  |  |
| 1 | Vvi-Vitvi12g04197\_t001 |  | \| \| |  |  |  |  |  |  |  |  |  |  |
| 1 | Vvi-Vitvi12g04198\_t001 |  | \| \| |  |  |  |  |  |  |  |  |  |  |
| 1 | Vvi-Vitvi12g02448\_t001 |  | \| \| |  |  |  |  |  |  |  |  |  |  |
| 1 | Vvi-Vitvi12g02449\_t001 |  | \| \| |  |  |  |  |  |  |  |  |  |  |
| 1 | Vvi-Vitvi12g02450\_t001 |  | \| \| |  |  |  |  |  |  |  |  |  |  |
| 1 | Vvi-Vitvi12g02451\_t001 |  | \| \| |  |  |  |  |  |  |  |  |  |  |
| 1 | Vvi-Vitvi12g02452\_t001 |  | \| \| |  |  |  |  |  |  |  |  |  |  |
| 1 | Vvi-Vitvi12g04199\_t001 |  | \| \| |  |  |  |  |  |  |  |  |  |  |
| 1 | Vvi-Vitvi12g02454\_t001 |  | \| \| |  |  |  |  |  |  |  |  |  |  |
| 1 | Vvi-Vitvi12g04200\_t001 |  | \| \| |  |  |  |  |  |  |  |  |  |  |
| 1 | Vvi-Vitvi12g00633\_t003 |  | Atr-ERN03531 |  |  |  |  |  |  |  |  |  |  |
| 1 | Vvi-Vitvi12g00634\_t001 |  | Atr-ERN03530 |  |  |  |  |  |  |  |  |  |  |
| 1 | Vvi-Vitvi12g04201\_t001 |  | \| \| |  |  |  |  |  |  |  |  |  |  |
| 1 | Vvi-Vitvi12g00636\_t001 |  | \| \| |  |  |  |  |  |  |  |  |  |  |
| 1 | Vvi-Vitvi12g02455\_t001 |  | \| \| |  |  |  |  |  |  |  |  |  |  |
| 1 | Vvi-Vitvi12g00637\_t001 |  | \| \| |  |  |  |  |  |  |  |  |  |  |
| 1 | Vvi-Vitvi12g02456\_t001 |  | \| \| |  |  |  |  |  |  |  |  |  |  |
| 1 | Vvi-Vitvi12g02457\_t001 |  | Atr-ERN03528 |  |  |  |  |  |  |  |  |  |  |
| 1 | Vvi-Vitvi12g02458\_t001 |  | \| \| |  |  |  |  |  |  |  |  |  |  |
| 1 | Vvi-Vitvi12g02459\_t001 |  | \| \| |  |  |  |  |  |  |  |  |  |  |
| 1 | Vvi-Vitvi12g04202\_t001 |  | \| \| |  |  |  |  |  |  |  |  |  |  |
| 1 | Vvi-Vitvi12g04203\_t001 |  | \| \| |  |  |  |  |  |  |  |  |  |  |
| 1 | Vvi-Vitvi12g02461\_t001 |  | \| \| |  |  |  |  |  |  |  |  |  |  |
| 1 | Vvi-Vitvi12g04204\_t001 |  | \| \| |  |  |  |  |  |  |  |  |  |  |
| 1 | Vvi-Vitvi12g04205\_t001 |  | \| \| |  |  |  |  |  |  |  |  |  |  |
| 1 | Vvi-Vitvi12g04206\_t001 |  | \| \| |  |  |  |  |  |  |  |  |  |  |
| 1 | Vvi-Vitvi12g04207\_t001 |  | \| \| |  |  |  |  |  |  |  |  |  |  |
| 1 | Vvi-Vitvi12g02465\_t001 |  | \| \| |  |  |  |  |  |  |  |  |  |  |
| 1 | Vvi-Vitvi12g02466\_t001 |  | \| \| |  |  |  |  |  |  |  |  |  |  |
| 1 | Vvi-Vitvi12g02467\_t001 |  | \| \| |  |  |  |  |  |  |  |  |  |  |
| 1 | Vvi-Vitvi12g02468\_t001 |  | \| \| |  |  |  |  |  |  |  |  |  |  |
| 1 | Vvi-Vitvi12g00638\_t001 |  | Atr-ERN03523 |  |  |  |  |  |  |  |  |  |  |
| 1 | Vvi-Vitvi12g04208\_t001 |  | \| \| |  |  |  |  |  |  |  |  |  |  |
| 1 | Vvi-Vitvi12g02470\_t001 |  | \| \| |  |  |  |  |  |  |  |  |  |  |
| 1 | Vvi-Vitvi12g00639\_t001 |  | \| \| |  |  |  |  |  |  |  |  |  |  |
| 1 | Vvi-Vitvi12g00640\_t001 |  | Atr-ERN03511 |  |  |  |  |  |  |  |  |  |  |
| 1 | Vvi-Vitvi12g00641\_t001 |  | Atr-ERN03499 |  |  |  |  |  |  |  |  |  |  |
| 1 | Vvi-Vitvi12g00642\_t001 |  | \| \| |  |  |  |  |  |  |  |  |  |  |
| 1 | Vvi-Vitvi12g04209\_t001 |  | \| \| |  |  |  |  |  |  |  |  |  |  |
| 1 | Vvi-Vitvi12g04210\_t001 |  | \| \| |  |  |  |  |  |  |  |  |  |  |
| 1 | Vvi-Vitvi12g02471\_t001 |  | \| \| |  |  |  |  |  |  |  |  |  |  |
| 1 | Vvi-Vitvi12g04211\_t001 |  | \| \| |  |  |  |  |  |  |  |  |  |  |
| 1 | Vvi-Vitvi12g00643\_t001 |  | Atr-ERN03495 |  |  |  |  |  |  |  |  |  |  |
| 0 | Vvi-Vitvi12g02472\_t001 |  |  |  |  |  |  |  |  |  |  |  |  |
| 0 | Vvi-Vitvi12g02473\_t001 |  |  |  |  |  |  |  |  |  |  |  |  |
| 0 | Vvi-Vitvi12g04212\_t001 |  |  |  |  |  |  |  |  |  |  |  |  |
| 0 | Vvi-Vitvi12g02474\_t001 |  |  |  |  |  |  |  |  |  |  |  |  |
| 0 | Vvi-Vitvi12g04213\_t001 |  |  |  |  |  |  |  |  |  |  |  |  |
| 0 | Vvi-Vitvi12g04214\_t001 |  |  |  |  |  |  |  |  |  |  |  |  |
| 0 | Vvi-Vitvi12g00645\_t001 |  |  |  |  |  |  |  |  |  |  |  |  |
| 0 | Vvi-Vitvi12g00646\_t001 |  |  |  |  |  |  |  |  |  |  |  |  |
| 0 | Vvi-Vitvi12g00647\_t001 |  |  |  |  |  |  |  |  |  |  |  |  |
| 0 | Vvi-Vitvi12g00648\_t001 |  |  |  |  |  |  |  |  |  |  |  |  |
| 0 | Vvi-Vitvi12g00649\_t001 |  |  |  |  |  |  |  |  |  |  |  |  |
| 0 | Vvi-Vitvi12g04215\_t001 |  |  |  |  |  |  |  |  |  |  |  |  |
| 0 | Vvi-Vitvi12g00650\_t001 |  |  |  |  |  |  |  |  |  |  |  |  |
| 0 | Vvi-Vitvi12g00651\_t001 |  |  |  |  |  |  |  |  |  |  |  |  |
| 0 | Vvi-Vitvi12g02478\_t001 |  |  |  |  |  |  |  |  |  |  |  |  |
| 0 | Vvi-Vitvi12g00654\_t001 |  |  |  |  |  |  |  |  |  |  |  |  |
| 0 | Vvi-Vitvi12g00655\_t001 |  |  |  |  |  |  |  |  |  |  |  |  |
| 1 | Vvi-Vitvi12g00656\_t001 |  | Atr-ERM99635 |  |  |  |  |  |  |  |  |  |  |
| 1 | Vvi-Vitvi12g04216\_t001 |  | \| \| |  |  |  |  |  |  |  |  |  |  |
| 1 | Vvi-Vitvi12g00657\_t001 |  | \| \| |  |  |  |  |  |  |  |  |  |  |
| 1 | Vvi-Vitvi12g00658\_t001 |  | \| \| |  |  |  |  |  |  |  |  |  |  |
| 1 | Vvi-Vitvi12g04217\_t001 |  | Atr-ERM99631 |  |  |  |  |  |  |  |  |  |  |
| 1 | Vvi-Vitvi12g04218\_t001 |  | \| \| |  |  |  |  |  |  |  |  |  |  |
| 1 | Vvi-Vitvi12g00661\_t001 |  | \| \| |  |  |  |  |  |  |  |  |  |  |
| 1 | Vvi-Vitvi12g00662\_t001 |  | \| \| |  |  |  |  |  |  |  |  |  |  |
| 1 | Vvi-Vitvi12g00664\_t003 |  | Atr-ERM99620 |  |  |  |  |  |  |  |  |  |  |
| 1 | Vvi-Vitvi12g00665\_t002 |  | Atr-ERM99617 |  |  |  |  |  |  |  |  |  |  |
| 1 | Vvi-Vitvi12g00667\_t001 |  | Atr-ERM99613 |  |  |  |  |  |  |  |  |  |  |
| 1 | Vvi-Vitvi12g04219\_t001 |  | \| \| |  |  |  |  |  |  |  |  |  |  |
| 1 | Vvi-Vitvi12g04220\_t001 |  | \| \| |  |  |  |  |  |  |  |  |  |  |
| 1 | Vvi-Vitvi12g04221\_t001 |  | \| \| |  |  |  |  |  |  |  |  |  |  |
| 1 | Vvi-Vitvi12g04222\_t001 |  | \| \| |  |  |  |  |  |  |  |  |  |  |
| 1 | Vvi-Vitvi12g00670\_t001 |  | Atr-ERM99609 |  |  |  |  |  |  |  |  |  |  |
| 1 | Vvi-Vitvi12g00671\_t001 |  | Atr-ERM99608 |  |  |  |  |  |  |  |  |  |  |
| 1 | Vvi-Vitvi12g00672\_t001 |  | \| \| |  |  |  |  |  |  |  |  |  |  |
| 1 | Vvi-Vitvi12g00673\_t001 |  | \| \| |  |  |  |  |  |  |  |  |  |  |
| 1 | Vvi-Vitvi12g04223\_t001 |  | \| \| |  |  |  |  |  |  |  |  |  |  |
| 1 | Vvi-Vitvi12g00675\_t001 |  | \| \| |  |  |  |  |  |  |  |  |  |  |
| 1 | Vvi-Vitvi12g00676\_t001 |  | \| \| |  |  |  |  |  |  |  |  |  |  |
| 1 | Vvi-Vitvi12g04224\_t001 |  | \| \| |  |  |  |  |  |  |  |  |  |  |
| 1 | Vvi-Vitvi12g00678\_t001 |  | \| \| |  |  |  |  |  |  |  |  |  |  |
| 1 | Vvi-Vitvi12g00679\_t001 |  | \| \| |  |  |  |  |  |  |  |  |  |  |
| 1 | Vvi-Vitvi12g04225\_t001 |  | \| \| |  |  |  |  |  |  |  |  |  |  |
| 1 | Vvi-Vitvi12g02485\_t001 |  | Atr-ERM99607 |  |  |  |  |  |  |  |  |  |  |
| 0 | Vvi-Vitvi12g00681\_t001 |  |  |  |  |  |  |  |  |  |  |  |  |
| 0 | Vvi-Vitvi12g00682\_t001 |  |  |  |  |  |  |  |  |  |  |  |  |
| 0 | Vvi-Vitvi12g02486\_t001 |  |  |  |  |  |  |  |  |  |  |  |  |
| 0 | Vvi-Vitvi12g00683\_t001 |  |  |  |  |  |  |  |  |  |  |  |  |
| 0 | Vvi-Vitvi12g00684\_t002 |  |  |  |  |  |  |  |  |  |  |  |  |
| 0 | Vvi-Vitvi12g00685\_t001 |  |  |  |  |  |  |  |  |  |  |  |  |
| 0 | Vvi-Vitvi12g00686\_t001 |  |  |  |  |  |  |  |  |  |  |  |  |
| 0 | Vvi-Vitvi12g00687\_t001 |  |  |  |  |  |  |  |  |  |  |  |  |
| 0 | Vvi-Vitvi12g00688\_t001 |  |  |  |  |  |  |  |  |  |  |  |  |
| 0 | Vvi-Vitvi12g00689\_t001 |  |  |  |  |  |  |  |  |  |  |  |  |
| 0 | Vvi-Vitvi12g04226\_t001 |  |  |  |  |  |  |  |  |  |  |  |  |
| 0 | Vvi-Vitvi12g00690\_t001 |  |  |  |  |  |  |  |  |  |  |  |  |
| 0 | Vvi-Vitvi12g04227\_t001 |  |  |  |  |  |  |  |  |  |  |  |  |
| 0 | Vvi-Vitvi12g00691\_t001 |  |  |  |  |  |  |  |  |  |  |  |  |
| 0 | Vvi-Vitvi12g04228\_t001 |  |  |  |  |  |  |  |  |  |  |  |  |
| 0 | Vvi-Vitvi12g00692\_t001 |  |  |  |  |  |  |  |  |  |  |  |  |
| 0 | Vvi-Vitvi12g04229\_t001 |  |  |  |  |  |  |  |  |  |  |  |  |
| 0 | Vvi-Vitvi12g00694\_t001 |  |  |  |  |  |  |  |  |  |  |  |  |
| 0 | Vvi-Vitvi12g04230\_t001 |  |  |  |  |  |  |  |  |  |  |  |  |
| 0 | Vvi-Vitvi12g04231\_t001 |  |  |  |  |  |  |  |  |  |  |  |  |
| 0 | Vvi-Vitvi12g04232\_t001 |  |  |  |  |  |  |  |  |  |  |  |  |
| 0 | Vvi-Vitvi12g04233\_t001 |  |  |  |  |  |  |  |  |  |  |  |  |
| 0 | Vvi-Vitvi12g04234\_t001 |  |  |  |  |  |  |  |  |  |  |  |  |
| 0 | Vvi-Vitvi12g02489\_t001 |  |  |  |  |  |  |  |  |  |  |  |  |
| 0 | Vvi-Vitvi12g04235\_t001 |  |  |  |  |  |  |  |  |  |  |  |  |
| 0 | Vvi-Vitvi12g04236\_t001 |  |  |  |  |  |  |  |  |  |  |  |  |
| 0 | Vvi-Vitvi12g04237\_t001 |  |  |  |  |  |  |  |  |  |  |  |  |
| 0 | Vvi-Vitvi12g02491\_t001 |  |  |  |  |  |  |  |  |  |  |  |  |
| 0 | Vvi-Vitvi12g04238\_t001 |  |  |  |  |  |  |  |  |  |  |  |  |
| 0 | Vvi-Vitvi12g04239\_t001 |  |  |  |  |  |  |  |  |  |  |  |  |
| 0 | Vvi-Vitvi12g02492\_t001 |  |  |  |  |  |  |  |  |  |  |  |  |
| 0 | Vvi-Vitvi12g04240\_t001 |  |  |  |  |  |  |  |  |  |  |  |  |
| 0 | Vvi-Vitvi12g00704\_t001 |  |  |  |  |  |  |  |  |  |  |  |  |
| 0 | Vvi-Vitvi12g00705\_t001 |  |  |  |  |  |  |  |  |  |  |  |  |
| 0 | Vvi-Vitvi12g00706\_t001 |  |  |  |  |  |  |  |  |  |  |  |  |
| 0 | Vvi-Vitvi12g00707\_t001 |  |  |  |  |  |  |  |  |  |  |  |  |
| 0 | Vvi-Vitvi12g00709\_t001 |  |  |  |  |  |  |  |  |  |  |  |  |
| 0 | Vvi-Vitvi12g00710\_t001 |  |  |  |  |  |  |  |  |  |  |  |  |
| 0 | Vvi-Vitvi12g00711\_t001 |  |  |  |  |  |  |  |  |  |  |  |  |
| 0 | Vvi-Vitvi12g02493\_t001 |  |  |  |  |  |  |  |  |  |  |  |  |
| 0 | Vvi-Vitvi12g00713\_t001 |  |  |  |  |  |  |  |  |  |  |  |  |
| 0 | Vvi-Vitvi12g00714\_t001 |  |  |  |  |  |  |  |  |  |  |  |  |
| 0 | Vvi-Vitvi12g00715\_t001 |  |  |  |  |  |  |  |  |  |  |  |  |
| 0 | Vvi-Vitvi12g00716\_t001 |  |  |  |  |  |  |  |  |  |  |  |  |
| 0 | Vvi-Vitvi12g00717\_t001 |  |  |  |  |  |  |  |  |  |  |  |  |
| 0 | Vvi-Vitvi12g00718\_t001 |  |  |  |  |  |  |  |  |  |  |  |  |
| 0 | Vvi-Vitvi12g02494\_t001 |  |  |  |  |  |  |  |  |  |  |  |  |
| 1 | Vvi-Vitvi12g00719\_t001 |  | Atr-ERN19273 |  |  |  |  |  |  |  |  |  |  |
| 1 | Vvi-Vitvi12g00721\_t001 |  | \| \| |  |  |  |  |  |  |  |  |  |  |
| 1 | Vvi-Vitvi12g00722\_t001 |  | \| \| |  |  |  |  |  |  |  |  |  |  |
| 1 | Vvi-Vitvi12g04241\_t001 |  | \| \| |  |  |  |  |  |  |  |  |  |  |
| 1 | Vvi-Vitvi12g04242\_t001 |  | \| \| |  |  |  |  |  |  |  |  |  |  |
| 1 | Vvi-Vitvi12g04243\_t001 |  | \| \| |  |  |  |  |  |  |  |  |  |  |
| 1 | Vvi-Vitvi12g04244\_t001 |  | \| \| |  |  |  |  |  |  |  |  |  |  |
| 1 | Vvi-Vitvi12g02496\_t001 |  | \| \| |  |  |  |  |  |  |  |  |  |  |
| 1 | Vvi-Vitvi12g00725\_t001 |  | Atr-ERN19251 |  |  |  |  |  |  |  |  |  |  |
| 1 | Vvi-Vitvi12g04245\_t001 |  | \| \| |  |  |  |  |  |  |  |  |  |  |
| 1 | Vvi-Vitvi12g00727\_t001 |  | \| \| |  |  |  |  |  |  |  |  |  |  |
| 1 | Vvi-Vitvi12g04246\_t001 |  | \| \| |  |  |  |  |  |  |  |  |  |  |
| 1 | Vvi-Vitvi12g02498\_t002 |  | \| \| |  |  |  |  |  |  |  |  |  |  |
| 1 | Vvi-Vitvi12g02499\_t003 |  | \| \| |  |  |  |  |  |  |  |  |  |  |
| 1 | Vvi-Vitvi12g04247\_t001 |  | \| \| |  |  |  |  |  |  |  |  |  |  |
| 1 | Vvi-Vitvi12g04248\_t001 |  | \| \| |  |  |  |  |  |  |  |  |  |  |
| 1 | Vvi-Vitvi12g04249\_t001 |  | \| \| |  |  |  |  |  |  |  |  |  |  |
| 1 | Vvi-Vitvi12g00730\_t001 |  | \| \| |  |  |  |  |  |  |  |  |  |  |
| 1 | Vvi-Vitvi12g02500\_t001 |  | \| \| |  |  |  |  |  |  |  |  |  |  |
| 1 | Vvi-Vitvi12g04250\_t001 |  | \| \| |  |  |  |  |  |  |  |  |  |  |
| 1 | Vvi-Vitvi12g04251\_t001 |  | \| \| |  |  |  |  |  |  |  |  |  |  |
| 1 | Vvi-Vitvi12g00736\_t001 |  | Atr-ERN19236 |  |  |  |  |  |  |  |  |  |  |
| 1 | Vvi-Vitvi12g00738\_t001 |  | \| \| |  |  |  |  |  |  |  |  |  |  |
| 1 | Vvi-Vitvi12g02504\_t002 |  | \| \| |  |  |  |  |  |  |  |  |  |  |
| 1 | Vvi-Vitvi12g04252\_t001 |  | Atr-ERN19234 |  |  |  |  |  |  |  |  |  |  |
| 1 | Vvi-Vitvi12g00741\_t001 |  | Atr-ERN19232 |  |  |  |  |  |  |  |  |  |  |
| 1 | Vvi-Vitvi12g00742\_t001 |  | Atr-ERN19226 |  |  |  |  |  |  |  |  |  |  |
| 1 | Vvi-Vitvi12g04253\_t001 |  | \| \| |  |  |  |  |  |  |  |  |  |  |
| 1 | Vvi-Vitvi12g00744\_t001 |  | \| \| |  |  |  |  |  |  |  |  |  |  |
| 1 | Vvi-Vitvi12g00745\_t001 |  | Atr-ERN19224 |  |  |  |  |  |  |  |  |  |  |
| 1 | Vvi-Vitvi12g00746\_t001 |  | Atr-ERN19223 |  |  |  |  |  |  |  |  |  |  |
| 1 | Vvi-Vitvi12g00747\_t001 |  | Atr-ERN19222 |  |  |  |  |  |  |  |  |  |  |
| 1 | Vvi-Vitvi12g02505\_t001 |  | Atr-ERN19221 |  |  |  |  |  |  |  |  |  |  |
| 1 | Vvi-Vitvi12g04254\_t001 |  | \| \| |  |  |  |  |  |  |  |  |  |  |
| 1 | Vvi-Vitvi12g00750\_t001 |  | Atr-ERN19207 |  |  |  |  |  |  |  |  |  |  |
| 1 | Vvi-Vitvi12g00751\_t001 |  | Atr-ERN19206 |  |  |  |  |  |  |  |  |  |  |
| 1 | Vvi-Vitvi12g02506\_t001 |  | \| \| |  |  |  |  |  |  |  |  |  |  |
| 1 | Vvi-Vitvi12g00752\_t001 |  | Atr-ERN19200 |  |  |  |  |  |  |  |  |  |  |
| 1 | Vvi-Vitvi12g00753\_t001 |  | Atr-ERN19197 |  |  |  |  |  |  |  |  |  |  |
| 1 | Vvi-Vitvi12g00754\_t001 |  | Atr-ERN19195 |  |  |  |  |  |  |  |  |  |  |
| 1 | Vvi-Vitvi12g00755\_t001 |  | Atr-ERN19177 |  |  |  |  |  |  |  |  |  |  |
| 1 | Vvi-Vitvi12g00756\_t001 |  | \| \| |  |  |  |  |  |  |  |  |  |  |
| 1 | Vvi-Vitvi12g00757\_t001 |  | Atr-ERN19175 |  |  |  |  |  |  |  |  |  |  |
| 1 | Vvi-Vitvi12g04255\_t001 |  | \| \| |  |  |  |  |  |  |  |  |  |  |
| 1 | Vvi-Vitvi12g00759\_t001 |  | Atr-ERN19174 |  |  |  |  |  |  |  |  |  |  |
| 1 | Vvi-Vitvi12g00760\_t001 |  | Atr-ERN19169 |  |  |  |  |  |  |  |  |  |  |
| 1 | Vvi-Vitvi12g00761\_t001 |  | Atr-ERN19166 |  |  |  |  |  |  |  |  |  |  |
| 1 | Vvi-Vitvi12g00762\_t001 |  | Atr-ERN19165 |  |  |  |  |  |  |  |  |  |  |
| 1 | Vvi-Vitvi12g00763\_t001 |  | Atr-ERN19147 |  |  |  |  |  |  |  |  |  |  |
| 1 | Vvi-Vitvi12g02507\_t001 |  | Atr-ERN19130 |  |  |  |  |  |  |  |  |  |  |
| 1 | Vvi-Vitvi12g00764\_t001 |  | \| \| |  |  |  |  |  |  |  |  |  |  |
| 1 | Vvi-Vitvi12g00765\_t001 |  | \| \| |  |  |  |  |  |  |  |  |  |  |
| 1 | Vvi-Vitvi12g02508\_t001 |  | Atr-ERN19122 |  |  |  |  |  |  |  |  |  |  |
| 1 | Vvi-Vitvi12g02509\_t001 |  | \| \| |  |  |  |  |  |  |  |  |  |  |
| 1 | Vvi-Vitvi12g00766\_t001 |  | Atr-ERN19112 |  |  |  |  |  |  |  |  |  |  |
| 1 | Vvi-Vitvi12g00767\_t001 |  | Atr-ERN19107 |  |  |  |  |  |  |  |  |  |  |
| 1 | Vvi-Vitvi12g00769\_t001 |  | Atr-ERN19106 |  |  |  |  |  |  |  |  |  |  |
| 1 | Vvi-Vitvi12g00770\_t001 |  | Atr-ERN19087 |  |  |  |  |  |  |  |  |  |  |
| 1 | Vvi-Vitvi12g00771\_t001 |  | \| \| |  |  |  |  |  |  |  |  |  |  |
| 1 | Vvi-Vitvi12g00772\_t001 |  | \| \| |  |  |  |  |  |  |  |  |  |  |
| 1 | Vvi-Vitvi12g00773\_t001 |  | Atr-ERN19072 |  |  |  |  |  |  |  |  |  |  |
| 1 | Vvi-Vitvi12g00774\_t001 |  | Atr-ERN19071 |  |  |  |  |  |  |  |  |  |  |
| 1 | Vvi-Vitvi12g04256\_t001 |  | \| \| |  |  |  |  |  |  |  |  |  |  |
| 1 | Vvi-Vitvi12g02510\_t001 |  | Atr-ERN19064 |  |  |  |  |  |  |  |  |  |  |
| 1 | Vvi-Vitvi12g00777\_t001 |  | Atr-ERN19059 |  |  |  |  |  |  |  |  |  |  |
| 0 | Vvi-Vitvi12g04257\_t001 |  |  |  |  |  |  |  |  |  |  |  |  |
| 0 | Vvi-Vitvi12g00779\_t001 |  |  |  |  |  |  |  |  |  |  |  |  |
| 0 | Vvi-Vitvi12g00782\_t001 |  |  |  |  |  |  |  |  |  |  |  |  |
| 0 | Vvi-Vitvi12g04258\_t001 |  |  |  |  |  |  |  |  |  |  |  |  |
| 0 | Vvi-Vitvi12g04259\_t001 |  |  |  |  |  |  |  |  |  |  |  |  |
| 0 | Vvi-Vitvi12g02511\_t001 |  |  |  |  |  |  |  |  |  |  |  |  |
| 0 | Vvi-Vitvi12g04260\_t001 |  |  |  |  |  |  |  |  |  |  |  |  |
| 0 | Vvi-Vitvi12g04261\_t001 |  |  |  |  |  |  |  |  |  |  |  |  |
| 0 | Vvi-Vitvi12g04262\_t001 |  |  |  |  |  |  |  |  |  |  |  |  |
| 0 | Vvi-Vitvi12g04264\_t001 |  |  |  |  |  |  |  |  |  |  |  |  |
| 0 | Vvi-Vitvi12g04265\_t001 |  |  |  |  |  |  |  |  |  |  |  |  |
| 0 | Vvi-Vitvi12g04266\_t001 |  |  |  |  |  |  |  |  |  |  |  |  |
| 0 | Vvi-Vitvi12g00840\_t001 |  |  |  |  |  |  |  |  |  |  |  |  |
| 0 | Vvi-Vitvi12g00838\_t001 |  |  |  |  |  |  |  |  |  |  |  |  |
| 0 | Vvi-Vitvi12g00837\_t003 |  |  |  |  |  |  |  |  |  |  |  |  |
| 0 | Vvi-Vitvi12g04267\_t001 |  |  |  |  |  |  |  |  |  |  |  |  |
| 0 | Vvi-Vitvi12g02525\_t001 |  |  |  |  |  |  |  |  |  |  |  |  |
| 0 | Vvi-Vitvi12g04268\_t001 |  |  |  |  |  |  |  |  |  |  |  |  |
| 0 | Vvi-Vitvi12g04269\_t001 |  |  |  |  |  |  |  |  |  |  |  |  |
| 0 | Vvi-Vitvi12g04270\_t001 |  |  |  |  |  |  |  |  |  |  |  |  |
| 0 | Vvi-Vitvi12g04271\_t001 |  |  |  |  |  |  |  |  |  |  |  |  |
| 0 | Vvi-Vitvi12g00830\_t001 |  |  |  |  |  |  |  |  |  |  |  |  |
| 0 | Vvi-Vitvi12g02523\_t001 |  |  |  |  |  |  |  |  |  |  |  |  |
| 0 | Vvi-Vitvi12g04272\_t001 |  |  |  |  |  |  |  |  |  |  |  |  |
| 0 | Vvi-Vitvi12g02520\_t001 |  |  |  |  |  |  |  |  |  |  |  |  |
| 0 | Vvi-Vitvi12g00828\_t001 |  |  |  |  |  |  |  |  |  |  |  |  |
| 0 | Vvi-Vitvi12g00825\_t004 |  |  |  |  |  |  |  |  |  |  |  |  |
| 0 | Vvi-Vitvi12g00823\_t001 |  |  |  |  |  |  |  |  |  |  |  |  |
| 0 | Vvi-Vitvi12g00822\_t001 |  |  |  |  |  |  |  |  |  |  |  |  |
| 0 | Vvi-Vitvi12g04273\_t001 |  |  |  |  |  |  |  |  |  |  |  |  |
| 0 | Vvi-Vitvi12g04274\_t001 |  |  |  |  |  |  |  |  |  |  |  |  |
| 1 | Vvi-Vitvi12g00857\_t001 |  | Atr-ERN14647 |  |  |  |  |  |  |  |  |  |  |
| 1 | Vvi-Vitvi12g04275\_t001 |  | \| \| |  |  |  |  |  |  |  |  |  |  |
| 1 | Vvi-Vitvi12g04276\_t001 |  | \| \| |  |  |  |  |  |  |  |  |  |  |
| 1 | Vvi-Vitvi12g02532\_t001 |  | \| \| |  |  |  |  |  |  |  |  |  |  |
| 1 | Vvi-Vitvi12g00858\_t001 |  | \| \| |  |  |  |  |  |  |  |  |  |  |
| 1 | Vvi-Vitvi12g04277\_t001 |  | \| \| |  |  |  |  |  |  |  |  |  |  |
| 1 | Vvi-Vitvi12g00864\_t001 |  | \| \| |  |  |  |  |  |  |  |  |  |  |
| 1 | Vvi-Vitvi12g00866\_t001 |  | Atr-ERN14633 |  |  |  |  |  |  |  |  |  |  |
| 1 | Vvi-Vitvi12g04278\_t001 |  | \| \| |  |  |  |  |  |  |  |  |  |  |
| 1 | Vvi-Vitvi12g00868\_t002 |  | \| \| |  |  |  |  |  |  |  |  |  |  |
| 1 | Vvi-Vitvi12g02534\_t001 |  | \| \| |  |  |  |  |  |  |  |  |  |  |
| 1 | Vvi-Vitvi12g04279\_t001 |  | \| \| |  |  |  |  |  |  |  |  |  |  |
| 1 | Vvi-Vitvi12g04280\_t001 |  | \| \| |  |  |  |  |  |  |  |  |  |  |
| 1 | Vvi-Vitvi12g02536\_t001 |  | \| \| |  |  |  |  |  |  |  |  |  |  |
| 1 | Vvi-Vitvi12g04281\_t001 |  | \| \| |  |  |  |  |  |  |  |  |  |  |
| 1 | Vvi-Vitvi12g04282\_t001 |  | \| \| |  |  |  |  |  |  |  |  |  |  |
| 1 | Vvi-Vitvi12g02545\_t001 |  | \| \| |  |  |  |  |  |  |  |  |  |  |
| 1 | Vvi-Vitvi12g04283\_t001 |  | \| \| |  |  |  |  |  |  |  |  |  |  |
| 1 | Vvi-Vitvi12g04284\_t001 |  | \| \| |  |  |  |  |  |  |  |  |  |  |
| 1 | Vvi-Vitvi12g04285\_t001 |  | \| \| |  |  |  |  |  |  |  |  |  |  |
| 1 | Vvi-Vitvi12g02551\_t001 |  | \| \| |  |  |  |  |  |  |  |  |  |  |
| 1 | Vvi-Vitvi12g04286\_t001 |  | \| \| |  |  |  |  |  |  |  |  |  |  |
| 1 | Vvi-Vitvi12g04287\_t001 |  | \| \| |  |  |  |  |  |  |  |  |  |  |
| 1 | Vvi-Vitvi12g04288\_t001 |  | \| \| |  |  |  |  |  |  |  |  |  |  |
| 1 | Vvi-Vitvi12g04289\_t001 |  | \| \| |  |  |  |  |  |  |  |  |  |  |
| 1 | Vvi-Vitvi12g00905\_t001 |  | Atr-ERN14619 |  |  |  |  |  |  |  |  |  |  |
| 1 | Vvi-Vitvi12g04290\_t001 |  | \| \| |  |  |  |  |  |  |  |  |  |  |
| 1 | Vvi-Vitvi12g04293\_t001 |  | \| \| |  |  |  |  |  |  |  |  |  |  |
| 1 | Vvi-Vitvi12g04294\_t001 |  | \| \| |  |  |  |  |  |  |  |  |  |  |
| 1 | Vvi-Vitvi12g00910\_t001 |  | \| \| |  |  |  |  |  |  |  |  |  |  |
| 1 | Vvi-Vitvi12g00912\_t001 |  | \| \| |  |  |  |  |  |  |  |  |  |  |
| 1 | Vvi-Vitvi12g00913\_t001 |  | \| \| |  |  |  |  |  |  |  |  |  |  |
| 1 | Vvi-Vitvi12g04295\_t001 |  | \| \| |  |  |  |  |  |  |  |  |  |  |
| 1 | Vvi-Vitvi12g04296\_t001 |  | \| \| |  |  |  |  |  |  |  |  |  |  |
| 1 | Vvi-Vitvi12g00914\_t001 |  | \| \| |  |  |  |  |  |  |  |  |  |  |
| 1 | Vvi-Vitvi12g04297\_t001 |  | \| \| |  |  |  |  |  |  |  |  |  |  |
| 1 | Vvi-Vitvi12g00915\_t001 |  | \| \| |  |  |  |  |  |  |  |  |  |  |
| 1 | Vvi-Vitvi12g04298\_t001 |  | \| \| |  |  |  |  |  |  |  |  |  |  |
| 1 | Vvi-Vitvi12g04299\_t002 |  | Atr-ERN14613 |  |  |  |  |  |  |  |  |  |  |
| 1 | Vvi-Vitvi12g04300\_t002 |  | Atr-ERN14610 |  |  |  |  |  |  |  |  |  |  |
| 1 | Vvi-Vitvi12g02554\_t001 |  | Atr-ERN14609 |  |  |  |  |  |  |  |  |  |  |
| 0 | Vvi-Vitvi12g00918\_t001 |  |  |  |  |  |  |  |  |  |  |  |  |
| 0 | Vvi-Vitvi12g00919\_t001 |  |  |  |  |  |  |  |  |  |  |  |  |
| 0 | Vvi-Vitvi12g00921\_t001 |  |  |  |  |  |  |  |  |  |  |  |  |
| 0 | Vvi-Vitvi12g04301\_t001 |  |  |  |  |  |  |  |  |  |  |  |  |
| 0 | Vvi-Vitvi12g00927\_t001 |  |  |  |  |  |  |  |  |  |  |  |  |
| 0 | Vvi-Vitvi12g00928\_t001 |  |  |  |  |  |  |  |  |  |  |  |  |
| 0 | Vvi-Vitvi12g04302\_t001 |  |  |  |  |  |  |  |  |  |  |  |  |
| 0 | Vvi-Vitvi12g00941\_t001 |  |  |  |  |  |  |  |  |  |  |  |  |
| 0 | Vvi-Vitvi12g04303\_t001 |  |  |  |  |  |  |  |  |  |  |  |  |
| 0 | Vvi-Vitvi12g04304\_t001 |  |  |  |  |  |  |  |  |  |  |  |  |
| 0 | Vvi-Vitvi12g04305\_t001 |  |  |  |  |  |  |  |  |  |  |  |  |
| 0 | Vvi-Vitvi12g04306\_t001 |  |  |  |  |  |  |  |  |  |  |  |  |
| 0 | Vvi-Vitvi12g04307\_t001 |  |  |  |  |  |  |  |  |  |  |  |  |
| 0 | Vvi-Vitvi12g04308\_t001 |  |  |  |  |  |  |  |  |  |  |  |  |
| 0 | Vvi-Vitvi12g04309\_t001 |  |  |  |  |  |  |  |  |  |  |  |  |
| 0 | Vvi-Vitvi12g01053\_t001 |  |  |  |  |  |  |  |  |  |  |  |  |
| 0 | Vvi-Vitvi12g04310\_t001 |  |  |  |  |  |  |  |  |  |  |  |  |
| 0 | Vvi-Vitvi12g04311\_t001 |  |  |  |  |  |  |  |  |  |  |  |  |
| 0 | Vvi-Vitvi12g04312\_t001 |  |  |  |  |  |  |  |  |  |  |  |  |
| 0 | Vvi-Vitvi12g01102\_t001 |  |  |  |  |  |  |  |  |  |  |  |  |
| 0 | Vvi-Vitvi12g01115\_t001 |  |  |  |  |  |  |  |  |  |  |  |  |
| 0 | Vvi-Vitvi12g01121\_t001 |  |  |  |  |  |  |  |  |  |  |  |  |
| 0 | Vvi-Vitvi12g04313\_t001 |  |  |  |  |  |  |  |  |  |  |  |  |
| 0 | Vvi-Vitvi12g04314\_t001 |  |  |  |  |  |  |  |  |  |  |  |  |
| 0 | Vvi-Vitvi12g02556\_t001 |  |  |  |  |  |  |  |  |  |  |  |  |
| 0 | Vvi-Vitvi12g04315\_t001 |  |  |  |  |  |  |  |  |  |  |  |  |
| 0 | Vvi-Vitvi12g01181\_t001 |  |  |  |  |  |  |  |  |  |  |  |  |
| 0 | Vvi-Vitvi12g01199\_t001 |  |  |  |  |  |  |  |  |  |  |  |  |
| 0 | Vvi-Vitvi12g04316\_t001 |  |  |  |  |  |  |  |  |  |  |  |  |
| 0 | Vvi-Vitvi12g04317\_t001 |  |  |  |  |  |  |  |  |  |  |  |  |
| 0 | Vvi-Vitvi12g04318\_t001 |  |  |  |  |  |  |  |  |  |  |  |  |
| 0 | Vvi-Vitvi12g04319\_t001 |  |  |  |  |  |  |  |  |  |  |  |  |
| 0 | Vvi-Vitvi12g01241\_t001 |  |  |  |  |  |  |  |  |  |  |  |  |
| 0 | Vvi-Vitvi12g04320\_t001 |  |  |  |  |  |  |  |  |  |  |  |  |
| 0 | Vvi-Vitvi12g01264\_t001 |  |  |  |  |  |  |  |  |  |  |  |  |
| 0 | Vvi-Vitvi12g04321\_t001 |  |  |  |  |  |  |  |  |  |  |  |  |
| 0 | Vvi-Vitvi12g01270\_t001 |  |  |  |  |  |  |  |  |  |  |  |  |
| 0 | Vvi-Vitvi12g01272\_t001 |  |  |  |  |  |  |  |  |  |  |  |  |
| 0 | Vvi-Vitvi12g01277\_t001 |  |  |  |  |  |  |  |  |  |  |  |  |
| 0 | Vvi-Vitvi12g04322\_t001 |  |  |  |  |  |  |  |  |  |  |  |  |
| 0 | Vvi-Vitvi12g04323\_t001 |  |  |  |  |  |  |  |  |  |  |  |  |
| 0 | Vvi-Vitvi12g04324\_t001 |  |  |  |  |  |  |  |  |  |  |  |  |
| 0 | Vvi-Vitvi12g01323\_t001 |  |  |  |  |  |  |  |  |  |  |  |  |
| 0 | Vvi-Vitvi12g04325\_t001 |  |  |  |  |  |  |  |  |  |  |  |  |
| 0 | Vvi-Vitvi12g04326\_t001 |  |  |  |  |  |  |  |  |  |  |  |  |
| 0 | Vvi-Vitvi12g04327\_t001 |  |  |  |  |  |  |  |  |  |  |  |  |
| 0 | Vvi-Vitvi12g04328\_t001 |  |  |  |  |  |  |  |  |  |  |  |  |
| 0 | Vvi-Vitvi12g04329\_t001 |  |  |  |  |  |  |  |  |  |  |  |  |
| 0 | Vvi-Vitvi12g04330\_t001 |  |  |  |  |  |  |  |  |  |  |  |  |
| 0 | Vvi-Vitvi12g04331\_t001 |  |  |  |  |  |  |  |  |  |  |  |  |
| 0 | Vvi-Vitvi12g04332\_t001 |  |  |  |  |  |  |  |  |  |  |  |  |
| 0 | Vvi-Vitvi12g04333\_t001 |  |  |  |  |  |  |  |  |  |  |  |  |
| 0 | Vvi-Vitvi12g04334\_t001 |  |  |  |  |  |  |  |  |  |  |  |  |
| 0 | Vvi-Vitvi12g01349\_t001 |  |  |  |  |  |  |  |  |  |  |  |  |
| 0 | Vvi-Vitvi12g04335\_t001 |  |  |  |  |  |  |  |  |  |  |  |  |
| 0 | Vvi-Vitvi12g04336\_t001 |  |  |  |  |  |  |  |  |  |  |  |  |
| 0 | Vvi-Vitvi12g04337\_t001 |  |  |  |  |  |  |  |  |  |  |  |  |
| 0 | Vvi-Vitvi12g04338\_t001 |  |  |  |  |  |  |  |  |  |  |  |  |
| 0 | Vvi-Vitvi12g04339\_t001 |  |  |  |  |  |  |  |  |  |  |  |  |
| 0 | Vvi-Vitvi12g04340\_t001 |  |  |  |  |  |  |  |  |  |  |  |  |
| 0 | Vvi-Vitvi12g04341\_t001 |  |  |  |  |  |  |  |  |  |  |  |  |
| 0 | Vvi-Vitvi12g04342\_t001 |  |  |  |  |  |  |  |  |  |  |  |  |
| 0 | Vvi-Vitvi12g04343\_t001 |  |  |  |  |  |  |  |  |  |  |  |  |
| 0 | Vvi-Vitvi12g04344\_t001 |  |  |  |  |  |  |  |  |  |  |  |  |
| 0 | Vvi-Vitvi12g01431\_t001 |  |  |  |  |  |  |  |  |  |  |  |  |
| 0 | Vvi-Vitvi12g01449\_t001 |  |  |  |  |  |  |  |  |  |  |  |  |
| 0 | Vvi-Vitvi12g01467\_t002 |  |  |  |  |  |  |  |  |  |  |  |  |
| 1 | Vvi-Vitvi12g04345\_t001 |  | Atr-ERM94223 |  |  |  |  |  |  |  |  |  |  |
| 1 | Vvi-Vitvi12g01480\_t001 |  | \| \| |  |  |  |  |  |  |  |  |  |  |
| 1 | Vvi-Vitvi12g01497\_t001 |  | Atr-ERM94220 |  |  |  |  |  |  |  |  |  |  |
| 1 | Vvi-Vitvi12g04346\_t001 |  | \| \| |  |  |  |  |  |  |  |  |  |  |
| 1 | Vvi-Vitvi12g01529\_t001 |  | Atr-ERM94219 |  |  |  |  |  |  |  |  |  |  |
| 1 | Vvi-Vitvi12g01553\_t001 |  | \| \| |  |  |  |  |  |  |  |  |  |  |
| 1 | Vvi-Vitvi12g01555\_t001 |  | Atr-ERM94217 |  |  |  |  |  |  |  |  |  |  |
| 1 | Vvi-Vitvi12g01578\_t001 |  | \| \| |  |  |  |  |  |  |  |  |  |  |
| 1 | Vvi-Vitvi12g01584\_t001 |  | \| \| |  |  |  |  |  |  |  |  |  |  |
| 1 | Vvi-Vitvi12g04347\_t001 |  | Atr-ERM94209 |  |  |  |  |  |  |  |  |  |  |
| 1 | Vvi-Vitvi12g04348\_t001 |  | \| \| |  |  |  |  |  |  |  |  |  |  |
| 1 | Vvi-Vitvi12g04349\_t001 |  | \| \| |  |  |  |  |  |  |  |  |  |  |
| 1 | Vvi-Vitvi12g02559\_t001 |  | \| \| |  |  |  |  |  |  |  |  |  |  |
| 1 | Vvi-Vitvi12g04350\_t001 |  | \| \| |  |  |  |  |  |  |  |  |  |  |
| 1 | Vvi-Vitvi12g04351\_t001 |  | \| \| |  |  |  |  |  |  |  |  |  |  |
| 1 | Vvi-Vitvi12g04352\_t001 |  | \| \| |  |  |  |  |  |  |  |  |  |  |
| 1 | Vvi-Vitvi12g02561\_t001 |  | \| \| |  |  |  |  |  |  |  |  |  |  |
| 1 | Vvi-Vitvi12g01596\_t001 |  | \| \| |  |  |  |  |  |  |  |  |  |  |
| 1 | Vvi-Vitvi12g04353\_t001 |  | \| \| |  |  |  |  |  |  |  |  |  |  |
| 1 | Vvi-Vitvi12g01598\_t001 |  | \| \| |  |  |  |  |  |  |  |  |  |  |
| 1 | Vvi-Vitvi12g04354\_t001 |  | \| \| |  |  |  |  |  |  |  |  |  |  |
| 1 | Vvi-Vitvi12g01600\_t001 |  | \| \| |  |  |  |  |  |  |  |  |  |  |
| 1 | Vvi-Vitvi12g01601\_t001 |  | \| \| |  |  |  |  |  |  |  |  |  |  |
| 1 | Vvi-Vitvi12g01602\_t001 |  | \| \| |  |  |  |  |  |  |  |  |  |  |
| 1 | Vvi-Vitvi12g01603\_t001 |  | Atr-ERM94203 |  |  |  |  |  |  |  |  |  |  |
| 0 | Vvi-Vitvi12g01604\_t001 |  |  |  |  |  |  |  |  |  |  |  |  |
| 0 | Vvi-Vitvi12g04355\_t001 |  |  |  |  |  |  |  |  |  |  |  |  |
| 0 | Vvi-Vitvi12g01605\_t001 |  |  |  |  |  |  |  |  |  |  |  |  |
| 0 | Vvi-Vitvi12g04356\_t001 |  |  |  |  |  |  |  |  |  |  |  |  |
| 0 | Vvi-Vitvi12g04357\_t001 |  |  |  |  |  |  |  |  |  |  |  |  |
| 0 | Vvi-Vitvi12g01610\_t001 |  |  |  |  |  |  |  |  |  |  |  |  |
| 0 | Vvi-Vitvi12g01611\_t001 |  |  |  |  |  |  |  |  |  |  |  |  |
| 0 | Vvi-Vitvi12g04358\_t001 |  |  |  |  |  |  |  |  |  |  |  |  |
| 0 | Vvi-Vitvi12g01613\_t001 |  |  |  |  |  |  |  |  |  |  |  |  |
| 0 | Vvi-Vitvi12g04359\_t001 |  |  |  |  |  |  |  |  |  |  |  |  |
| 0 | Vvi-Vitvi12g04360\_t001 |  |  |  |  |  |  |  |  |  |  |  |  |
| 0 | Vvi-Vitvi12g01615\_t001 |  |  |  |  |  |  |  |  |  |  |  |  |
| 0 | Vvi-Vitvi12g01616\_t001 |  |  |  |  |  |  |  |  |  |  |  |  |
| 0 | Vvi-Vitvi12g01617\_t001 |  |  |  |  |  |  |  |  |  |  |  |  |
| 0 | Vvi-Vitvi12g01618\_t002 |  |  |  |  |  |  |  |  |  |  |  |  |
| 0 | Vvi-Vitvi12g01620\_t001 |  |  |  |  |  |  |  |  |  |  |  |  |
| 0 | Vvi-Vitvi12g01622\_t001 |  |  |  |  |  |  |  |  |  |  |  |  |
| 0 | Vvi-Vitvi12g01624\_t001 |  |  |  |  |  |  |  |  |  |  |  |  |
| 0 | Vvi-Vitvi12g02565\_t001 |  |  |  |  |  |  |  |  |  |  |  |  |
| 0 | Vvi-Vitvi12g01626\_t001 |  |  |  |  |  |  |  |  |  |  |  |  |
| 0 | Vvi-Vitvi12g01627\_t001 |  |  |  |  |  |  |  |  |  |  |  |  |
| 0 | Vvi-Vitvi12g04361\_t001 |  |  |  |  |  |  |  |  |  |  |  |  |
| 0 | Vvi-Vitvi12g04362\_t001 |  |  |  |  |  |  |  |  |  |  |  |  |
| 0 | Vvi-Vitvi12g01629\_t001 |  |  |  |  |  |  |  |  |  |  |  |  |
| 0 | Vvi-Vitvi12g02568\_t001 |  |  |  |  |  |  |  |  |  |  |  |  |
| 0 | Vvi-Vitvi12g01636\_t001 |  |  |  |  |  |  |  |  |  |  |  |  |
| 0 | Vvi-Vitvi12g01638\_t001 |  |  |  |  |  |  |  |  |  |  |  |  |
| 0 | Vvi-Vitvi12g04363\_t003 |  |  |  |  |  |  |  |  |  |  |  |  |
| 0 | Vvi-Vitvi12g01641\_t001 |  |  |  |  |  |  |  |  |  |  |  |  |
| 0 | Vvi-Vitvi12g04364\_t001 |  |  |  |  |  |  |  |  |  |  |  |  |
| 0 | Vvi-Vitvi12g04365\_t001 |  |  |  |  |  |  |  |  |  |  |  |  |
| 0 | Vvi-Vitvi12g04366\_t001 |  |  |  |  |  |  |  |  |  |  |  |  |
| 0 | Vvi-Vitvi12g04367\_t001 |  |  |  |  |  |  |  |  |  |  |  |  |
| 0 | Vvi-Vitvi12g04368\_t001 |  |  |  |  |  |  |  |  |  |  |  |  |
| 0 | Vvi-Vitvi12g01643\_t001 |  |  |  |  |  |  |  |  |  |  |  |  |
| 0 | Vvi-Vitvi12g04369\_t001 |  |  |  |  |  |  |  |  |  |  |  |  |
| 0 | Vvi-Vitvi12g04370\_t001 |  |  |  |  |  |  |  |  |  |  |  |  |
| 0 | Vvi-Vitvi12g04371\_t001 |  |  |  |  |  |  |  |  |  |  |  |  |
| 0 | Vvi-Vitvi12g02578\_t001 |  |  |  |  |  |  |  |  |  |  |  |  |
| 0 | Vvi-Vitvi12g04372\_t001 |  |  |  |  |  |  |  |  |  |  |  |  |
| 0 | Vvi-Vitvi12g01651\_t001 |  |  |  |  |  |  |  |  |  |  |  |  |
| 0 | Vvi-Vitvi12g04373\_t001 |  |  |  |  |  |  |  |  |  |  |  |  |
| 0 | Vvi-Vitvi12g04374\_t001 |  |  |  |  |  |  |  |  |  |  |  |  |
| 0 | Vvi-Vitvi12g01652\_t001 |  |  |  |  |  |  |  |  |  |  |  |  |
| 0 | Vvi-Vitvi12g01655\_t001 |  |  |  |  |  |  |  |  |  |  |  |  |
| 0 | Vvi-Vitvi12g01656\_t001 |  |  |  |  |  |  |  |  |  |  |  |  |
| 0 | Vvi-Vitvi12g01657\_t001 |  |  |  |  |  |  |  |  |  |  |  |  |
| 0 | Vvi-Vitvi12g01658\_t001 |  |  |  |  |  |  |  |  |  |  |  |  |
| 0 | Vvi-Vitvi12g04375\_t001 |  |  |  |  |  |  |  |  |  |  |  |  |
| 0 | Vvi-Vitvi12g04376\_t001 |  |  |  |  |  |  |  |  |  |  |  |  |
| 0 | Vvi-Vitvi12g01664\_t001 |  |  |  |  |  |  |  |  |  |  |  |  |
| 0 | Vvi-Vitvi12g01665\_t001 |  |  |  |  |  |  |  |  |  |  |  |  |
| 0 | Vvi-Vitvi12g01666\_t002 |  |  |  |  |  |  |  |  |  |  |  |  |
| 0 | Vvi-Vitvi12g01667\_t001 |  |  |  |  |  |  |  |  |  |  |  |  |
| 0 | Vvi-Vitvi12g04377\_t001 |  |  |  |  |  |  |  |  |  |  |  |  |
| 0 | Vvi-Vitvi12g01670\_t001 |  |  |  |  |  |  |  |  |  |  |  |  |
| 0 | Vvi-Vitvi12g02584\_t001 |  |  |  |  |  |  |  |  |  |  |  |  |
| 0 | Vvi-Vitvi12g01673\_t001 |  |  |  |  |  |  |  |  |  |  |  |  |
| 0 | Vvi-Vitvi12g01674\_t001 |  |  |  |  |  |  |  |  |  |  |  |  |
| 0 | Vvi-Vitvi12g01676\_t001 |  |  |  |  |  |  |  |  |  |  |  |  |
| 0 | Vvi-Vitvi12g04378\_t001 |  |  |  |  |  |  |  |  |  |  |  |  |
| 0 | Vvi-Vitvi12g01677\_t001 |  |  |  |  |  |  |  |  |  |  |  |  |
| 0 | Vvi-Vitvi12g04379\_t001 |  |  |  |  |  |  |  |  |  |  |  |  |
| 0 | Vvi-Vitvi12g04380\_t001 |  |  |  |  |  |  |  |  |  |  |  |  |
| 0 | Vvi-Vitvi12g04381\_t001 |  |  |  |  |  |  |  |  |  |  |  |  |
| 0 | Vvi-Vitvi12g04382\_t001 |  |  |  |  |  |  |  |  |  |  |  |  |
| 0 | Vvi-Vitvi12g04383\_t001 |  |  |  |  |  |  |  |  |  |  |  |  |
| 0 | Vvi-Vitvi12g04384\_t001 |  |  |  |  |  |  |  |  |  |  |  |  |
| 0 | Vvi-Vitvi12g04385\_t001 |  |  |  |  |  |  |  |  |  |  |  |  |
| 0 | Vvi-Vitvi12g01695\_t001 |  |  |  |  |  |  |  |  |  |  |  |  |
| 0 | Vvi-Vitvi12g04386\_t001 |  |  |  |  |  |  |  |  |  |  |  |  |
| 0 | Vvi-Vitvi12g04387\_t001 |  |  |  |  |  |  |  |  |  |  |  |  |
| 0 | Vvi-Vitvi12g04388\_t001 |  |  |  |  |  |  |  |  |  |  |  |  |
| 0 | Vvi-Vitvi12g01703\_t001 |  |  |  |  |  |  |  |  |  |  |  |  |
| 0 | Vvi-Vitvi12g04389\_t001 |  |  |  |  |  |  |  |  |  |  |  |  |
| 0 | Vvi-Vitvi12g04390\_t001 |  |  |  |  |  |  |  |  |  |  |  |  |
| 0 | Vvi-Vitvi12g02592\_t001 |  |  |  |  |  |  |  |  |  |  |  |  |
| 0 | Vvi-Vitvi12g04391\_t001 |  |  |  |  |  |  |  |  |  |  |  |  |
| 0 | Vvi-Vitvi12g01709\_t001 |  |  |  |  |  |  |  |  |  |  |  |  |
| 0 | Vvi-Vitvi12g04392\_t001 |  |  |  |  |  |  |  |  |  |  |  |  |
| 0 | Vvi-Vitvi12g04393\_t001 |  |  |  |  |  |  |  |  |  |  |  |  |
| 0 | Vvi-Vitvi12g04394\_t001 |  |  |  |  |  |  |  |  |  |  |  |  |
| 0 | Vvi-Vitvi12g04395\_t001 |  |  |  |  |  |  |  |  |  |  |  |  |
| 0 | Vvi-Vitvi12g01722\_t001 |  |  |  |  |  |  |  |  |  |  |  |  |
| 0 | Vvi-Vitvi12g04396\_t001 |  |  |  |  |  |  |  |  |  |  |  |  |
| 0 | Vvi-Vitvi12g04397\_t001 |  |  |  |  |  |  |  |  |  |  |  |  |
| 0 | Vvi-Vitvi12g01723\_t001 |  |  |  |  |  |  |  |  |  |  |  |  |
| 0 | Vvi-Vitvi12g04398\_t001 |  |  |  |  |  |  |  |  |  |  |  |  |
| 0 | Vvi-Vitvi12g04399\_t001 |  |  |  |  |  |  |  |  |  |  |  |  |
| 0 | Vvi-Vitvi12g01738\_t001 |  |  |  |  |  |  |  |  |  |  |  |  |
| 0 | Vvi-Vitvi12g04400\_t001 |  |  |  |  |  |  |  |  |  |  |  |  |
| 0 | Vvi-Vitvi12g01740\_t001 |  |  |  |  |  |  |  |  |  |  |  |  |
| 0 | Vvi-Vitvi12g04401\_t001 |  |  |  |  |  |  |  |  |  |  |  |  |
| 0 | Vvi-Vitvi12g01741\_t001 |  |  |  |  |  |  |  |  |  |  |  |  |
| 0 | Vvi-Vitvi12g04402\_t001 |  |  |  |  |  |  |  |  |  |  |  |  |
| 0 | Vvi-Vitvi12g02602\_t001 |  |  |  |  |  |  |  |  |  |  |  |  |
| 0 | Vvi-Vitvi12g04403\_t001 |  |  |  |  |  |  |  |  |  |  |  |  |
| 0 | Vvi-Vitvi12g04404\_t001 |  |  |  |  |  |  |  |  |  |  |  |  |
| 0 | Vvi-Vitvi12g04405\_t001 |  |  |  |  |  |  |  |  |  |  |  |  |
| 0 | Vvi-Vitvi12g01747\_t001 |  |  |  |  |  |  |  |  |  |  |  |  |
| 0 | Vvi-Vitvi12g04406\_t001 |  |  |  |  |  |  |  |  |  |  |  |  |
| 0 | Vvi-Vitvi12g04407\_t001 |  |  |  |  |  |  |  |  |  |  |  |  |
| 0 | Vvi-Vitvi12g04408\_t001 |  |  |  |  |  |  |  |  |  |  |  |  |
| 0 | Vvi-Vitvi12g04409\_t001 |  |  |  |  |  |  |  |  |  |  |  |  |
| 0 | Vvi-Vitvi12g01750\_t001 |  |  |  |  |  |  |  |  |  |  |  |  |
| 0 | Vvi-Vitvi12g01751\_t001 |  |  |  |  |  |  |  |  |  |  |  |  |
| 0 | Vvi-Vitvi12g01752\_t001 |  |  |  |  |  |  |  |  |  |  |  |  |
| 0 | Vvi-Vitvi12g04410\_t001 |  |  |  |  |  |  |  |  |  |  |  |  |
| 0 | Vvi-Vitvi12g02607\_t001 |  |  |  |  |  |  |  |  |  |  |  |  |
| 0 | Vvi-Vitvi12g01756\_t001 |  |  |  |  |  |  |  |  |  |  |  |  |
| 0 | Vvi-Vitvi12g04411\_t001 |  |  |  |  |  |  |  |  |  |  |  |  |
| 0 | Vvi-Vitvi12g04412\_t001 |  |  |  |  |  |  |  |  |  |  |  |  |
| 0 | Vvi-Vitvi12g01758\_t001 |  |  |  |  |  |  |  |  |  |  |  |  |
| 0 | Vvi-Vitvi12g01759\_t001 |  |  |  |  |  |  |  |  |  |  |  |  |
| 0 | Vvi-Vitvi12g01760\_t001 |  |  |  |  |  |  |  |  |  |  |  |  |
| 0 | Vvi-Vitvi12g04413\_t001 |  |  |  |  |  |  |  |  |  |  |  |  |
| 0 | Vvi-Vitvi12g04414\_t001 |  |  |  |  |  |  |  |  |  |  |  |  |
| 0 | Vvi-Vitvi12g04415\_t001 |  |  |  |  |  |  |  |  |  |  |  |  |
| 0 | Vvi-Vitvi12g01764\_t001 |  |  |  |  |  |  |  |  |  |  |  |  |
| 0 | Vvi-Vitvi12g04416\_t001 |  |  |  |  |  |  |  |  |  |  |  |  |
| 0 | Vvi-Vitvi12g04417\_t001 |  |  |  |  |  |  |  |  |  |  |  |  |
| 0 | Vvi-Vitvi12g01776\_t001 |  |  |  |  |  |  |  |  |  |  |  |  |
| 0 | Vvi-Vitvi12g01780\_t001 |  |  |  |  |  |  |  |  |  |  |  |  |
| 0 | Vvi-Vitvi12g02621\_t001 |  |  |  |  |  |  |  |  |  |  |  |  |
| 0 | Vvi-Vitvi12g04419\_t001 |  |  |  |  |  |  |  |  |  |  |  |  |
| 0 | Vvi-Vitvi12g04420\_t001 |  |  |  |  |  |  |  |  |  |  |  |  |
| 0 | Vvi-Vitvi12g02623\_t001 |  |  |  |  |  |  |  |  |  |  |  |  |
| 0 | Vvi-Vitvi12g04421\_t001 |  |  |  |  |  |  |  |  |  |  |  |  |
| 0 | Vvi-Vitvi12g04422\_t001 |  |  |  |  |  |  |  |  |  |  |  |  |
| 0 | Vvi-Vitvi12g04423\_t001 |  |  |  |  |  |  |  |  |  |  |  |  |
| 0 | Vvi-Vitvi12g04424\_t001 |  |  |  |  |  |  |  |  |  |  |  |  |
| 0 | Vvi-Vitvi12g02630\_t001 |  |  |  |  |  |  |  |  |  |  |  |  |
| 0 | Vvi-Vitvi12g01790\_t001 |  |  |  |  |  |  |  |  |  |  |  |  |
| 0 | Vvi-Vitvi12g01792\_t002 |  |  |  |  |  |  |  |  |  |  |  |  |
| 0 | Vvi-Vitvi12g01793\_t001 |  |  |  |  |  |  |  |  |  |  |  |  |
| 0 | Vvi-Vitvi12g02633\_t001 |  |  |  |  |  |  |  |  |  |  |  |  |
| 0 | Vvi-Vitvi12g04425\_t001 |  |  |  |  |  |  |  |  |  |  |  |  |
| 0 | Vvi-Vitvi12g01795\_t001 |  |  |  |  |  |  |  |  |  |  |  |  |
| 0 | Vvi-Vitvi12g01796\_t001 |  |  |  |  |  |  |  |  |  |  |  |  |
| 0 | Vvi-Vitvi12g01799\_t001 |  |  |  |  |  |  |  |  |  |  |  |  |
| 0 | Vvi-Vitvi12g01800\_t001 |  |  |  |  |  |  |  |  |  |  |  |  |
| 0 | Vvi-Vitvi12g01801\_t002 |  |  |  |  |  |  |  |  |  |  |  |  |
| 0 | Vvi-Vitvi12g04426\_t001 |  |  |  |  |  |  |  |  |  |  |  |  |
| 0 | Vvi-Vitvi12g01802\_t001 |  |  |  |  |  |  |  |  |  |  |  |  |
| 0 | Vvi-Vitvi12g04427\_t001 |  |  |  |  |  |  |  |  |  |  |  |  |
| 0 | Vvi-Vitvi12g01804\_t001 |  |  |  |  |  |  |  |  |  |  |  |  |
| 0 | Vvi-Vitvi12g04428\_t001 |  |  |  |  |  |  |  |  |  |  |  |  |
| 0 | Vvi-Vitvi12g01806\_t001 |  |  |  |  |  |  |  |  |  |  |  |  |
| 0 | Vvi-Vitvi12g01808\_t001 |  |  |  |  |  |  |  |  |  |  |  |  |
| 0 | Vvi-Vitvi12g01809\_t003 |  |  |  |  |  |  |  |  |  |  |  |  |
| 0 | Vvi-Vitvi12g01810\_t001 |  |  |  |  |  |  |  |  |  |  |  |  |
| 0 | Vvi-Vitvi12g01812\_t001 |  |  |  |  |  |  |  |  |  |  |  |  |
| 0 | Vvi-Vitvi12g01813\_t001 |  |  |  |  |  |  |  |  |  |  |  |  |
| 0 | Vvi-Vitvi12g01814\_t001 |  |  |  |  |  |  |  |  |  |  |  |  |
| 0 | Vvi-Vitvi12g01815\_t001 |  |  |  |  |  |  |  |  |  |  |  |  |
| 0 | Vvi-Vitvi12g01816\_t001 |  |  |  |  |  |  |  |  |  |  |  |  |
| 0 | Vvi-Vitvi12g01818\_t001 |  |  |  |  |  |  |  |  |  |  |  |  |
| 0 | Vvi-Vitvi12g04429\_t001 |  |  |  |  |  |  |  |  |  |  |  |  |
| 0 | Vvi-Vitvi12g01819\_t001 |  |  |  |  |  |  |  |  |  |  |  |  |
| 0 | Vvi-Vitvi12g01820\_t001 |  |  |  |  |  |  |  |  |  |  |  |  |
| 0 | Vvi-Vitvi12g01822\_t001 |  |  |  |  |  |  |  |  |  |  |  |  |
| 0 | Vvi-Vitvi12g02638\_t001 |  |  |  |  |  |  |  |  |  |  |  |  |
| 0 | Vvi-Vitvi12g04430\_t001 |  |  |  |  |  |  |  |  |  |  |  |  |
| 0 | Vvi-Vitvi12g04431\_t001 |  |  |  |  |  |  |  |  |  |  |  |  |
| 0 | Vvi-Vitvi12g04432\_t001 |  |  |  |  |  |  |  |  |  |  |  |  |
| 0 | Vvi-Vitvi12g04433\_t001 |  |  |  |  |  |  |  |  |  |  |  |  |
| 0 | Vvi-Vitvi12g04434\_t001 |  |  |  |  |  |  |  |  |  |  |  |  |
| 0 | Vvi-Vitvi12g04435\_t001 |  |  |  |  |  |  |  |  |  |  |  |  |
| 0 | Vvi-Vitvi12g02640\_t001 |  |  |  |  |  |  |  |  |  |  |  |  |
| 0 | Vvi-Vitvi12g02641\_t001 |  |  |  |  |  |  |  |  |  |  |  |  |
| 0 | Vvi-Vitvi12g04436\_t001 |  |  |  |  |  |  |  |  |  |  |  |  |
| 0 | Vvi-Vitvi12g04437\_t001 |  |  |  |  |  |  |  |  |  |  |  |  |
| 0 | Vvi-Vitvi12g02642\_t001 |  |  |  |  |  |  |  |  |  |  |  |  |
| 0 | Vvi-Vitvi12g04438\_t001 |  |  |  |  |  |  |  |  |  |  |  |  |
| 0 | Vvi-Vitvi12g04439\_t001 |  |  |  |  |  |  |  |  |  |  |  |  |
| 0 | Vvi-Vitvi12g01831\_t002 |  |  |  |  |  |  |  |  |  |  |  |  |
| 0 | Vvi-Vitvi12g04440\_t001 |  |  |  |  |  |  |  |  |  |  |  |  |
| 0 | Vvi-Vitvi12g04441\_t001 |  |  |  |  |  |  |  |  |  |  |  |  |
| 0 | Vvi-Vitvi12g01836\_t001 |  |  |  |  |  |  |  |  |  |  |  |  |
| 0 | Vvi-Vitvi12g01838\_t001 |  |  |  |  |  |  |  |  |  |  |  |  |
| 0 | Vvi-Vitvi12g01839\_t001 |  |  |  |  |  |  |  |  |  |  |  |  |
| 0 | Vvi-Vitvi12g01840\_t001 |  |  |  |  |  |  |  |  |  |  |  |  |
| 0 | Vvi-Vitvi12g01841\_t001 |  |  |  |  |  |  |  |  |  |  |  |  |
| 0 | Vvi-Vitvi12g01842\_t001 |  |  |  |  |  |  |  |  |  |  |  |  |
| 0 | Vvi-Vitvi12g04442\_t001 |  |  |  |  |  |  |  |  |  |  |  |  |
| 0 | Vvi-Vitvi12g01844\_t001 |  |  |  |  |  |  |  |  |  |  |  |  |
| 0 | Vvi-Vitvi12g04443\_t001 |  |  |  |  |  |  |  |  |  |  |  |  |
| 0 | Vvi-Vitvi12g04444\_t001 |  |  |  |  |  |  |  |  |  |  |  |  |
| 0 | Vvi-Vitvi12g01846\_t001 |  |  |  |  |  |  |  |  |  |  |  |  |
| 0 | Vvi-Vitvi12g04445\_t001 |  |  |  |  |  |  |  |  |  |  |  |  |
| 0 | Vvi-Vitvi12g04446\_t001 |  |  |  |  |  |  |  |  |  |  |  |  |
| 0 | Vvi-Vitvi12g04447\_t001 |  |  |  |  |  |  |  |  |  |  |  |  |
| 0 | Vvi-Vitvi12g04448\_t001 |  |  |  |  |  |  |  |  |  |  |  |  |
| 0 | Vvi-Vitvi12g02646\_t001 |  |  |  |  |  |  |  |  |  |  |  |  |
| 0 | Vvi-Vitvi12g04449\_t001 |  |  |  |  |  |  |  |  |  |  |  |  |
| 0 | Vvi-Vitvi12g01849\_t001 |  |  |  |  |  |  |  |  |  |  |  |  |
| 0 | Vvi-Vitvi12g02649\_t001 |  |  |  |  |  |  |  |  |  |  |  |  |
| 0 | Vvi-Vitvi12g04450\_t001 |  |  |  |  |  |  |  |  |  |  |  |  |
| 0 | Vvi-Vitvi12g04451\_t001 |  |  |  |  |  |  |  |  |  |  |  |  |
| 0 | Vvi-Vitvi12g04452\_t001 |  |  |  |  |  |  |  |  |  |  |  |  |
| 0 | Vvi-Vitvi12g04453\_t001 |  |  |  |  |  |  |  |  |  |  |  |  |
| 0 | Vvi-Vitvi12g04454\_t001 |  |  |  |  |  |  |  |  |  |  |  |  |
| 0 | Vvi-Vitvi12g04455\_t001 |  |  |  |  |  |  |  |  |  |  |  |  |
| 0 | Vvi-Vitvi12g04456\_t001 |  |  |  |  |  |  |  |  |  |  |  |  |
| 0 | Vvi-Vitvi12g04457\_t001 |  |  |  |  |  |  |  |  |  |  |  |  |
| 0 | Vvi-Vitvi12g04458\_t001 |  |  |  |  |  |  |  |  |  |  |  |  |
| 0 | Vvi-Vitvi12g04459\_t001 |  |  |  |  |  |  |  |  |  |  |  |  |
| 0 | Vvi-Vitvi12g04460\_t001 |  |  |  |  |  |  |  |  |  |  |  |  |
| 0 | Vvi-Vitvi12g01857\_t001 |  |  |  |  |  |  |  |  |  |  |  |  |
| 0 | Vvi-Vitvi12g04461\_t001 |  |  |  |  |  |  |  |  |  |  |  |  |
| 0 | Vvi-Vitvi12g04462\_t001 |  |  |  |  |  |  |  |  |  |  |  |  |
| 0 | Vvi-Vitvi12g04463\_t001 |  |  |  |  |  |  |  |  |  |  |  |  |
| 0 | Vvi-Vitvi12g04464\_t001 |  |  |  |  |  |  |  |  |  |  |  |  |
| 0 | Vvi-Vitvi12g04465\_t001 |  |  |  |  |  |  |  |  |  |  |  |  |
| 0 | Vvi-Vitvi12g04466\_t001 |  |  |  |  |  |  |  |  |  |  |  |  |
| 0 | Vvi-Vitvi12g04467\_t001 |  |  |  |  |  |  |  |  |  |  |  |  |
| 0 | Vvi-Vitvi12g04468\_t001 |  |  |  |  |  |  |  |  |  |  |  |  |
| 0 | Vvi-Vitvi12g04469\_t001 |  |  |  |  |  |  |  |  |  |  |  |  |
| 0 | Vvi-Vitvi12g04470\_t001 |  |  |  |  |  |  |  |  |  |  |  |  |
| 0 | Vvi-Vitvi12g04471\_t001 |  |  |  |  |  |  |  |  |  |  |  |  |
| 0 | Vvi-Vitvi12g04472\_t001 |  |  |  |  |  |  |  |  |  |  |  |  |
| 0 | Vvi-Vitvi12g04473\_t001 |  |  |  |  |  |  |  |  |  |  |  |  |
| 0 | Vvi-Vitvi12g04474\_t001 |  |  |  |  |  |  |  |  |  |  |  |  |
| 0 | Vvi-Vitvi12g04475\_t001 |  |  |  |  |  |  |  |  |  |  |  |  |
| 0 | Vvi-Vitvi12g04476\_t001 |  |  |  |  |  |  |  |  |  |  |  |  |
| 0 | Vvi-Vitvi12g04477\_t001 |  |  |  |  |  |  |  |  |  |  |  |  |
| 0 | Vvi-Vitvi12g01864\_t001 |  |  |  |  |  |  |  |  |  |  |  |  |
| 0 | Vvi-Vitvi12g02654\_t001 |  |  |  |  |  |  |  |  |  |  |  |  |
| 0 | Vvi-Vitvi12g01865\_t001 |  |  |  |  |  |  |  |  |  |  |  |  |
| 0 | Vvi-Vitvi12g04478\_t001 |  |  |  |  |  |  |  |  |  |  |  |  |
| 0 | Vvi-Vitvi12g04479\_t001 |  |  |  |  |  |  |  |  |  |  |  |  |
| 0 | Vvi-Vitvi12g01866\_t002 |  |  |  |  |  |  |  |  |  |  |  |  |
| 0 | Vvi-Vitvi12g04480\_t001 |  |  |  |  |  |  |  |  |  |  |  |  |
| 0 | Vvi-Vitvi12g01867\_t001 |  |  |  |  |  |  |  |  |  |  |  |  |
| 0 | Vvi-Vitvi12g01868\_t001 |  |  |  |  |  |  |  |  |  |  |  |  |
| 0 | Vvi-Vitvi12g01869\_t001 |  |  |  |  |  |  |  |  |  |  |  |  |
| 0 | Vvi-Vitvi12g01870\_t001 |  |  |  |  |  |  |  |  |  |  |  |  |
| 0 | Vvi-Vitvi12g01871\_t001 |  |  |  |  |  |  |  |  |  |  |  |  |
| 0 | Vvi-Vitvi12g01873\_t001 |  |  |  |  |  |  |  |  |  |  |  |  |
| 0 | Vvi-Vitvi12g01876\_t002 |  |  |  |  |  |  |  |  |  |  |  |  |
| 0 | Vvi-Vitvi12g04481\_t001 |  |  |  |  |  |  |  |  |  |  |  |  |
| 0 | Vvi-Vitvi12g02659\_t001 |  |  |  |  |  |  |  |  |  |  |  |  |
| 0 | Vvi-Vitvi12g01880\_t001 |  |  |  |  |  |  |  |  |  |  |  |  |
| 0 | Vvi-Vitvi12g01881\_t001 |  |  |  |  |  |  |  |  |  |  |  |  |
| 0 | Vvi-Vitvi12g01882\_t001 |  |  |  |  |  |  |  |  |  |  |  |  |
| 0 | Vvi-Vitvi12g01883\_t001 |  |  |  |  |  |  |  |  |  |  |  |  |
| 0 | Vvi-Vitvi12g01884\_t001 |  |  |  |  |  |  |  |  |  |  |  |  |
| 0 | Vvi-Vitvi12g04482\_t001 |  |  |  |  |  |  |  |  |  |  |  |  |
| 0 | Vvi-Vitvi12g01885\_t001 |  |  |  |  |  |  |  |  |  |  |  |  |
| 0 | Vvi-Vitvi12g04483\_t001 |  |  |  |  |  |  |  |  |  |  |  |  |
| 0 | Vvi-Vitvi12g02663\_t001 |  |  |  |  |  |  |  |  |  |  |  |  |
| 0 | Vvi-Vitvi12g04485\_t001 |  |  |  |  |  |  |  |  |  |  |  |  |
| 0 | Vvi-Vitvi12g04486\_t001 |  |  |  |  |  |  |  |  |  |  |  |  |
| 0 | Vvi-Vitvi12g04487\_t001 |  |  |  |  |  |  |  |  |  |  |  |  |
| 0 | Vvi-Vitvi12g04488\_t001 |  |  |  |  |  |  |  |  |  |  |  |  |
| 0 | Vvi-Vitvi12g04489\_t001 |  |  |  |  |  |  |  |  |  |  |  |  |
| 0 | Vvi-Vitvi12g04490\_t001 |  |  |  |  |  |  |  |  |  |  |  |  |
| 0 | Vvi-Vitvi12g04491\_t001 |  |  |  |  |  |  |  |  |  |  |  |  |
| 0 | Vvi-Vitvi12g01894\_t001 |  |  |  |  |  |  |  |  |  |  |  |  |
| 0 | Vvi-Vitvi12g01896\_t001 |  |  |  |  |  |  |  |  |  |  |  |  |
| 0 | Vvi-Vitvi12g04492\_t001 |  |  |  |  |  |  |  |  |  |  |  |  |
| 0 | Vvi-Vitvi12g01897\_t001 |  |  |  |  |  |  |  |  |  |  |  |  |
| 0 | Vvi-Vitvi12g04493\_t001 |  |  |  |  |  |  |  |  |  |  |  |  |
| 0 | Vvi-Vitvi12g01899\_t001 |  |  |  |  |  |  |  |  |  |  |  |  |
| 0 | Vvi-Vitvi12g01900\_t001 |  |  |  |  |  |  |  |  |  |  |  |  |
| 0 | Vvi-Vitvi12g01901\_t001 |  |  |  |  |  |  |  |  |  |  |  |  |
| 0 | Vvi-Vitvi12g01902\_t001 |  |  |  |  |  |  |  |  |  |  |  |  |
| 0 | Vvi-Vitvi12g04494\_t001 |  |  |  |  |  |  |  |  |  |  |  |  |
| 0 | Vvi-Vitvi12g01903\_t001 |  |  |  |  |  |  |  |  |  |  |  |  |
| 0 | Vvi-Vitvi12g01904\_t001 |  |  |  |  |  |  |  |  |  |  |  |  |
| 0 | Vvi-Vitvi12g01906\_t001 |  |  |  |  |  |  |  |  |  |  |  |  |
| 0 | Vvi-Vitvi12g04495\_t001 |  |  |  |  |  |  |  |  |  |  |  |  |
| 0 | Vvi-Vitvi12g04496\_t001 |  |  |  |  |  |  |  |  |  |  |  |  |
| 0 | Vvi-Vitvi12g04497\_t001 |  |  |  |  |  |  |  |  |  |  |  |  |
| 0 | Vvi-Vitvi12g04498\_t001 |  |  |  |  |  |  |  |  |  |  |  |  |
| 0 | Vvi-Vitvi12g04499\_t001 |  |  |  |  |  |  |  |  |  |  |  |  |
| 0 | Vvi-Vitvi12g04500\_t001 |  |  |  |  |  |  |  |  |  |  |  |  |
| 0 | Vvi-Vitvi12g04501\_t001 |  |  |  |  |  |  |  |  |  |  |  |  |
| 0 | Vvi-Vitvi12g01910\_t001 |  |  |  |  |  |  |  |  |  |  |  |  |
| 0 | Vvi-Vitvi12g04502\_t001 |  |  |  |  |  |  |  |  |  |  |  |  |
| 0 | Vvi-Vitvi12g04503\_t001 |  |  |  |  |  |  |  |  |  |  |  |  |
| 0 | Vvi-Vitvi12g04504\_t001 |  |  |  |  |  |  |  |  |  |  |  |  |
| 0 | Vvi-Vitvi12g04505\_t001 |  |  |  |  |  |  |  |  |  |  |  |  |
| 0 | Vvi-Vitvi12g01912\_t001 |  |  |  |  |  |  |  |  |  |  |  |  |
| 0 | Vvi-Vitvi12g04506\_t001 |  |  |  |  |  |  |  |  |  |  |  |  |
| 0 | Vvi-Vitvi12g01913\_t001 |  |  |  |  |  |  |  |  |  |  |  |  |
| 0 | Vvi-Vitvi12g01914\_t001 |  |  |  |  |  |  |  |  |  |  |  |  |
| 0 | Vvi-Vitvi12g01915\_t001 |  |  |  |  |  |  |  |  |  |  |  |  |
| 0 | Vvi-Vitvi12g04507\_t001 |  |  |  |  |  |  |  |  |  |  |  |  |
| 0 | Vvi-Vitvi12g04508\_t001 |  |  |  |  |  |  |  |  |  |  |  |  |
| 0 | Vvi-Vitvi12g04509\_t001 |  |  |  |  |  |  |  |  |  |  |  |  |
| 0 | Vvi-Vitvi12g01916\_t001 |  |  |  |  |  |  |  |  |  |  |  |  |
| 0 | Vvi-Vitvi12g04510\_t001 |  |  |  |  |  |  |  |  |  |  |  |  |
| 0 | Vvi-Vitvi12g04511\_t001 |  |  |  |  |  |  |  |  |  |  |  |  |
| 0 | Vvi-Vitvi12g04512\_t001 |  |  |  |  |  |  |  |  |  |  |  |  |
| 0 | Vvi-Vitvi12g04513\_t001 |  |  |  |  |  |  |  |  |  |  |  |  |
| 0 | Vvi-Vitvi12g02672\_t001 |  |  |  |  |  |  |  |  |  |  |  |  |
| 0 | Vvi-Vitvi12g02675\_t001 |  |  |  |  |  |  |  |  |  |  |  |  |
| 0 | Vvi-Vitvi12g04514\_t001 |  |  |  |  |  |  |  |  |  |  |  |  |
| 0 | Vvi-Vitvi12g04515\_t001 |  |  |  |  |  |  |  |  |  |  |  |  |
| 0 | Vvi-Vitvi12g04516\_t001 |  |  |  |  |  |  |  |  |  |  |  |  |
| 0 | Vvi-Vitvi12g04517\_t001 |  |  |  |  |  |  |  |  |  |  |  |  |
| 0 | Vvi-Vitvi12g04518\_t001 |  |  |  |  |  |  |  |  |  |  |  |  |
| 0 | Vvi-Vitvi12g02676\_t001 |  |  |  |  |  |  |  |  |  |  |  |  |
| 0 | Vvi-Vitvi12g04519\_t001 |  |  |  |  |  |  |  |  |  |  |  |  |
| 0 | Vvi-Vitvi12g04520\_t001 |  |  |  |  |  |  |  |  |  |  |  |  |
| 0 | Vvi-Vitvi12g04521\_t001 |  |  |  |  |  |  |  |  |  |  |  |  |
| 0 | Vvi-Vitvi12g02678\_t001 |  |  |  |  |  |  |  |  |  |  |  |  |
| 0 | Vvi-Vitvi12g04522\_t001 |  |  |  |  |  |  |  |  |  |  |  |  |
| 0 | Vvi-Vitvi12g04523\_t001 |  |  |  |  |  |  |  |  |  |  |  |  |
| 0 | Vvi-Vitvi12g01921\_t001 |  |  |  |  |  |  |  |  |  |  |  |  |
| 0 | Vvi-Vitvi12g04524\_t001 |  |  |  |  |  |  |  |  |  |  |  |  |
| 0 | Vvi-Vitvi12g01922\_t001 |  |  |  |  |  |  |  |  |  |  |  |  |
| 0 | Vvi-Vitvi12g04525\_t001 |  |  |  |  |  |  |  |  |  |  |  |  |
| 0 | Vvi-Vitvi12g01923\_t001 |  |  |  |  |  |  |  |  |  |  |  |  |
| 0 | Vvi-Vitvi12g01924\_t001 |  |  |  |  |  |  |  |  |  |  |  |  |
| 0 | Vvi-Vitvi12g01926\_t001 |  |  |  |  |  |  |  |  |  |  |  |  |
| 0 | Vvi-Vitvi12g01927\_t001 |  |  |  |  |  |  |  |  |  |  |  |  |
| 0 | Vvi-Vitvi12g01928\_t001 |  |  |  |  |  |  |  |  |  |  |  |  |
| 0 | Vvi-Vitvi12g01929\_t001 |  |  |  |  |  |  |  |  |  |  |  |  |
| 0 | Vvi-Vitvi12g04526\_t001 |  |  |  |  |  |  |  |  |  |  |  |  |
| 0 | Vvi-Vitvi12g02681\_t001 |  |  |  |  |  |  |  |  |  |  |  |  |
| 0 | Vvi-Vitvi12g02682\_t001 |  |  |  |  |  |  |  |  |  |  |  |  |
| 0 | Vvi-Vitvi12g04527\_t001 |  |  |  |  |  |  |  |  |  |  |  |  |
| 0 | Vvi-Vitvi12g04528\_t001 |  |  |  |  |  |  |  |  |  |  |  |  |
| 0 | Vvi-Vitvi12g04529\_t001 |  |  |  |  |  |  |  |  |  |  |  |  |
| 0 | Vvi-Vitvi12g04530\_t001 |  |  |  |  |  |  |  |  |  |  |  |  |
| 0 | Vvi-Vitvi12g01935\_t001 |  |  |  |  |  |  |  |  |  |  |  |  |
| 0 | Vvi-Vitvi12g04531\_t001 |  |  |  |  |  |  |  |  |  |  |  |  |
| 0 | Vvi-Vitvi12g04532\_t001 |  |  |  |  |  |  |  |  |  |  |  |  |
| 0 | Vvi-Vitvi12g01937\_t002 |  |  |  |  |  |  |  |  |  |  |  |  |
| 0 | Vvi-Vitvi12g04533\_t001 |  |  |  |  |  |  |  |  |  |  |  |  |
| 0 | Vvi-Vitvi12g04534\_t001 |  |  |  |  |  |  |  |  |  |  |  |  |
| 0 | Vvi-Vitvi12g04535\_t001 |  |  |  |  |  |  |  |  |  |  |  |  |
| 0 | Vvi-Vitvi12g04536\_t001 |  |  |  |  |  |  |  |  |  |  |  |  |
| 0 | Vvi-Vitvi12g01939\_t001 |  |  |  |  |  |  |  |  |  |  |  |  |
| 0 | Vvi-Vitvi12g04537\_t001 |  |  |  |  |  |  |  |  |  |  |  |  |
| 0 | Vvi-Vitvi12g01940\_t001 |  |  |  |  |  |  |  |  |  |  |  |  |
| 0 | Vvi-Vitvi12g04538\_t001 |  |  |  |  |  |  |  |  |  |  |  |  |
| 0 | Vvi-Vitvi12g04539\_t001 |  |  |  |  |  |  |  |  |  |  |  |  |
| 0 | Vvi-Vitvi12g04540\_t001 |  |  |  |  |  |  |  |  |  |  |  |  |
| 0 | Vvi-Vitvi12g04541\_t001 |  |  |  |  |  |  |  |  |  |  |  |  |
| 0 | Vvi-Vitvi12g04542\_t001 |  |  |  |  |  |  |  |  |  |  |  |  |
| 0 | Vvi-Vitvi12g01944\_t001 |  |  |  |  |  |  |  |  |  |  |  |  |
| 0 | Vvi-Vitvi12g04543\_t001 |  |  |  |  |  |  |  |  |  |  |  |  |
| 0 | Vvi-Vitvi12g04544\_t001 |  |  |  |  |  |  |  |  |  |  |  |  |
| 0 | Vvi-Vitvi12g04545\_t001 |  |  |  |  |  |  |  |  |  |  |  |  |
| 0 | Vvi-Vitvi12g04546\_t001 |  |  |  |  |  |  |  |  |  |  |  |  |
| 0 | Vvi-Vitvi12g04547\_t001 |  |  |  |  |  |  |  |  |  |  |  |  |
| 0 | Vvi-Vitvi12g01950\_t001 |  |  |  |  |  |  |  |  |  |  |  |  |
| 0 | Vvi-Vitvi12g01951\_t001 |  |  |  |  |  |  |  |  |  |  |  |  |
| 0 | Vvi-Vitvi12g04548\_t001 |  |  |  |  |  |  |  |  |  |  |  |  |
| 0 | Vvi-Vitvi12g01954\_t001 |  |  |  |  |  |  |  |  |  |  |  |  |
| 0 | Vvi-Vitvi12g04549\_t001 |  |  |  |  |  |  |  |  |  |  |  |  |
| 0 | Vvi-Vitvi12g01955\_t001 |  |  |  |  |  |  |  |  |  |  |  |  |
| 0 | Vvi-Vitvi12g04550\_t001 |  |  |  |  |  |  |  |  |  |  |  |  |
| 0 | Vvi-Vitvi12g04551\_t001 |  |  |  |  |  |  |  |  |  |  |  |  |
| 0 | Vvi-Vitvi12g01960\_t001 |  |  |  |  |  |  |  |  |  |  |  |  |
| 0 | Vvi-Vitvi12g04552\_t001 |  |  |  |  |  |  |  |  |  |  |  |  |
| 0 | Vvi-Vitvi12g04553\_t001 |  |  |  |  |  |  |  |  |  |  |  |  |
| 0 | Vvi-Vitvi12g01963\_t001 |  |  |  |  |  |  |  |  |  |  |  |  |
| 0 | Vvi-Vitvi12g01964\_t001 |  |  |  |  |  |  |  |  |  |  |  |  |
| 0 | Vvi-Vitvi12g04554\_t001 |  |  |  |  |  |  |  |  |  |  |  |  |
| 0 | Vvi-Vitvi12g01965\_t001 |  |  |  |  |  |  |  |  |  |  |  |  |
| 0 | Vvi-Vitvi12g01968\_t001 |  |  |  |  |  |  |  |  |  |  |  |  |
| 0 | Vvi-Vitvi12g01969\_t001 |  |  |  |  |  |  |  |  |  |  |  |  |
| 0 | Vvi-Vitvi12g04555\_t001 |  |  |  |  |  |  |  |  |  |  |  |  |
| 0 | Vvi-Vitvi12g04556\_t001 |  |  |  |  |  |  |  |  |  |  |  |  |
| 0 | Vvi-Vitvi12g04557\_t001 |  |  |  |  |  |  |  |  |  |  |  |  |
| 0 | Vvi-Vitvi12g04558\_t001 |  |  |  |  |  |  |  |  |  |  |  |  |
| 0 | Vvi-Vitvi12g04559\_t001 |  |  |  |  |  |  |  |  |  |  |  |  |
| 0 | Vvi-Vitvi12g04560\_t001 |  |  |  |  |  |  |  |  |  |  |  |  |
| 0 | Vvi-Vitvi12g01972\_t001 |  |  |  |  |  |  |  |  |  |  |  |  |
| 0 | Vvi-Vitvi12g01973\_t001 |  |  |  |  |  |  |  |  |  |  |  |  |
| 0 | Vvi-Vitvi12g01974\_t001 |  |  |  |  |  |  |  |  |  |  |  |  |
| 0 | Vvi-Vitvi12g02698\_t001 |  |  |  |  |  |  |  |  |  |  |  |  |
| 0 | Vvi-Vitvi12g04561\_t001 |  |  |  |  |  |  |  |  |  |  |  |  |
| 0 | Vvi-Vitvi12g04562\_t001 |  |  |  |  |  |  |  |  |  |  |  |  |
| 0 | Vvi-Vitvi12g01977\_t001 |  |  |  |  |  |  |  |  |  |  |  |  |
| 0 | Vvi-Vitvi12g01979\_t001 |  |  |  |  |  |  |  |  |  |  |  |  |
| 0 | Vvi-Vitvi12g01981\_t001 |  |  |  |  |  |  |  |  |  |  |  |  |
| 0 | Vvi-Vitvi12g02701\_t001 |  |  |  |  |  |  |  |  |  |  |  |  |
| 0 | Vvi-Vitvi12g04563\_t001 |  |  |  |  |  |  |  |  |  |  |  |  |
| 0 | Vvi-Vitvi12g02703\_t001 |  |  |  |  |  |  |  |  |  |  |  |  |
| 0 | Vvi-Vitvi12g04564\_t001 |  |  |  |  |  |  |  |  |  |  |  |  |
| 0 | Vvi-Vitvi12g02705\_t001 |  |  |  |  |  |  |  |  |  |  |  |  |
| 0 | Vvi-Vitvi12g04565\_t001 |  |  |  |  |  |  |  |  |  |  |  |  |
| 0 | Vvi-Vitvi12g04566\_t001 |  |  |  |  |  |  |  |  |  |  |  |  |
| 0 | Vvi-Vitvi12g04567\_t001 |  |  |  |  |  |  |  |  |  |  |  |  |
| 0 | Vvi-Vitvi12g04568\_t001 |  |  |  |  |  |  |  |  |  |  |  |  |
| 0 | Vvi-Vitvi12g04569\_t001 |  |  |  |  |  |  |  |  |  |  |  |  |
| 0 | Vvi-Vitvi12g04570\_t001 |  |  |  |  |  |  |  |  |  |  |  |  |
| 0 | Vvi-Vitvi12g04571\_t001 |  |  |  |  |  |  |  |  |  |  |  |  |
| 0 | Vvi-Vitvi12g01984\_t001 |  |  |  |  |  |  |  |  |  |  |  |  |
| 0 | Vvi-Vitvi12g04572\_t001 |  |  |  |  |  |  |  |  |  |  |  |  |
| 0 | Vvi-Vitvi12g04573\_t001 |  |  |  |  |  |  |  |  |  |  |  |  |
| 0 | Vvi-Vitvi12g04574\_t001 |  |  |  |  |  |  |  |  |  |  |  |  |
| 0 | Vvi-Vitvi12g01987\_t001 |  |  |  |  |  |  |  |  |  |  |  |  |
| 0 | Vvi-Vitvi12g01988\_t001 |  |  |  |  |  |  |  |  |  |  |  |  |
| 0 | Vvi-Vitvi12g02709\_t001 |  |  |  |  |  |  |  |  |  |  |  |  |
| 0 | Vvi-Vitvi12g04575\_t001 |  |  |  |  |  |  |  |  |  |  |  |  |
| 0 | Vvi-Vitvi12g04576\_t001 |  |  |  |  |  |  |  |  |  |  |  |  |
| 0 | Vvi-Vitvi12g04577\_t001 |  |  |  |  |  |  |  |  |  |  |  |  |
| 0 | Vvi-Vitvi12g02714\_t001 |  |  |  |  |  |  |  |  |  |  |  |  |
| 0 | Vvi-Vitvi12g01993\_t001 |  |  |  |  |  |  |  |  |  |  |  |  |
| 0 | Vvi-Vitvi12g01994\_t001 |  |  |  |  |  |  |  |  |  |  |  |  |
| 0 | Vvi-Vitvi12g04578\_t001 |  |  |  |  |  |  |  |  |  |  |  |  |
| 0 | Vvi-Vitvi12g01996\_t002 |  |  |  |  |  |  |  |  |  |  |  |  |
| 0 | Vvi-Vitvi12g01997\_t001 |  |  |  |  |  |  |  |  |  |  |  |  |
| 0 | Vvi-Vitvi12g01998\_t001 |  |  |  |  |  |  |  |  |  |  |  |  |
| 0 | Vvi-Vitvi12g01999\_t001 |  |  |  |  |  |  |  |  |  |  |  |  |
| 0 | Vvi-Vitvi12g02000\_t001 |  |  |  |  |  |  |  |  |  |  |  |  |
| 0 | Vvi-Vitvi12g04579\_t001 |  |  |  |  |  |  |  |  |  |  |  |  |
| 0 | Vvi-Vitvi12g04580\_t001 |  |  |  |  |  |  |  |  |  |  |  |  |
| 0 | Vvi-Vitvi12g02001\_t001 |  |  |  |  |  |  |  |  |  |  |  |  |
| 0 | Vvi-Vitvi12g02003\_t001 |  |  |  |  |  |  |  |  |  |  |  |  |
| 0 | Vvi-Vitvi12g02006\_t001 |  |  |  |  |  |  |  |  |  |  |  |  |
| 0 | Vvi-Vitvi12g02007\_t001 |  |  |  |  |  |  |  |  |  |  |  |  |
| 0 | Vvi-Vitvi12g02009\_t001 |  |  |  |  |  |  |  |  |  |  |  |  |
| 0 | Vvi-Vitvi12g02010\_t001 |  |  |  |  |  |  |  |  |  |  |  |  |
| 0 | Vvi-Vitvi12g04581\_t001 |  |  |  |  |  |  |  |  |  |  |  |  |
| 0 | Vvi-Vitvi12g04582\_t001 |  |  |  |  |  |  |  |  |  |  |  |  |
| 0 | Vvi-Vitvi12g02011\_t001 |  |  |  |  |  |  |  |  |  |  |  |  |
| 0 | Vvi-Vitvi12g02013\_t001 |  |  |  |  |  |  |  |  |  |  |  |  |
| 0 | Vvi-Vitvi12g02014\_t001 |  |  |  |  |  |  |  |  |  |  |  |  |
| 0 | Vvi-Vitvi12g02015\_t001 |  |  |  |  |  |  |  |  |  |  |  |  |
| 0 | Vvi-Vitvi12g02016\_t001 |  |  |  |  |  |  |  |  |  |  |  |  |
| 0 | Vvi-Vitvi12g02017\_t001 |  |  |  |  |  |  |  |  |  |  |  |  |
| 0 | Vvi-Vitvi12g02018\_t001 |  |  |  |  |  |  |  |  |  |  |  |  |
| 0 | Vvi-Vitvi12g02019\_t001 |  |  |  |  |  |  |  |  |  |  |  |  |
| 0 | Vvi-Vitvi12g02020\_t001 |  |  |  |  |  |  |  |  |  |  |  |  |
| 0 | Vvi-Vitvi12g02716\_t001 |  |  |  |  |  |  |  |  |  |  |  |  |
| 0 | Vvi-Vitvi12g02021\_t001 |  |  |  |  |  |  |  |  |  |  |  |  |
| 0 | Vvi-Vitvi12g04583\_t001 |  |  |  |  |  |  |  |  |  |  |  |  |
| 0 | Vvi-Vitvi12g02717\_t001 |  |  |  |  |  |  |  |  |  |  |  |  |
| 0 | Vvi-Vitvi12g02022\_t001 |  |  |  |  |  |  |  |  |  |  |  |  |
| 0 | Vvi-Vitvi12g02023\_t001 |  |  |  |  |  |  |  |  |  |  |  |  |
| 0 | Vvi-Vitvi12g02026\_t001 |  |  |  |  |  |  |  |  |  |  |  |  |
| 0 | Vvi-Vitvi12g02718\_t001 |  |  |  |  |  |  |  |  |  |  |  |  |
| 0 | Vvi-Vitvi12g02027\_t001 |  |  |  |  |  |  |  |  |  |  |  |  |
| 0 | Vvi-Vitvi12g02028\_t001 |  |  |  |  |  |  |  |  |  |  |  |  |
| 0 | Vvi-Vitvi12g04584\_t001 |  |  |  |  |  |  |  |  |  |  |  |  |
| 1 | Vvi-Vitvi12g04585\_t001 |  | Atr-ERN20021 |  |  |  |  |  |  |  |  |  |  |
| 1 | Vvi-Vitvi12g02033\_t001 |  | Atr-ERN20023 |  |  |  |  |  |  |  |  |  |  |
| 1 | Vvi-Vitvi12g02034\_t001 |  | Atr-ERN20028 |  |  |  |  |  |  |  |  |  |  |
| 1 | Vvi-Vitvi12g04586\_t001 |  | \| \| |  |  |  |  |  |  |  |  |  |  |
| 1 | Vvi-Vitvi12g02037\_t001 |  | Atr-ERN20032 |  |  |  |  |  |  |  |  |  |  |
| 1 | Vvi-Vitvi12g04587\_t001 |  | \| \| |  |  |  |  |  |  |  |  |  |  |
| 1 | Vvi-Vitvi12g04588\_t001 |  | \| \| |  |  |  |  |  |  |  |  |  |  |
| 1 | Vvi-Vitvi12g02039\_t001 |  | \| \| |  |  |  |  |  |  |  |  |  |  |
| 1 | Vvi-Vitvi12g04589\_t001 |  | \| \| |  |  |  |  |  |  |  |  |  |  |
| 1 | Vvi-Vitvi12g02040\_t002 |  | \| \| |  |  |  |  |  |  |  |  |  |  |
| 1 | Vvi-Vitvi12g04590\_t001 |  | \| \| |  |  |  |  |  |  |  |  |  |  |
| 1 | Vvi-Vitvi12g02041\_t001 |  | Atr-ERN20036 |  |  |  |  |  |  |  |  |  |  |
| 1 | Vvi-Vitvi12g02042\_t001 |  | Atr-ERN20037 |  |  |  |  |  |  |  |  |  |  |
| 1 | Vvi-Vitvi12g02721\_t001 |  | Atr-ERN20041 |  |  |  |  |  |  |  |  |  |  |
| 1 | Vvi-Vitvi12g02044\_t001 |  | Atr-ERN20043 |  |  |  |  |  |  |  |  |  |  |
| 1 | Vvi-Vitvi12g02045\_t003 |  | Atr-ERN20048 |  |  |  |  |  |  |  |  |  |  |
| 1 | Vvi-Vitvi12g02046\_t001 |  | Atr-ERN20049 |  |  |  |  |  |  |  |  |  |  |
| 1 | Vvi-Vitvi12g02047\_t003 |  | Atr-ERN20051 |  |  |  |  |  |  |  |  |  |  |
| 1 | Vvi-Vitvi12g02050\_t001 |  | Atr-ERN20055 |  |  |  |  |  |  |  |  |  |  |
| 1 | Vvi-Vitvi12g02723\_t002 |  | Atr-ERN20057 |  |  |  |  |  |  |  |  |  |  |
| 1 | Vvi-Vitvi12g02055\_t003 |  | \| \| |  |  |  |  |  |  |  |  |  |  |
| 1 | Vvi-Vitvi12g02056\_t001 |  | Atr-ERN20073 |  |  |  |  |  |  |  |  |  |  |
| 1 | Vvi-Vitvi12g02057\_t001 |  | Atr-ERN20074 |  |  |  |  |  |  |  |  |  |  |
| 0 | Vvi-Vitvi12g02058\_t001 |  |  |  |  |  |  |  |  |  |  |  |  |
| 0 | Vvi-Vitvi12g04591\_t001 |  |  |  |  |  |  |  |  |  |  |  |  |
| 1 | Vvi-Vitvi12g02059\_t001 |  | Atr-ERM99646 |  |  |  |  |  |  |  |  |  |  |
| 1 | Vvi-Vitvi12g02060\_t001 |  | Atr-ERM99657 |  |  |  |  |  |  |  |  |  |  |
| 1 | Vvi-Vitvi12g04592\_t001 |  | \| \| |  |  |  |  |  |  |  |  |  |  |
| 1 | Vvi-Vitvi12g02061\_t001 |  | Atr-ERM99675 |  |  |  |  |  |  |  |  |  |  |
| 1 | Vvi-Vitvi12g04593\_t001 |  | \| \| |  |  |  |  |  |  |  |  |  |  |
| 1 | Vvi-Vitvi12g02062\_t001 |  | Atr-ERM99676 |  |  |  |  |  |  |  |  |  |  |
| 1 | Vvi-Vitvi12g02063\_t001 |  | Atr-ERM99678 |  |  |  |  |  |  |  |  |  |  |
| 1 | Vvi-Vitvi12g02064\_t001 |  | Atr-ERM99687 |  |  |  |  |  |  |  |  |  |  |
| 1 | Vvi-Vitvi12g02724\_t001 |  | \| \| |  |  |  |  |  |  |  |  |  |  |
| 1 | Vvi-Vitvi12g04594\_t001 |  | \| \| |  |  |  |  |  |  |  |  |  |  |
| 1 | Vvi-Vitvi12g04595\_t001 |  | \| \| |  |  |  |  |  |  |  |  |  |  |
| 1 | Vvi-Vitvi12g04596\_t001 |  | \| \| |  |  |  |  |  |  |  |  |  |  |
| 1 | Vvi-Vitvi12g04597\_t001 |  | \| \| |  |  |  |  |  |  |  |  |  |  |
| 1 | Vvi-Vitvi12g04598\_t001 |  | \| \| |  |  |  |  |  |  |  |  |  |  |
| 1 | Vvi-Vitvi12g02727\_t001 |  | \| \| |  |  |  |  |  |  |  |  |  |  |
| 1 | Vvi-Vitvi12g02066\_t001 |  | \| \| |  |  |  |  |  |  |  |  |  |  |
| 1 | Vvi-Vitvi12g04599\_t001 |  | \| \| |  |  |  |  |  |  |  |  |  |  |
| 1 | Vvi-Vitvi12g02729\_t002 |  | \| \| |  |  |  |  |  |  |  |  |  |  |
| 1 | Vvi-Vitvi12g04600\_t001 |  | \| \| |  |  |  |  |  |  |  |  |  |  |
| 1 | Vvi-Vitvi12g04601\_t001 |  | \| \| |  |  |  |  |  |  |  |  |  |  |
| 1 | Vvi-Vitvi12g04602\_t001 |  | \| \| |  |  |  |  |  |  |  |  |  |  |
| 1 | Vvi-Vitvi12g04603\_t001 |  | \| \| |  |  |  |  |  |  |  |  |  |  |
| 1 | Vvi-Vitvi12g02732\_t001 |  | \| \| |  |  |  |  |  |  |  |  |  |  |
| 1 | Vvi-Vitvi12g02071\_t001 |  | \| \| |  |  |  |  |  |  |  |  |  |  |
| 1 | Vvi-Vitvi12g02072\_t002 |  | \| \| |  |  |  |  |  |  |  |  |  |  |
| 1 | Vvi-Vitvi12g04604\_t001 |  | \| \| |  |  |  |  |  |  |  |  |  |  |
| 1 | Vvi-Vitvi12g02073\_t001 |  | \| \| |  |  |  |  |  |  |  |  |  |  |
| 1 | Vvi-Vitvi12g02074\_t002 |  | \| \| |  |  |  |  |  |  |  |  |  |  |
| 1 | Vvi-Vitvi12g04605\_t001 |  | Atr-ERM99698 |  |  |  |  |  |  |  |  |  |  |
| 0 | Vvi-Vitvi12g02076\_t001 |  |  |  |  |  |  |  |  |  |  |  |  |
| 0 | Vvi-Vitvi12g02078\_t001 |  |  |  |  |  |  |  |  |  |  |  |  |
| 0 | Vvi-Vitvi12g04606\_t001 |  |  |  |  |  |  |  |  |  |  |  |  |
| 0 | Vvi-Vitvi12g04607\_t001 |  |  |  |  |  |  |  |  |  |  |  |  |
| 0 | Vvi-Vitvi12g04608\_t001 |  |  |  |  |  |  |  |  |  |  |  |  |
| 0 | Vvi-Vitvi12g04609\_t001 |  |  |  |  |  |  |  |  |  |  |  |  |
| 0 | Vvi-Vitvi12g02735\_t001 |  |  |  |  |  |  |  |  |  |  |  |  |
| 0 | Vvi-Vitvi12g02736\_t001 |  |  |  |  |  |  |  |  |  |  |  |  |
| 0 | Vvi-Vitvi12g02737\_t001 |  |  |  |  |  |  |  |  |  |  |  |  |
| 0 | Vvi-Vitvi12g04610\_t001 |  |  |  |  |  |  |  |  |  |  |  |  |
| 0 | Vvi-Vitvi12g04611\_t001 |  |  |  |  |  |  |  |  |  |  |  |  |
| 0 | Vvi-Vitvi12g02738\_t001 |  |  |  |  |  |  |  |  |  |  |  |  |
| 0 | Vvi-Vitvi12g02739\_t001 |  |  |  |  |  |  |  |  |  |  |  |  |
| 0 | Vvi-Vitvi12g04612\_t001 |  |  |  |  |  |  |  |  |  |  |  |  |
| 0 | Vvi-Vitvi12g04613\_t001 |  |  |  |  |  |  |  |  |  |  |  |  |
| 0 | Vvi-Vitvi12g04614\_t001 |  |  |  |  |  |  |  |  |  |  |  |  |
| 0 | Vvi-Vitvi12g04615\_t001 |  |  |  |  |  |  |  |  |  |  |  |  |
| 0 | Vvi-Vitvi12g04616\_t001 |  |  |  |  |  |  |  |  |  |  |  |  |
| 0 | Vvi-Vitvi12g02743\_t001 |  |  |  |  |  |  |  |  |  |  |  |  |
| 0 | Vvi-Vitvi12g02744\_t001 |  |  |  |  |  |  |  |  |  |  |  |  |
| 0 | Vvi-Vitvi12g04617\_t001 |  |  |  |  |  |  |  |  |  |  |  |  |
| 0 | Vvi-Vitvi12g04618\_t001 |  |  |  |  |  |  |  |  |  |  |  |  |
| 0 | Vvi-Vitvi12g04619\_t001 |  |  |  |  |  |  |  |  |  |  |  |  |
| 0 | Vvi-Vitvi12g04620\_t001 |  |  |  |  |  |  |  |  |  |  |  |  |
| 0 | Vvi-Vitvi12g04621\_t001 |  |  |  |  |  |  |  |  |  |  |  |  |
| 0 | Vvi-Vitvi12g02085\_t001 |  |  |  |  |  |  |  |  |  |  |  |  |
| 0 | Vvi-Vitvi12g02088\_t001 |  |  |  |  |  |  |  |  |  |  |  |  |
| 0 | Vvi-Vitvi12g04622\_t001 |  |  |  |  |  |  |  |  |  |  |  |  |
| 0 | Vvi-Vitvi12g04623\_t001 |  |  |  |  |  |  |  |  |  |  |  |  |
| 0 | Vvi-Vitvi12g02745\_t001 |  |  |  |  |  |  |  |  |  |  |  |  |
| 0 | Vvi-Vitvi12g04624\_t001 |  |  |  |  |  |  |  |  |  |  |  |  |
| 0 | Vvi-Vitvi12g04625\_t001 |  |  |  |  |  |  |  |  |  |  |  |  |
| 0 | Vvi-Vitvi12g02094\_t001 |  |  |  |  |  |  |  |  |  |  |  |  |
| 0 | Vvi-Vitvi12g02097\_t001 |  |  |  |  |  |  |  |  |  |  |  |  |
| 0 | Vvi-Vitvi12g02099\_t001 |  |  |  |  |  |  |  |  |  |  |  |  |
| 0 | Vvi-Vitvi12g04626\_t001 |  |  |  |  |  |  |  |  |  |  |  |  |
| 0 | Vvi-Vitvi12g04627\_t001 |  |  |  |  |  |  |  |  |  |  |  |  |
| 0 | Vvi-Vitvi12g04628\_t001 |  |  |  |  |  |  |  |  |  |  |  |  |
| 0 | Vvi-Vitvi12g02101\_t001 |  |  |  |  |  |  |  |  |  |  |  |  |
| 0 | Vvi-Vitvi12g04629\_t001 |  |  |  |  |  |  |  |  |  |  |  |  |
| 0 | Vvi-Vitvi12g02753\_t001 |  |  |  |  |  |  |  |  |  |  |  |  |
| 0 | Vvi-Vitvi12g04630\_t001 |  |  |  |  |  |  |  |  |  |  |  |  |
| 0 | Vvi-Vitvi12g02754\_t001 |  |  |  |  |  |  |  |  |  |  |  |  |
| 0 | Vvi-Vitvi12g02103\_t001 |  |  |  |  |  |  |  |  |  |  |  |  |
| 0 | Vvi-Vitvi12g02105\_t001 |  |  |  |  |  |  |  |  |  |  |  |  |
| 1 | Vvi-Vitvi12g02106\_t001 |  | Atr-ERM99705 |  |  |  |  |  |  |  |  |  |  |
| 1 | Vvi-Vitvi12g02109\_t001 |  | \| \| |  |  |  |  |  |  |  |  |  |  |
| 1 | Vvi-Vitvi12g02110\_t001 |  | \| \| |  |  |  |  |  |  |  |  |  |  |
| 1 | Vvi-Vitvi12g02111\_t001 |  | \| \| |  |  |  |  |  |  |  |  |  |  |
| 1 | Vvi-Vitvi12g02115\_t001 |  | Atr-ERM99712 |  |  |  |  |  |  |  |  |  |  |
| 1 | Vvi-Vitvi12g04631\_t001 |  | \| \| |  |  |  |  |  |  |  |  |  |  |
| 1 | Vvi-Vitvi12g04632\_t001 |  | \| \| |  |  |  |  |  |  |  |  |  |  |
| 1 | Vvi-Vitvi12g02119\_t001 |  | \| \| |  |  |  |  |  |  |  |  |  |  |
| 1 | Vvi-Vitvi12g02120\_t001 |  | \| \| |  |  |  |  |  |  |  |  |  |  |
| 1 | Vvi-Vitvi12g02121\_t001 |  | Atr-ERM99729 |  |  |  |  |  |  |  |  |  |  |
| 1 | Vvi-Vitvi12g04633\_t001 |  | \| \| |  |  |  |  |  |  |  |  |  |  |
| 1 | Vvi-Vitvi12g02122\_t001 |  | \| \| |  |  |  |  |  |  |  |  |  |  |
| 1 | Vvi-Vitvi12g04634\_t001 |  | \| \| |  |  |  |  |  |  |  |  |  |  |
| 1 | Vvi-Vitvi12g04635\_t001 |  | \| \| |  |  |  |  |  |  |  |  |  |  |
| 1 | Vvi-Vitvi12g04636\_t001 |  | \| \| |  |  |  |  |  |  |  |  |  |  |
| 1 | Vvi-Vitvi12g02124\_t001 |  | Atr-ERM99746 |  |  |  |  |  |  |  |  |  |  |
| 1 | Vvi-Vitvi12g02125\_t001 |  | Atr-ERM99748 |  |  |  |  |  |  |  |  |  |  |
| 1 | Vvi-Vitvi12g02126\_t001 |  | Atr-ERM99754 |  |  |  |  |  |  |  |  |  |  |
| 1 | Vvi-Vitvi12g02128\_t001 |  | Atr-ERM99760 |  |  |  |  |  |  |  |  |  |  |
| 1 | Vvi-Vitvi12g04637\_t001 |  | \| \| |  |  |  |  |  |  |  |  |  |  |
| 1 | Vvi-Vitvi12g02129\_t001 |  | \| \| |  |  |  |  |  |  |  |  |  |  |
| 1 | Vvi-Vitvi12g02130\_t001 |  | Atr-ERM99775 |  |  |  |  |  |  |  |  |  |  |
| 1 | Vvi-Vitvi12g02134\_t002 |  | Atr-ERM99776 |  |  |  |  |  |  |  |  |  |  |
| 1 | Vvi-Vitvi12g02135\_t001 |  | Atr-ERM99777 |  |  |  |  |  |  |  |  |  |  |
| 1 | Vvi-Vitvi12g04638\_t001 |  | \| \| |  |  |  |  |  |  |  |  |  |  |
| 1 | Vvi-Vitvi12g02136\_t001 |  | Atr-ERM99778 |  |  |  |  |  |  |  |  |  |  |
| 1 | Vvi-Vitvi12g02137\_t001 |  | \| \| |  |  |  |  |  |  |  |  |  |  |
| 1 | Vvi-Vitvi12g02138\_t001 |  | Atr-ERM99779 |  |  |  |  |  |  |  |  |  |  |
| 1 | Vvi-Vitvi12g02139\_t001 |  | Atr-ERM99781 |  |  |  |  |  |  |  |  |  |  |
| 1 | Vvi-Vitvi12g02140\_t001 |  | Atr-ERM99784 |  |  |  |  |  |  |  |  |  |  |
| 1 | Vvi-Vitvi12g02141\_t001 |  | Atr-ERM99786 |  |  |  |  |  |  |  |  |  |  |
| 1 | Vvi-Vitvi12g02142\_t001 |  | Atr-ERM99788 |  |  |  |  |  |  |  |  |  |  |
| 1 | Vvi-Vitvi12g02143\_t001 |  | \| \| |  |  |  |  |  |  |  |  |  |  |
| 1 | Vvi-Vitvi12g02144\_t001 |  | Atr-ERM99796 |  |  |  |  |  |  |  |  |  |  |
| 1 | Vvi-Vitvi12g02145\_t002 |  | \| \| |  |  |  |  |  |  |  |  |  |  |
| 1 | Vvi-Vitvi12g02147\_t001 |  | \| \| |  |  |  |  |  |  |  |  |  |  |
| 1 | Vvi-Vitvi12g04639\_t001 |  | \| \| |  |  |  |  |  |  |  |  |  |  |
| 2 | Vvi-Vitvi12g02150\_t001 |  | \| \| |  | Atr-ERM99809 |  |  |  |  |  |  |  |  |
| 2 | Vvi-Vitvi12g02152\_t001 |  | \| \| |  | Atr-ERM99808 |  |  |  |  |  |  |  |  |
| 2 | Vvi-Vitvi12g04640\_t001 |  | \| \| |  | \| \| |  |  |  |  |  |  |  |  |
| 2 | Vvi-Vitvi12g02154\_t001 |  | \| \| |  | \| \| |  |  |  |  |  |  |  |  |
| 2 | Vvi-Vitvi12g04641\_t001 |  | \| \| |  | \| \| |  |  |  |  |  |  |  |  |
| 2 | Vvi-Vitvi12g02157\_t001 |  | \| \| |  | \| \| |  |  |  |  |  |  |  |  |
| 2 | Vvi-Vitvi12g02158\_t001 |  | Atr-ERM99807 |  | \| \| |  |  |  |  |  |  |  |  |
| 1 | Vvi-Vitvi12g02755\_t001 |  |  |  | Atr-ERM99806 |  |  |  |  |  |  |  |  |
| 1 | Vvi-Vitvi12g04642\_t001 |  |  |  | \| \| |  |  |  |  |  |  |  |  |
| 1 | Vvi-Vitvi12g02757\_t001 |  |  |  | Atr-ERM99804 |  |  |  |  |  |  |  |  |
| 1 | Vvi-Vitvi12g02758\_t001 |  |  |  | Atr-ERM99802 |  |  |  |  |  |  |  |  |
| 1 | Vvi-Vitvi12g02159\_t001 |  |  |  | \| \| |  |  |  |  |  |  |  |  |
| 1 | Vvi-Vitvi12g02160\_t001 |  |  |  | \| \| |  |  |  |  |  |  |  |  |
| 1 | Vvi-Vitvi12g02162\_t001 |  |  |  | Atr-ERM99798 |  |  |  |  |  |  |  |  |
| 0 | Vvi-Vitvi12g04643\_t001 |  |  |  |  |  |  |  |  |  |  |  |  |
| 0 | Vvi-Vitvi12g04644\_t001 |  |  |  |  |  |  |  |  |  |  |  |  |
| 0 | Vvi-Vitvi12g02167\_t001 |  |  |  |  |  |  |  |  |  |  |  |  |
| 0 | Vvi-Vitvi12g02168\_t001 |  |  |  |  |  |  |  |  |  |  |  |  |
| 0 | Vvi-Vitvi12g04645\_t003 |  |  |  |  |  |  |  |  |  |  |  |  |
| 0 | Vvi-Vitvi12g04646\_t001 |  |  |  |  |  |  |  |  |  |  |  |  |
| 0 | Vvi-Vitvi12g04647\_t001 |  |  |  |  |  |  |  |  |  |  |  |  |
| 0 | Vvi-Vitvi12g02174\_t003 |  |  |  |  |  |  |  |  |  |  |  |  |
| 0 | Vvi-Vitvi12g02175\_t001 |  |  |  |  |  |  |  |  |  |  |  |  |
| 0 | Vvi-Vitvi12g04648\_t001 |  |  |  |  |  |  |  |  |  |  |  |  |
